# Chromenylium Star Polymers: Merging Water Solubility and Stealth Properties with Shortwave Infrared Emissive Fluorophores

**DOI:** 10.1021/acscentsci.4c01570

**Published:** 2024-12-21

**Authors:** Emily B. Mobley, Eric Y. Lin, Ellen M. Sletten

**Affiliations:** † Department of Chemistry and Biochemistry, University of California, Los Angeles, Los Angeles, California 90095, United States

## Abstract

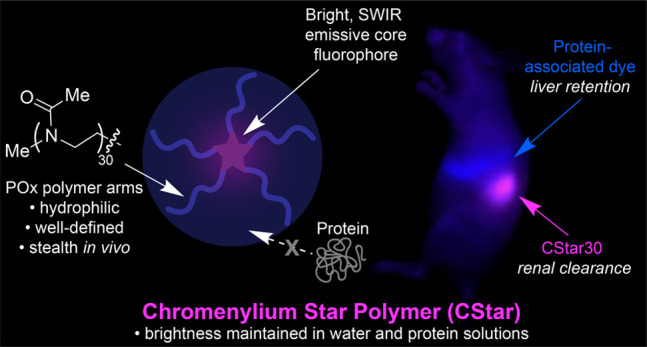

Fluorescence imaging in the shortwave infrared (SWIR) region has emerged as a vital tool for studying mammals. SWIR emissive polymethine dyes are well-suited to this endeavor; however, advancing *in vivo* imaging utility with these dyes is primarily limited by hydrophobicity and/or nonspecific protein association. Herein, we take a distinct approach to combine hydrophilicity and stealth behavior to construct bright, SWIR emissive chromenylium fluorophores by employing a well-defined poly(2-methyl-2-oxazoline) (POx) star polymer architecture, which we refer to as chromenylium stars, or “CStars.” Of these polymer-shielded dyes, the variant containing five POx chains (**CStar30**) boasts particularly enhanced aqueous solubility and SWIR brightness, enabling high-resolution SWIR imaging in mice. The swift renal clearance and stealth behavior displayed *in vivo* also achieves improved noninvasive visualization of the lymphatic system. Further, CStar’s orthogonal biodistribution to an FDA-approved dye, indocyanine green (**ICG**), facilitates excitation-multiplexed SWIR imaging in two colors to achieve simultaneous visualization of both fluid dynamics and protein dynamics in the same animal in real time at video-rate frame counts.

## Abstract 2

Chromenylium star (CStar) polymers are bright, SWIR emissive, and water soluble. Appending stealth polymers surmounts solubility and *in vivo* retention issues of small molecule fluorophore analogs.

## Introduction

Fluorophores are invaluable chemical tools for illuminating biological systems from research to clinical settings. In research, fluorescence imaging is a ubiquitous approach to label biological structures, and quantify biomolecule expression and localization.^1^ There are a plethora of fluorophores available for these studies in cells, transparent organisms, and even small mammals.^2^ Clinically, fluorescence imaging aids in tumor resection,^3,4^ noninvasive visualization of biological processes,^5,6^ and mapping the lymphatic system.^7^ Unlike in research, the clinical optical imaging toolbox is sparse, which is partially attributed to the challenge of gaining FDA approval. Since the 1950s, indocyanine green (**ICG**), a heptamethine cyanine dye that emits primarily in the near-infrared (NIR or NIR-I, 700–1000 nm) region, has been the most widely used contrast agent for optical imaging in humans.^3−7^ Excitingly, a new NIR emitting dye with improved utility emerged from the clinical pipeline in 2021.^8^

Recently, data from preclinical models as well as clinical trials have indicated that moving to the longer, lower energy wavelengths of the shortwave infrared (SWIR or NIR-II, 1000–2000 nm) region results in enhanced contrast, resolution, and penetration of light through tissue.^4,9−11^ Translating these advantages to the clinic would expand our ability to diagnose and treat pervasive disease states with increased sensitivity. Despite an explosion of effort over the past decade designing SWIR emitting fluorophores and technologies,^6^ optimization is still necessary to bring these advantages to the clinic.

**Figure 1:**
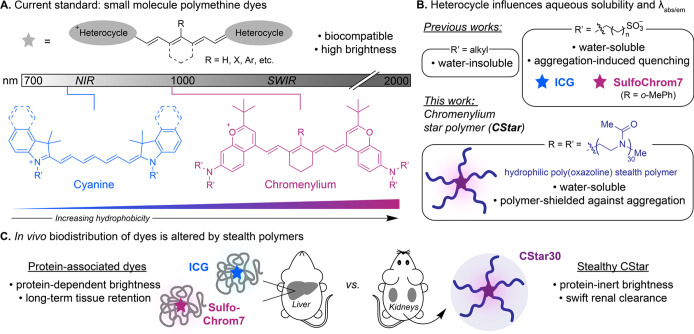
Polymethine fluorophores for *in vivo* imaging. (A) General heptamethine dye structures and properties. (B) Previous work: NIR and SWIR emitting fluorophores and properties according to heterocycle substituents. This work: SWIR emitting chromenylium star polymers and properties. (C) Comparison of *in vivo* biodistribution patterns between protein-associated dyes **ICG** and **SulfoChrom7** (see Figure S1 for structures) and polymer-shielded **CStar30** (see Figure S3 for structure).

To produce SWIR dyes suitable for migration from fundamental science to clinical settings, several metrics must be achieved: (1) biocompatibility, (2) high SWIR brightness, and (3) aqueous solubility.^12^ Stealth behavior (i.e., minimal interaction with biomolecules) in biological settings can also increase stability and open the door for control over *in vivo* localizations.^13^ Of the array of SWIR emitters available, small molecule organic polymethine fluorophores (defined as two heterocycles linked by a polymethine chain) stand out for their nontoxic nature and privileged brightness (Figure 1A).^14−16^ This is due to their favorable photophysical properties, such as high absorption coefficients (ε_max_) and quantum yields of fluorescence (Φ_F_) in organic solvents. However, imaging *in vivo* requires translating brightness to the aqueous biological environment. SWIR emitting polymethine fluorophores are much more difficult to solubilize in water than their NIR emitting analogs, owed to the more conjugated and hydrophobic heterocycles forming nonemissive, blue-shifted aggregates in aqueous media (Figure 1B).^17−19^ Water also inherently quenches emission through −OH bond vibrations.^20^ Furthermore, the small HOMO–LUMO gap required for SWIR emission leads to these fluorophores being more prone to degradation by water and other nucleophiles.^17,21,22^ Typically, polymer-based noncovalent delivery vehicles (e.g., micelles, liposomes) are used to protect SWIR dyes and allow for imaging *in vivo*.^23,24^ Still, nonemissive aggregation is a major problem with these systems, along with fluorophore leaching, stability concerns, nonspecific interactions, and retention *in vivo*.^25^

Methods to overcome the aggregation, brightness, and biological interaction challenges of SWIR emissive dyes in aqueous environments are paramount for clinical translation. Toward these goals, we recently reported hydrophilic versions of heptamethine chromenylium dyes, such as **SulfoChrom7** (λ_max, abs_ 963 nm, fetal bovine serum [FBS]),^26^ which can be considered a SWIR emissive version of **ICG** (λ_max, abs_ 798 nm, FBS) (Figure S1). Both dyes have enabled high-resolution, carrier-free imaging *in vivo*, but **ICG** requires substantially higher concentrations for adequate SWIR detection than **SulfoChrom7**.^3,26−28^

To facilitate *in vivo* imaging, both **SulfoChrom7** and **ICG** are captured by endogenous proteins such as albumin. This interaction maintains a monomeric, emissive state of the fluorophore via steric occlusion, leading to significantly enhanced Φ_F_ in serum and protein solutions (i.e., FBS).^29,30^ In fact, **SulfoChrom7** is primarily aggregated and quenched in protein-free solutions, such as water, and both dyes display poor stability in aqueous environments, regardless of protein association. While striking vasculature imaging has been achieved with these dyes, biodistribution dynamics and clearance rates are dictated by the protein.^31,32^ Thus, protein association limits the ability to modulate specific fluorophore localization and biodistribution *in vivo*.

Protein association is also a significant challenge for the nanomaterials community. In this field, covalent installation of “stealth” polymers is a popular strategy to alleviate *in vivo* interactions.^33,34^ Stealth polymers are neutral, hydrophilic, and flexible, which provides a high entropic barrier to cohesion of biomolecules in solution. In addition to minimizing protein interactions, stealth polymers also impart hydrophilicity to increase bioavailability and steric protection to enhance stability. While poly(ethylene glycol) (PEG) is a popular class of stealth polymers,^34,35^ poly(2-methyl-2-oxazoline) (POx) polymers offer similar stealth properties.^33,36,37^ POx polymers also offer increased hydrophilicity,^38^ sites for functionalization and synthetic control to achieve a well-defined molecular species (Figure S2).^39^

Covalent polymer functionalization is an underutilized approach for specifically improving the biophysical properties of both NIR and SWIR emissive dyes. Typically, fluorophore-polymer conjugates involve a fluorophore appended to the periphery of a polymer-based therapeutic.^40−43^ Notable exceptions include work by Smith and co-workers, who demonstrated with NIR emitting cyanine dyes that short PEG oligomers can be strategically placed to shield the fluorophore, increasing stability in aqueous environments.^44^ Zhou and co-workers took this strategy one step further and placed cyanine dyes at the center of poly(lysine) dendrimers, demonstrating improvements in both brightness and stability of these fluorophores in water.^45^ However, the highly cationic nature of these dendrimers introduced biocompatibility concerns. While no SWIR polymethine dyes have invoked stealth polymers to enhance fluorophore stability, aqueous solubility, or brightness, other scaffolds such as xanthene,^46^ BODIPY,^47^ and donor–acceptor–donor^48^ fluorophores have achieved at least one of these metrics using this strategy.

In this work, we merge the advantages of stealth polymers with SWIR emissive polymethine fluorophores. To keep the contrast agents as molecularly discrete as possible, we adopt a well-defined star polymer architecture. With three or more polymer chains (“arms”) extending from a common core, star polymers are known for their unique properties such as reduced chain entanglements and solution viscosities compared to analogous linear polymers.^43^ Through implementing a SWIR emissive fluorophore at the core of a star polymer, we reasoned the polymer arms would provide water solubility and stealth properties *in vivo*, while also acting as a molecular shield to protect the fluorophore in aqueous environments. Herein, we introduce chromenylium star polymers, or “CStars,” which feature a bright chromenylium fluorophore core with stealthy, hydrophilic POx polymer arms extending outward (Figure 1B). CStars are ultimately applied to excitation-multiplexed two-color SWIR imaging in mice in tandem with **ICG** to reveal how minimizing protein association alters biodistribution and clearance rates *in vivo* (Figure 1C).

## Results and Discussion

### CStar Design

**Figure 2:**
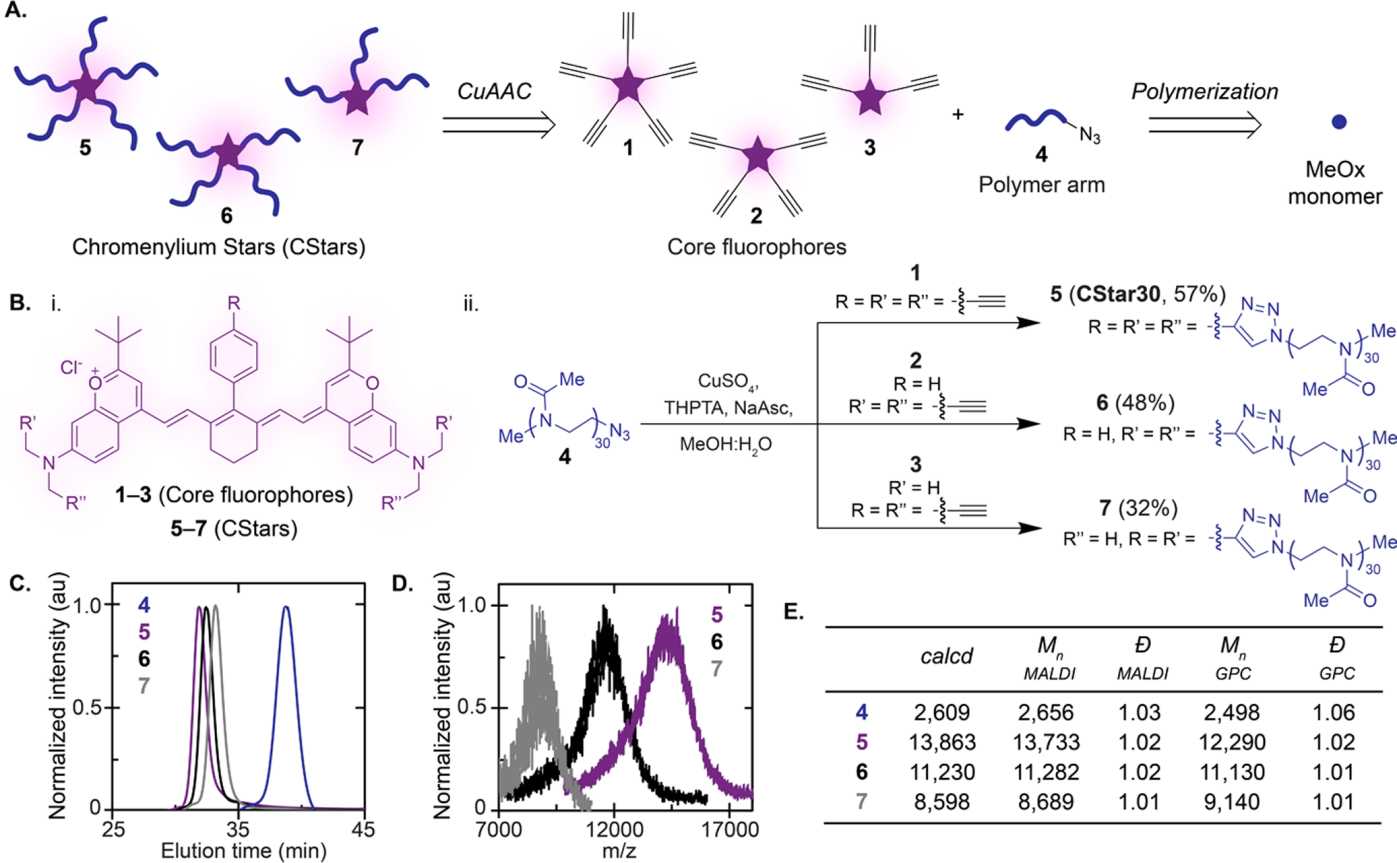
Synthesis of chromenylium star polymers (CStars) via click chemistry. (A) Schematized retrosynthesis of CStars. (B) Structures and syntheses of CStars (i.) Base chromenylium structure of core fluorophores **1**–**3** and CStars **5**–**7** with R groups defined in (ii). (ii.) Copper-catalyzed azide–alkyne cycloaddition (CuAAC) between azide-terminated POx polymer **4** and alkyne-containing dyes **1**, **2**, or **3** (core fluorophores) to yield CStars **5**, **6**, or 7, respectively (see Figure S3, Scheme S4 for structures). (C) Gel permeation chromatography (GPC, dUV 210 nm) of CStars **5**–**7**, and linear POx polymer **4**. (D) Matrix-assisted laser desorption ionization mass spectrometry (MALDI) of CStars **5**–**7**, and linear POx polymer **4**. (E) Table of size parameters calculated (calcd) and observed (GPC, MALDI) for CStars **5**–**7**, and linear POx polymer **4**. *Đ* = *M*_*w*_*/M*_*n*__._ Note that GPC data were obtained via comparison of retention times to linear POx standards, which are unoptimized for the star polymer architecture (see Appendix B).

In pursuit of CStars, a heptamethine chromenylium fluorophore (Chrom7) was chosen as the core for its respectable SWIR brightness.^15^ We structurally amended the core chromenylium scaffold to contain three to five terminal alkynes for polymer couplings via copper-catalyzed click chemistry. For the stealthy polymer arms, we selected azide-functionalized linear POx, polymerized from the corresponding 2-methyl-2-oxazoline (MeOx) monomer. We focused on a degree of polymerization of 30, as (MeOx)_30_ polymers have demonstrated excellent hydrophilicity in amphiphiles composing micelles and nanoemulsions.^49,50^ Bringing the core fluorophore and polymer arms together with a “coupling-onto” approach, where each component is first prepared independently, favors star polymers with low dispersity, resulting in a well-defined molecular species (Figure 2A).^43^ Although the kinetics of large polymer–polymer couplings are sluggish, copper-catalyzed azide–alkyne cycloaddition (CuAAC) has been demonstrated to be efficient for this purpose.^51,52^

### CStar Synthesis

To first explore how a star polymer architecture influences the properties of chromenylium SWIR dyes, three core fluorophores with varying numbers of alkynes were prepared. These core fluorophores were accessed through four common precursors: heptamethine linkers **S1** and **S2** (Scheme S1) and chromenylium heterocycles **S7** and **S10** (Scheme S2, S3). Briefly, the appropriate heterocycle and linker pair were condensed in the presence of base and acetic anhydride to access penta- (**1**), tetra- (**2**), and trialkyne (**3**) functionalized heptamethine chromenylium dyes (Scheme S4). Separately, a linear POx polymer (**4**) was synthesized through a living cationic ring-opening polymerization of MeOx monomer, initiated with methyl triflate and terminated with sodium azide as a complementary click chemistry handle to the core fluorophores (Scheme S5). POx polymer **4** was found to have an average degree of polymerization of 30 and was well-defined with a low dispersity (*Đ*) of 1.06.

CStars were then synthesized via CuAAC, which cleanly afforded low dispersity (*Đ* ≤ 1.02) CStars (**5**, **6**, and **7**) with either five, four, or three POx polymer arms, respectively (Figure 2B, Figure S3). Optimizing these reaction conditions with **5**, we found that reactions proceed to full conversion in just a few hours with a minimum of two equiv. POx polymer per alkyne and 16% Cu(I) catalyst loading (Figure S4). Size analysis of **5**, **6**, and **7** via gel permeation chromatography (GPC), matrix-assisted laser desorption ionization mass spectrometry (MALDI) (Figure 2C-E), and SDS-PAGE (Figure S5) indicated efficient coupling chemistry was achieved, with excellent reproducibility comparing replicate reactions of **5**, **6**, and **7** via GPC (Figure S6, S7). Isolated yields decreased with decreasing number of arms on CStars due to the increased difficulty of separating species with more similar molecular weights by size exclusion chromatography and dialysis.

### Photophysical Properties and Aqueous Solubility

**Figure 3:**
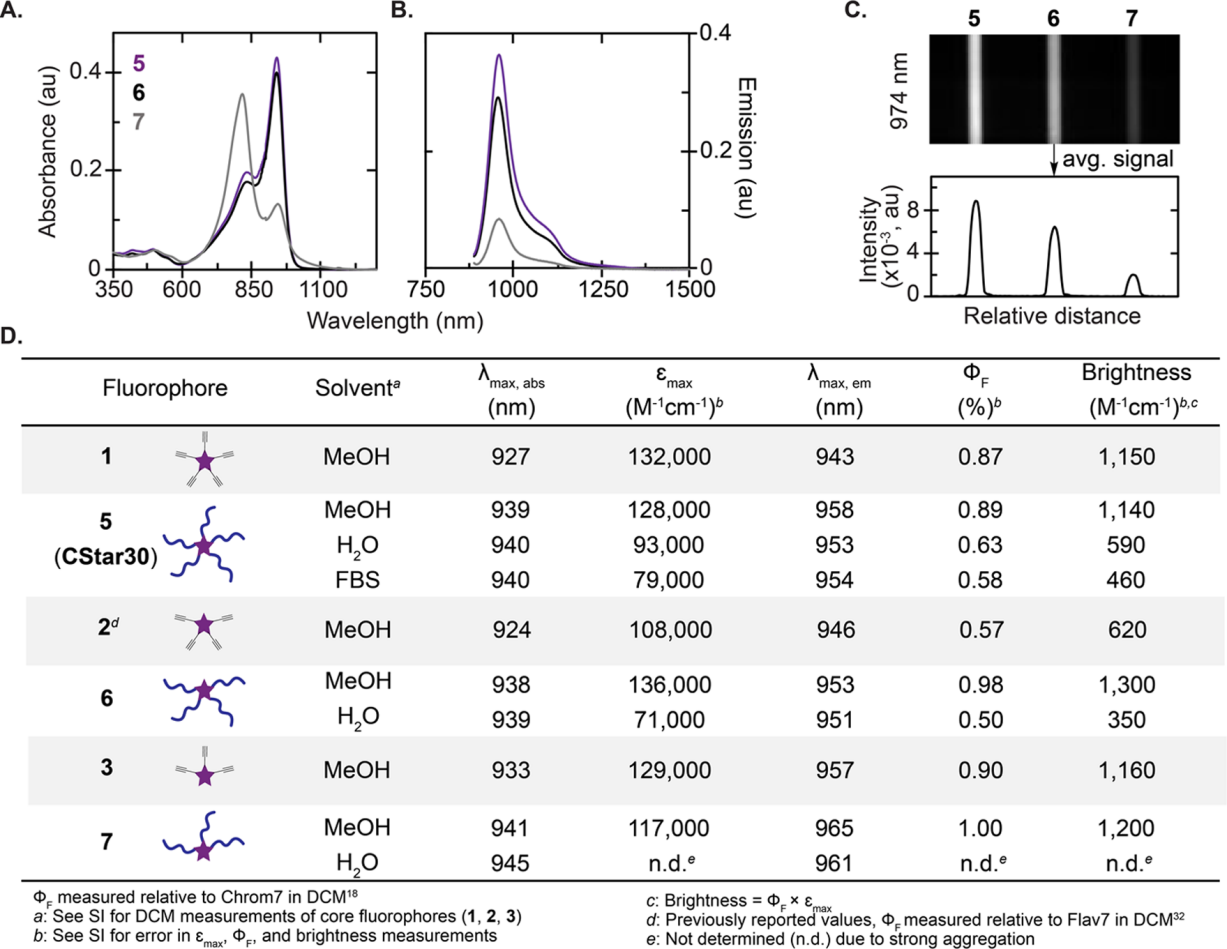
Photophysical properties of CStars and core fluorophores. (A–B) Absorbance (A) and emission (B) spectra of CStars **5**–**7** in water (15 μM, 3 mm cuvette). Emission spectra: 860 nm ex, 885–1500 nm em. (C) Capillary brightness and intensity quantification of samples from (A) measured via InGaAs camera (974 nm ex, 100 mW/cm^2^, 5 ms ET, 1100 nm LP). (D) Table summarizing photophysical measurements of CStars and core fluorophores. See Table S1 for DCM measurements and error values.

With five-, four-, and three-arm CStars (**5**–**7**) in hand, we investigated the photophysical properties and aqueous solubility compared to the core fluorophores (**1**–**3**, Figure 3). Initially, we discovered all CStars are insoluble in dichloromethane (DCM), an organic solvent typically used to benchmark small molecule fluorophore brightness *in vitro*.^19,22^ To accommodate these substantial changes in solubility, a more polar solvent, methanol (MeOH), was selected to directly compare core fluorophore and CStar brightness. Gratifyingly, all CStars displayed typical polymethine absorption/emission profiles with a ∼ 10 nm bathochromic shift (λ_max, abs_ ∼ 940 nm) compared to the corresponding core fluorophore (Figure S8, S9). The brightness (Φ_F_ × ε_max_) of the core fluorophores was also maintained or improved across all CStars (Figure 3D, Table S1). Taken together, these data suggest that the polymer arms are able to alter the solubility of a core fluorophore while preserving desirable photophysical properties.

We then evaluated the CStar architecture’s ability to prevent nonemissive aggregation and translate brightness to water. Initially, we discovered concentration-matched absorption profiles varied according to the number of arms (Figure 3A), while emission profiles were unaffected (Figure 3B). The three-arm CStar (**7**) failed to achieve an emissive, monomeric absorption profile in water at any concentration tested (Figure S10). On the other hand, the more substituted five- and four-arm CStars (**5** and **6**) were similar, displaying majority monomer absorption profiles across the same concentration range in water (Figure S10). Comparing photophysical properties of the five- and four-arm CStars (**5** and **6**) in water, **5** was superior with higher ε_max_ and Φ_F_ (Figure 3D, Table S1). Since the three-arm CStar (**7**) was primarily aggregated in water, we were not able to thoroughly measure its photophysical properties; however, an excitation spectrum revealed the blue-shifted aggregate peak is indeed nonemissive (Figure S9).

To directly compare relative SWIR brightness in water, concentration-matched emission intensities of the five-, four-, and three-arm CStar variants were measured in capillaries under 974 nm excitation. Consistent with the photophysical measurements, **5** boasted the highest SWIR emission intensity beyond 1100 nm (Figure 3C). Overall, we conclude that five polymer arms are optimal for maximizing both aqueous solubility and brightness, and name the five-arm chromenylium star polymer (**5**) **CStar30**, where the “30” indicates the number of MeOx repeat units for each POx polymer arm. In water, **CStar30** is the brightest primarily SWIR emissive polymethine fluorophore reported to date.^26,53−55^

### Protein Association and Stability under Biological Conditions

**Figure 4:**
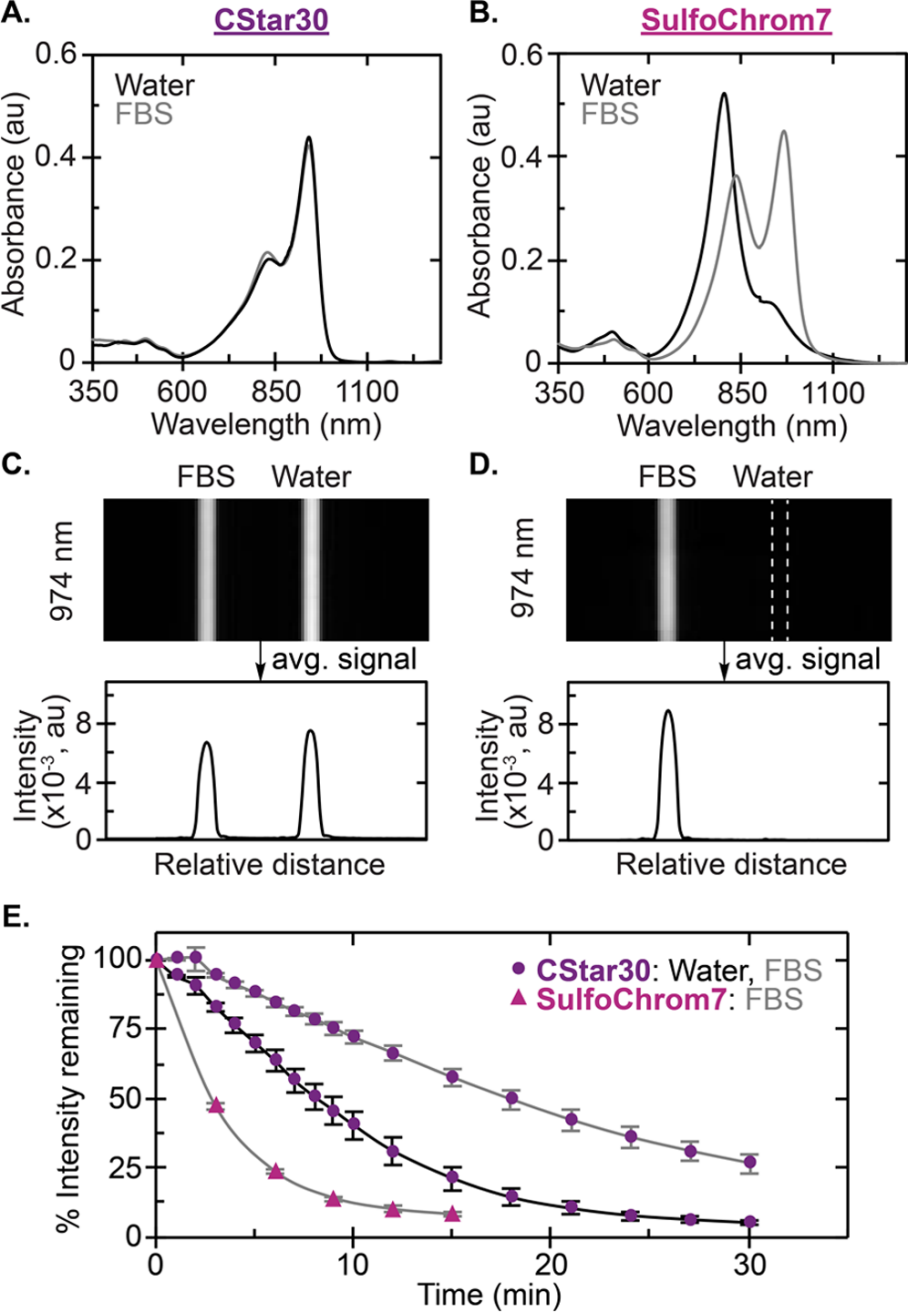
Comparison of **CStar30** and **SulfoChrom7** in water and protein solutions (see Figure 3D, S1 for photophysical properties). (A–B) Absorbance spectra of **CStar30** (A), **SulfoChrom7** (B) in water and FBS (15 μM, 3 mm cuvette). (C) Capillary image and intensity quantification of **CStar30** samples from (A) measured via InGaAs camera (974 nm ex, 100 mW/cm^2^, 5 ms ET, 1100 nm LP). (D) Capillary image and intensity quantification of samples from (B) measured via InGaAs camera (974 nm ex, 100 mW/cm^2^, 2 ms ET, 1100 nm LP). (E) Photobleaching of **CStar30** samples in water and FBS from (A), and **SulfoChrom7**(26) in FBS measured via InGaAs camera (974 nm ex, 100 mW/cm^2^, 5 ms ET CStar30, 30 ms ET **SulfoChrom7**, 1100 nm LP). The photobleaching rate constant for **CStar30** was determined to be 0.09 and 0.043 min^–1^ in water and FBS, respectively (see raw data in Figure S13). For **SulfoChrom7**, the photobleaching rate was reported to be 0.19 min^–1^ in FBS.^26^

We next evaluated the ability of **CStar30** to repel protein capture, as compared to **ICG** and **SulfoChrom7** (Figure S1) as water-soluble small molecule polymethine fluorophore benchmarks. We first examined **CStar30** and **SulfoChrom7** in water and FBS, which contains a high level of albumin protein as well as other proteins and biomolecules (Figure 4). Excitingly, **CStar30** displayed a monomeric absorption profile in water and FBS (Figure 4A, Figure S10), with nearly identical SWIR emission intensity (Figure 4C). Further, the desirable photophysical properties of **CStar30** in water were unaffected in FBS (Figure 3D). Conversely, absorption profiles and SWIR emission intensities varied dramatically from water to FBS for **SulfoChrom7**. Here, nonemissive aggregation dominated in water, abolishing nearly all SWIR emission, while FBS rescued the emissive monomer state (Figure 4B, 4D). As previously characterized, **ICG** displays similar behavior.^27^ Brightness increases in the presence of protein are characteristic of protein association for small molecule fluorophores.^29,30^ Hence, these data suggest that the star polymer architecture has the ability to shield the core fluorophore component of **CStar30** from protein capture.

Next, we probed for any interaction of **CStar30** with proteins via a native PAGE gel. Maintaining proteins in their native state allows for noncovalent interactions to persist, as previously demonstrated with **ICG** binding to albumin.^56^ Using bovine serum albumin (BSA) as a standard, **CStar30** did not show any overlap between fluorescence and protein signal, whereas **SulfoChrom7** fluorescence was readily observed alongside BSA (Figure S11). Gratifyingly, opposite to **SulfoChrom7**, **CStar30** also did not demonstrate any signal overlap when tested with FBS or cell lysate (Figure S11). Taken together, these data support that the star polymer architecture of **CStar30** broadly prevents protein association.

Stability in biologically relevant environments is also notoriously challenging with small molecule dyes. Previous reports have demonstrated that for both **ICG** and **SulfoChrom7**, little fluorophore remains in solution after only a few hours at physiological temperature (37 °C).^26,57,58^ Fortuitously, the stability of **CStar30** was improved in both water and FBS (Figure S12). Furthermore, the stability of **CStar30** was greatly enhanced in the presence of glutathione (GSH) with more than 50% of fluorophore remaining after 20 days in 1 mM GSH, and 55 days in 10 mM GSH (Figure S12). Additionally, photobleaching rates of **CStar30** in both water and FBS solutions were improved ∼10-fold when directly compared to **SulfoChrom7** in FBS^26^ under the same conditions (Figure 4E, S13). **ICG** also has well-documented photostability concerns.^27^ Overall, these results suggest that the star polymer architecture of **CStar30** more effectively shields the fluorophore in aqueous environments, thereby increasing both chemo- and photostability over protein-associated dyes.

### Single Color **CStar30** Biodistribution in Mice

**Figure 5:**
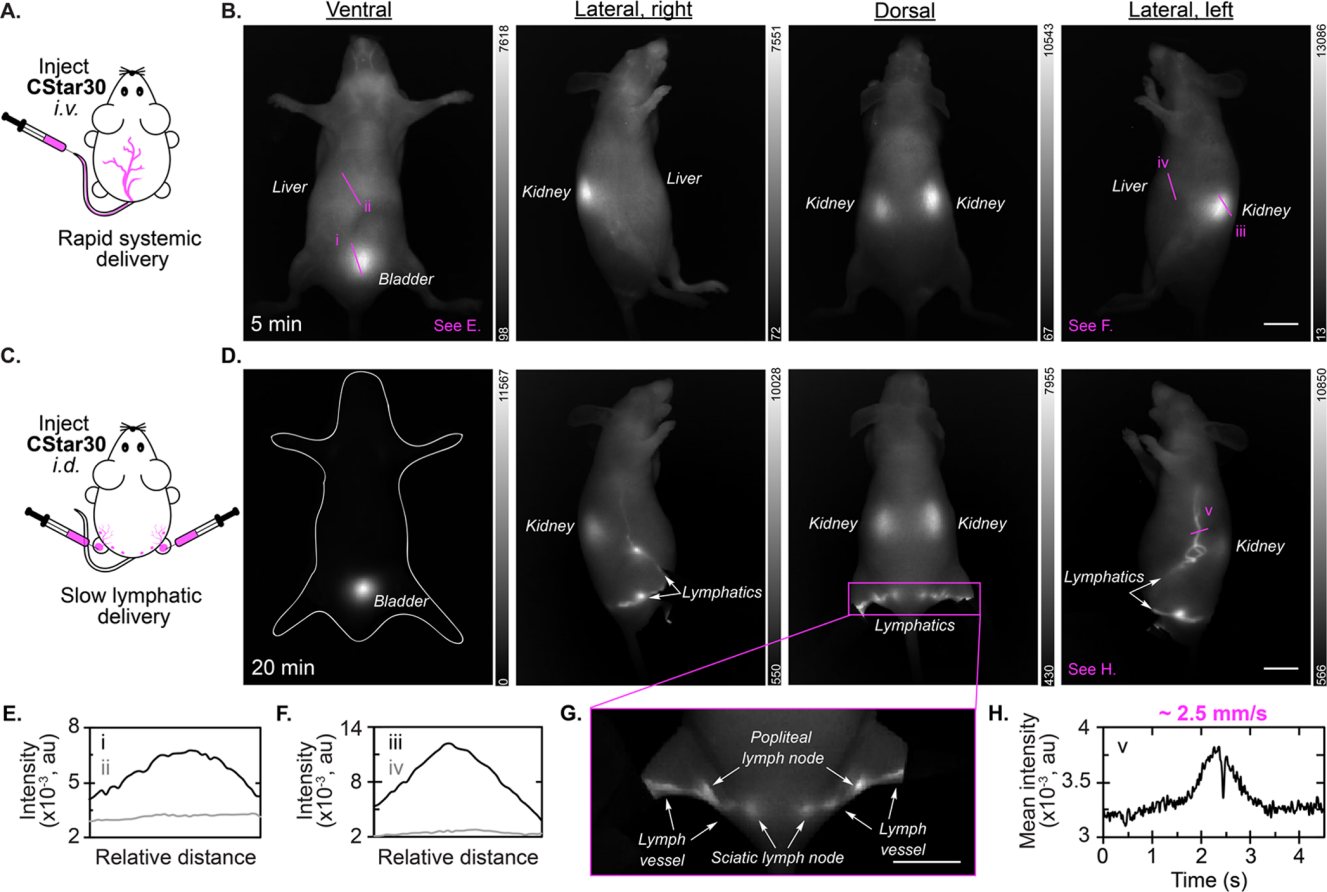
Single color imaging of **CStar30** in mice. (A) Schematic for intravenous (*i.v*.) tail vein injection. (B) *In vivo* images 5 min after *i.v*. injection of **CStar30** dissolved in sterile water (200 μL, 30 nmol) measured via InGaAs camera (974 nm ex, 1100 nm LP, 100 fps). (C) Schematic for intradermal (*i.d*.) hind footpad injections. (D) *In vivo* images 20 min after *i.d*. injection of **CStar30** dissolved in sterile water (50 μL in each hind footpad, 30 nmol total) measured via InGaAs camera (974 nm ex, 1100 nm LP, 50–100 fps). (E–F) Raw intensity profiles across ROI i, ii (E) and iii, iv (F) from (B). (G) Focused region for dorsal lymph visualization 20 min postinjection with hind limbs extended outward, measured via InGaAs camera. (H) *Z*-axis profile across ROI v and corresponding lymphatic flow velocity from (D). See Figure S17 for velocity calculation. See Table S2 for sensitivity related parameters. Scale bars: 10 mm.

With **CStar30** displaying excellent *in vitro* properties, we next prepared for *in vivo* SWIR imaging in mice (Figure 5). Confirming the biocompatibility of **CStar30** via toxicity assessment *in cellulo*, **CStar30** displayed no toxicity to cells at and above the anticipated concentration *in vivo* (Figure S14). This finding is consistent with the known biocompatibility of the core Chrom7 fluorophore and POx polymer arms. We also ensured copper levels were low via ICP-MS, which indicated that at the anticipated dose of 30 nmol, the estimated *in vivo* concentration of Cu is 0.002 ppm from **CStar30**, which is well below toxicity levels (> 1.4 ppm total serum Cu).

Initial *in vivo* experiments involved intravenous (*i.v*.) tail vein injection of **CStar30** in mice (Figure 5A). In contrast to all previously reported experiments with chromenylium fluorophores, we immediately observed kidney localization (Figure 5B, Video S1), followed by increased bladder signal over time (Figure S15, S16). Ultimately, under anesthesia the blood circulation time of **CStar30** is estimated to be on the order of 10–15 min via *i.v*. injection (Figure S17). This biodistribution suggests that **CStar30** is small enough for renal clearance, in line with the predicted hydrodynamic diameter of ∼ 5 nm (estimated from the Flory dimension of a single POx polymer arm).^33^ Additionally, while the vast majority of small molecule polymethine dyes rapidly localize to the liver, primarily as a consequence of protein association, signal in the liver was basal with **CStar30** (Figure 5E, 5F), suggesting stealth behavior *in vivo*. At 3 h postinjection, the overall signal in the animal was substantially reduced, and became nearly undetectable after 1 d (Figure S15, S16). After 2 d, the animal was euthanized. Weak fluorescence intensity in all organs *ex vivo* is consistent with the majority of the probe clearing from the body (Figure S18).

Motivated by this distinct biodistribution and fast clearance, we also introduced **CStar30** intradermally (*i.d*.) via hind footpad injections to visualize the lymphatic system (Figure 5C). Lymph node (LN) mapping and lymphatic drainage rates are important diagnostic tools for cancer staging, but decoupling protein and fluid dynamics remains a challenge due to protein association of small molecule dyes.^32,59^ After *i.d.* injection, LNs were strongly illuminated by **CStar30**, which, combined with a high-resolution SWIR detection window, allowed for facile noninvasive LN identification (Figure 5D, 5G). LNs are difficult to identify with white light even *ex vivo* due to their small size and indistinguishable appearance from other tissues (Figure S19). The high-resolution SWIR region allowed for facile image-guided resection of LNs (Video S2). *In vivo*, we were able to readily monitor the dye pulsing through lymph vessels in real time and calculated a lymphatic drainage velocity of ∼ 2.5 mm/min under anesthesia (Figure 5H, Figure S20, Video S3). While previous reports of lymphatic visualization via fluorescence imaging vary drastically with respect to LN identification and drainage velocities,^60−62^ we hypothesize that minimizing nonspecific interactions of proteins and other biomolecules with **CStar30** provides a more accurate representation of these dynamics.

As **CStar30** drained from LNs and was reabsorbed to the bloodstream, kidney and bladder localization increased proportionally over time (Figure S21, S22). Owed to the slow-absorbing nature of an *i.d*. injection mode, renal clearance was mildly slower compared to an *i.v*. injection. Still, after 2 d postinjection, the majority of **CStar30** was absorbed from the footpads, and *ex vivo* organ analysis confirms efficient clearance overall (Figure S23).

### Two Color Imaging in Mice: Stealthy **CStar30** vs. Protein-Associated **ICG**

**Figure 6:**
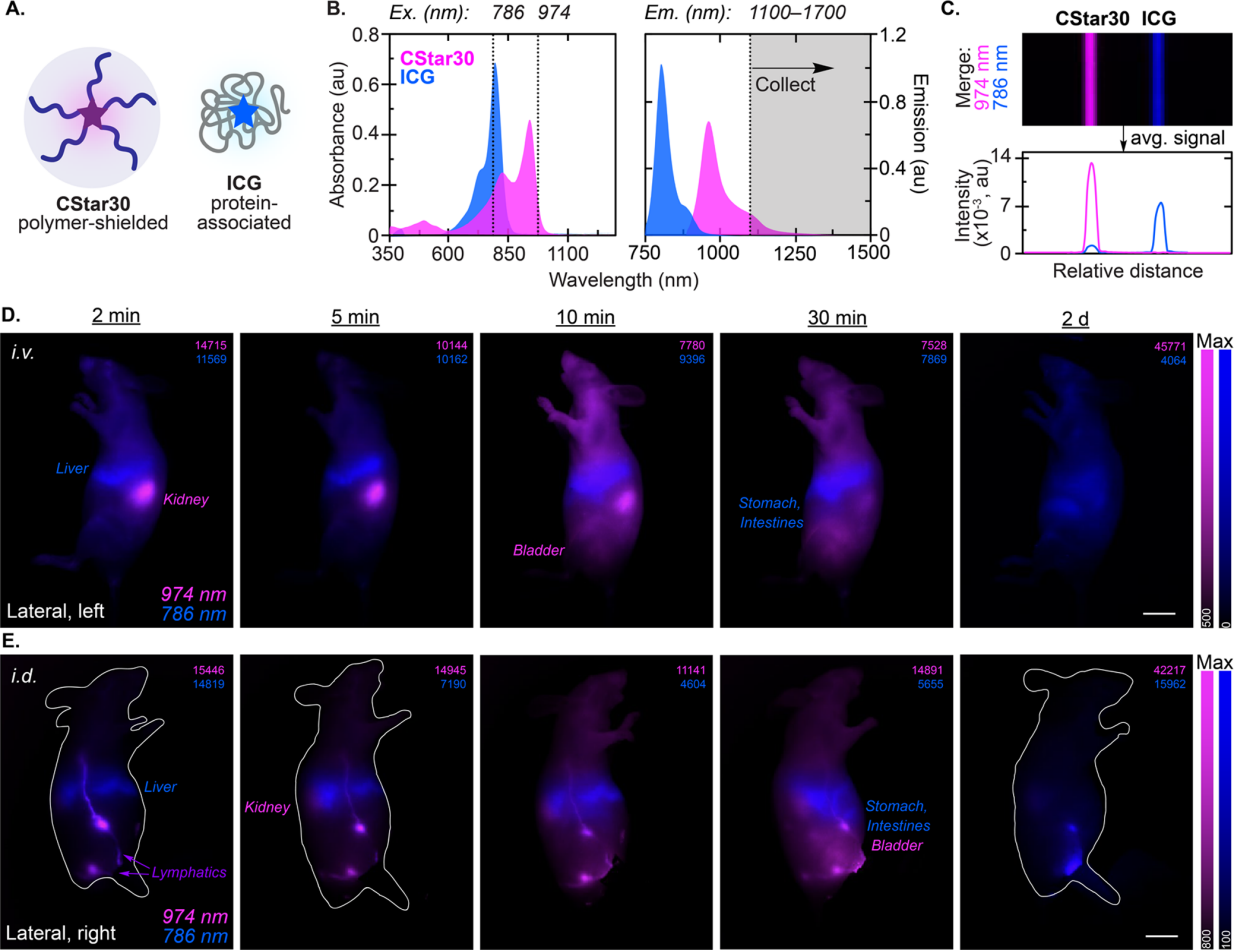
Two-color excitation-multiplexed imaging of **CStar30** and **ICG** in mice. (A) Schematic of **CStar30** shielded by POx polymer arms versus **ICG** associated with protein (see Figure S1 and S3 for structures). (B) Absorbance and emission spectra of **CStar30** (15 μM) in water and **ICG** (100 μM) in FBS. Measurements were taken with a 3 mm cuvette. **CStar30** emission spectra: 860 nm ex, 885–1500 nm em. **ICG** emission spectra: 720 nm ex, 740–1400 nm em. SWIR excitation-multiplexed imaging parameters are schematized: excitation at either 974 nm (**CStar30**) or 786 nm (**ICG**), and a single SWIR emission window > 1100 nm, defined by an 1100 nm LP filter. (C) Capillary images of samples from (B) measured via InGaAs camera. Merged channels 974 nm ex (magenta, 160 mW/cm^2^, 5 ms ET) and 786 nm ex (blue, 100 mW/cm^2^, 1 ms ET) and intensity quantification. See Figure S22 for single channel images. (D) Two-color timecourse of 974 nm (magenta) and 786 nm (blue) excitation channels after *i.v.* coinjection of **CStar30** and **ICG** in sterile water (100 μL each dye, 30 nmol **CStar30** and 200 nmol **ICG**) measured via InGaAs camera (786 and 974 nm ex, 1100 nm LP, 15–100 fps). (E) Two-color timecourse of 974 (magenta) and 786 nm (blue) excitation channels after *i.d.* coinjection of **CStar30** and **ICG** in sterile water (25 μL each dye in each hind footpad, overall 30 nmol **CStar30** and 200 nmol **ICG**) measured via InGaAs camera (786 and 974 nm ex, 1100 nm LP, 17–100 fps). See Supporting Information for all corresponding single channel images and Table S3 for sensitivity related parameters. Note that organs are labeled at first appearance in the timecourse, **CStar30** and **ICG** fluorescence are colored magenta and blue, respectively, with colocalization of the fluorophores colored purple. Max intensity: upper right of each image. Scale bars: 10 mm.

To further explore the difference in *in vivo* dynamics observed between a protein-associated and protein-inert fluorophore, we performed excitation-multiplexed two color imaging with **ICG** as a protein-associated dye, and **CStar30** as a protein-inert dye (Figure 6A). **ICG** was selected as it is spectrally separated from **CStar30**, allowing for alternating excitation of each dye at either 786 and 974 nm, respectively, with a single SWIR emission window (Figure 6B, Figure S24). This approach allows for visualization of both fluorophores at video-rate imaging speeds. Although **ICG** is a primarily NIR emitting fluorophore, a minute emission tail can still be visualized in the SWIR, albeit with higher injection concentrations.^3,27,28^ We determined that a concentration ratio of 15 to 100 μM (**CStar30**:**ICG**) was optimal for eliminating crosstalk between excitation channels (Figure 6C, Figure S25), setting the stage for two-color imaging *in vivo*.

Co-injecting **CStar30** and **ICG** via *i.v.* or *i.d.* injections, we directly compared biodistribution dynamics and clearance timelines of each fluorophore in the same animal. This approach reduces biological variability and increases the significance of *in vivo* imaging observations. First, with an *i.v.* tail vein injection, we observed **ICG** traverse through the vasculature and accumulate in the liver within 2 min postinjection, consistent with protein association (Figure 6D, blue). On the other hand, polymer-shielded **CStar30** avoided protein capture and traveled to the kidneys in the same time frame (Figure 6D, magenta). The animal was imaged in intervals up to 30 min before coming out of anesthesia and excreting the fluorophores. During this time, we observed progressively increased signal from **ICG** in the liver (Figure 6D, blue). Meanwhile, signal from **CStar30** in the kidneys began decreasing around 10 min postinjection, and by the 30 min time point, bladder accumulation was most prominent (Figure 6D, magenta). At 3 h postinjection, strong signal in the liver, stomach and intestines was observed for **ICG**, but negligible signal was detected from **CStar30** in the kidneys, along with relatively low signal remaining in the bladder (Figure S26, S27). After 1 d, there was no detectable signal for **CStar30** (Figure S26, S27). However, **ICG** was still readily visualized in tissues associated with the reticuloendothelial system (RES), such as the liver, 2 d postinjection (Figure 6D, blue). Such *in vivo* retention is characteristic for **ICG** and other small molecule protein-associated dyes. Again, *ex vivo* analysis of excised organs is consistent with the *in vivo* biodistribution and clearance observations (Figure S28).

Finally, we performed the same coinjection experiment with **CStar30** and **ICG** via *i.d.* hind footpad injections to assess how the distinct biodistribution and clearance rates extend to visualizing the lymphatic system. Gratifyingly, similar trends were observed. Immediately following injection, both dyes were strongly localized to the same LNs (Figure 6E, purple). ICG was also trafficked to the liver in the same time frame (Figure 6E, blue), whereas **CStar30** exhibited slower drainage from LNs, evident by low signal in the kidneys and bladder up to 10 min postinjection (Figure 6E, magenta). In fact, strong LN signal was present for **CStar30** for up to 1 h postinjection (Figure S29, S30). Following the same trend as the *i.v.* coinjection, **ICG** signal in RES-associated tissues was still readily visualized 2 d postinjection (Figure 6E, blue), while **CStar30** was undetectable. This *in vivo* observation was further validated via *ex vivo* organ analysis (Figure S31). Ultimately, this direct comparison reveals how protein association with **ICG** may confound LN mapping and lymphatic drainage monitoring over time.

We conclude that **CStar30** and **ICG** can be employed in tandem for SWIR imaging as complementary fluorophores to visualize both fluid and protein dynamics in the same animal. Additionally, single channel images suggest that crosstalk was negligible between the two dyes, allowing images to be analyzed without need for linear unmixing corrections (see Supporting Information). The orthogonal biodistributions, independent of the mode of injection, demonstrated by **CStar30** and **ICG** highlight how a simple chemical modification can instill distinct changes in *in vivo* localization and clearance rates.

## Conclusion

Surveying the breadth of polymethine fluorophores available for fluorescence imaging *in vivo*, it is clear that SWIR brightness, aqueous solubility, and the propensity for biomolecule interaction fluctuate significantly. In this work, we merged stealthy, hydrophilic linear POx polymers with SWIR emissive chromenylium heptamethine fluorophores via click chemistry to arrive at chromenylium star polymers (CStars). Evaluating the number of polymer arms required for maintaining the bright nature of the SWIR emissive fluorophore core in water, we discovered five arms to be optimal. This modification also has the benefit of substantially enhancing both chemo- and photostability in aqueous environments. Moving forward with **5** (**CStar30**), we demonstrated that the star polymer architecture repels protein interaction *in vitro*, evident by the negligible differences in absorption profiles as well as SWIR brightness in water versus protein solutions.

As the first in its class, we applied **CStar30** for *in vivo* SWIR imaging in mice via both *i.v.* and *i.d.* injections. Here, we were delighted to observe that the dye clears readily through the renal system, independent of the mode of injection, as SWIR chromenylium dyes have been plagued by liver accumulation. For the *i.d.* injection, we were also able to easily monitor LN drainage in real time — an important biological marker of disease states. Moreover, in excitation-multiplexed imaging experiments with **CStar30** and **ICG**, we showcased the differences in biodistribution and *in vivo* dynamics imbued by the star polymer architecture versus a protein-associated small molecule dye.

Having finally surmounted the barrier of biomolecule interactions with a water-soluble, SWIR emissive polymethine fluorophore, we believe the CStar architecture has the potential to greatly improve noninvasive clinically relevant endeavors such as monitoring renal function and lymphatic drainage *in vivo*. Furthermore, with the synthetic accessibility of attaching POx polymer arms, we envision that this strategy will be amenable to a variety of fluorophore scaffolds.

## Data Availability

*In vivo* imaging data files for this work are available at BioImage Archive, accession number: S-BIAD1368. These data can be obtained free of charge via https://www.ebi.ac.uk/biostudies/bioimages/studies/S-BIAD1368. Custom software used in this manuscript can be found at GitLab. The software can be accessed free of charge via https://gitlab.com/brunslab/ccda.

## Supporting Information Available

Figures S1–S31, Schemes S1–S5, Tables S1–S3, experimental procedures, and synthesis and characterization for all new compounds (PDF)
Video S1: Single Channel Injection of **CStar30** (*i.v.*) in Mice (AVI)
Video S2: Single Channel *Ex Vivo* Image-Guided Lymph Node Resection (AVI)
Video S3: Single Channel Lymphatic Vessel Velocity of **CStar30** in Mice (AVI)

## Supplementary Material

oc4c01570_si_001.pdf

oc4c01570_si_002.avi

oc4c01570_si_003.avi

oc4c01570_si_004.avi

## Abbreviations

abs: absorbance
BSA: bovine serum albumin
CStar: chromenylium star
CuAAC: copper-catalyzed azide–alkyne cycloaddition
d: days
em: emission
ET: exposure time
ex: excitation
FBS: fetal bovine serum
fps: frames per second
GPC: gel permeation chromatography
GSH: l-glutathione
*i.d*.: intradermal
*i.v*.: intravenous
ICG: indocyanine green
LN: lymph node
LP: long pass filter
MALDI: matrix-assisted laser desorption ionization mass spectrometry
max: maximum
MeOX: 2-methyl-2-oxazoline
min: minutes
ms: milliseconds
NIR: near-infrared
PEG: poly(ethylene glycol)
POx: poly(2-methyl-2-oxazoline)
RES: reticuloendothelial system
ROI: region of interest
SWIR: shortwave infrared

## References

[ref1] Lavis L. D. Chemistry is Dead. Long Live Chemistry!. Biochemistry. 2017, 56, 5165–5170. 10.1021/acs.biochem.7b00529. 28704030

[ref2] Grimm J. B.; Lavis L. D. Caveat Fluorophore: An Insider’s Guide to Small-Molecule Fluorescent Labels.. Nat. Methods. 2022, 19, 149–158. 10.1038/s41592-021-01338-6. 34949811

[ref3] van Manen L.; Handgraaf H. J. M.; Diana M.; Dijkstra J.; Ishizawa T.; Vahrmeijer A. L.; Mieog J. S. D. A Practical Guide for the Use of Indocyanine Green and Methylene Blue in Fluorescence-Guided Abdominal Surgery.. J. Surg. Oncol. 2018, 118, 283–300. 10.1002/jso.25105. 29938401 PMC6175214

[ref4] Hu Z.; Fang C.; Li B.; Zhang Z.; Cao C.; Cai M.; Su S.; Sun X.; Shi X.; Li C.; Zhou T.; Zhang Y.; Chi C.; He P.; Xia X.; Chen Y.; Gambhir S. S.; Cheng Z.; Tian J. First-In-Human Liver-Tumour Surgery Guided by Multispectral Fluorescence Imaging in the Visible and Near-Infrared-I/II Windows.. Nat. Biomed. Eng. 2020, 4, 259–271. 10.1038/s41551-019-0494-0. 31873212

[ref5] Marshall M. V.; Rasmussen J. C.; Tan I.; Aldrich M. B.; Adams K. E.; Wang X.; Fife C. E.; Maus E. A.; Smith L. A.; Sevick-Muraca E. M. Near-Infrared Fluorescence Imaging in Humans with Indocyanine Green: A Review and Update.. Open Surg. Oncol. J. 2010, 2 (2), 12–25. 10.2174/1876504101002010012. 22924087 PMC3424734

[ref6] Chen Y.; Wang S.; Zhang F. Near-Infrared Luminescence High-contrast In Vivo Biomedical Imaging.. Nat. Rev. Bioeng. 2023, 1, 60–78. 10.1038/s44222-022-00002-8.

[ref7] Markuszewski M.; Buszewska-Forajta M.; Artymowicz M.; Polom W.; Roslan M.; Markuszewski M. Binding Indocyanine Green to Human Serum Albumin Potentially Enhances the Detection of Sentinel Lymph Nodes. An Initial Step for Facilitating the Detection of First-Station Nodes in Penile and Other Urological Cancers. Arch Med Sci. 2022, 18 (3), 719–725. 10.5114/aoms/113237. 35591825 PMC9102538

[ref8] Mahalingam S. M.; Kularatne S. M.; Myers C. H.; Gagare P.; Norshi M.; Liu X.; Singhal S.; Low P. S. Evaluation of Novel Tumor-Targeted Near-Infrared Probe for Fluorescence-Guided Surgery of Cancer.. J. Med. Chem. 2018, 61, 9637–9646. 10.1021/acs.jmedchem.8b01115. 30296376

[ref9] Carr J. A.; Franke D.; Caram J. R.; Perkinson C. F.; Saif M.; Askoxylakis V.; Datta M.; Fukumura D.; Jain R. K.; Bawendi M. G.; Bruns O. T. Shortwave Infrared Fluorescence Imaging with the Clinically Approved Near-Infrared Dye Indocyanine Green.. Proc. Natl. Acad. Sci. U.S.A. 2018, 115 (17), 4465–4470. 10.1073/pnas.1718917115. 29626132 PMC5924901

[ref10] Diao S.; Hong G.; Antaris A. L.; Blackburn J. L.; Cheng K.; Cheng Z.; Dai H. Biological Imaging Without Autofluorescence in the Second Near-Infrared Region.. Nano Res. 2015, 8 (9), 3027–3034. 10.1007/s12274-015-0808-9.

[ref11] Carr J. A.; Aellen M.; Franke D.; So P. T. C.; Bruns O. T.; Bawendi M. G. Absorption by Water Increases Fluorescence Image Contrast of Biological Tissue in the Shortwave Infrared.. Proc. Natl. Acad. Sci. U.S.A. 2018, 115 (37), 9080–9085. 10.1073/pnas.1803210115. 30150372 PMC6140498

[ref12] Jiang G.; Liu H.; Liu H.; Ke G.; Ren T.-B.; Xiong B.; Zhang X.-B.; Yuan L. Chemical Approaches to Optimize the Properties of Organic Fluorophores for Imaging and Sensing.. Angew. Chem., Int. Ed. 2024, 63, e202315217 10.1002/anie.202315217. 38081782

[ref13] Wen P.; Ke W.; Dirisala A.; Toh K.; Tanaka M.; Li J. Stealth and Pseudo-Stealth Nanocarriers.. Adv. Drug. Delivery Rev. 2023, 198, 114895 10.1016/j.addr.2023.114895. 37211278

[ref14] Luciano M. P.; Crooke S. N.; Nourian S.; Dingle I.; Nani R. R.; Kline G.; Patel N. L.; Robinson C. M.; Difilippantonio S.; Kalen J. D.; Finn M. G.; Schnermann M. J. A Nonaggregating Heptamethine Cyanine for Building Brighter Labeled Biomolecules.. ACS Chem. Biol. 2019, 14, 934–940. 10.1021/acschembio.9b00122. 31030512 PMC6528163

[ref15] Cosco E. D.; Arus B. A.; Spearman A. L.; Atallah T. L.; Lim I.; Leland O. S.; Caram J. R.; Bischof T. S.; Bruns O. T.; Sletten E. M. Bright Chromenylium Polymethine Dyes Enable Fast, Four-Color In Vivo Imaging with Shortwave Infrared Detection.. J. Am. Chem. Soc. 2021, 143 (18), 6836–6846. 10.1021/jacs.0c11599. 33939921 PMC8327756

[ref16] Lavis L. D.; Raines R. T. Bright Building Blocks for Chemical Biology. ACS Chem. Biol. 2014, 9, 855–866. 10.1021/cb500078u. 24579725 PMC4006396

[ref17] Thimsen E.; Sadtler B.; Berezin M. Y. Shortwave-Infrared (SWIR) Emitters for Biological Imaging: A Review of Challenges and Opportunities.. Nanophotonics. 2017, 6 (5), 1043–1054. 10.1515/nanoph-2017-0039.

[ref18] Feng X.; Wei L.; Liu Y.; Chen X.; Tian R. Orchestrated Strategies for Developing Fluorophores for NIR-II Imaging.. Adv. Healthcare Mater. 2023, 12, 2300537 10.1002/adhm.202300537. 37161650

[ref19] Terenziani F.; Przhonska O. V.; Webster S.; Padilha L. A.; Slominsky Y. L.; Davydenko I. G.; Gerasov A. O.; Kovtun Y. P.; Shandura M. P.; Kachkovski A. D.; Hagan D. J.; Van Stryland E. W.; Painelli A. Essential-State Model for Polymethine Dyes: Symmetry Breaking and Optical Spectra.. J. Phys. Chem. Lett. 2010, 1, 1800–1804. 10.1021/jz100430x.

[ref20] Maillard J.; Klehs K.; Rumble C.; Vauthey E.; Heilemann M.; Fürstenberg A. Universal Quenching of Common Fluorescent Probes by Water and Alcohols.. Chem. Sci. 2021, 12, 1352–1362. 10.1039/D0SC05431C. PMC8179231 34163898

[ref21] Friedman H. C.; Cosco E. D.; Atallah T. L.; Jia S.; Sletten E. M.; Caram J. R. Establishing Design Principles for Emissive Organic SWIR Chromophores from Energy Gap Laws.. Chem. 2021, 7, 3359–3376. 10.1016/j.chempr.2021.09.001. 34901520 PMC8664240

[ref22] Lovell T. C.; Branchaud B. P.; Jasti R. Organic Chemist’s Guide to Fluorophores - Understanding Common and Newer Non-Planar Fluorescent Molecules for Biological Applications.. Eur. J. Org. Chem. 2024, 27, e202301196 10.1002/ejoc.202301196.

[ref23] Cosco E. D.; Caram J. R.; Bruns O. T.; Franke D.; Day R. A.; Farr E. P.; Bawendi M. G.; Sletten E. M. Flavylium Polymethine Fluorophores for Near- and Shortwave Infrared Imaging.. Angew. Chem., Int. Ed. 2017, 56 (42), 13126–13129. 10.1002/anie.201706974. 28806473

[ref24] Tao Z.; Hong G.; Shinji C.; Chen C.; Diao S.; Antaris A. L.; Zhang B.; Zou Y.; Dai H. Biological Imaging Using Nanoparticles of Small Organic Molecules with Fluorescence Emission at Wavelengths Longer than 1000 nm.. Angew. Chem., Int. Ed. 2013, 52, 13002–13006. 10.1002/anie.201307346. 24174264

[ref25] Hua S.; de Matos M. B.; Metselaar J. M.; Storm G. Current Trends and Challenges in the Clinical Translation of Nanoparticulate Nanomedicines: Pathways for Translational Development and Commercialization.. Front. Pharmacol. 2018, 9, 790 10.3389/fphar.2018.00790. 30065653 PMC6056679

[ref26] Jia S.; Lin E. Y.; Mobley E. B.; Lim I.; Guo L.; Kallepu S.; Low P. S.; Sletten E. M. Water-Soluble Chromenylium Dyes for Shortwave Infrared Imaging in Mice.. Chem. 2023, 9 (12), 3648–3665. 10.1016/j.chempr.2023.08.021. 38283614 PMC10817055

[ref27] Cosco E. D.; Lim I.; Sletten E. M. Photophysical Properties of Indocyanine Green in the Shortwave Infrared Region.. ChemPhotoChem. 2021, 5, 727–734. 10.1002/cptc.202100045. 34504949 PMC8423351

[ref28] Gao S.; Yu Y.; Wang Z.; Wu Y.; Qiu X.; Jian C.; Yu A. NIR-II Fluorescence Imaging Using Indocyanine Green Provides Early Prediction of Skin Avulsion-Injury in a Porcine Model.. Clin. Cosmet. Investig. Dermatol. 2022, 15, 447–454. 10.2147/CCID.S357989. PMC8923835 35308638

[ref29] Costa S. M. B.; Tatikolov A. S. Complexation of Polymethine Dyes with Human Serum Albumin: A spectroscopic Study.. Biophys. Chem. 2004, 107, 33–49. 10.1016/S0301-4622(03)00218-7. 14871599

[ref30] Davies-Venn C. A.; Angermiller B.; Wilganowski N.; Ghosh P.; Harvey B. R.; Wu G.; Kwon S.; Aldrich M. B.; Sevick-Muraca E. M. Albumin-Binding Domain Conjugate for Near-Infrared Fluorescence Lymphatic Imaging.. Mol. Imaging Biol. 2012, 14, 301–314. 10.1007/s11307-011-0499-x. 21688052 PMC3346932

[ref31] Tian R.; Zeng Q.; Zhu S.; Lau J.; Chandra S.; Ertsey R.; Hettie K. S.; Teraphongphom T.; Hu Z.; Niu G.; Kiesewetter D. O.; Sun H.; Zhang X.; Antaris A. L.; Brooks B. R.; Chen X. Albumin-Chaperoned Cyanine Dye Yields Superbright NIR-II Fluorophore with Enhanced Pharamacokinetics.. Sci. Adv. 2019, 5, eaaw0672 10.1126/sciadv.aaw0672. 31548981 PMC6744268

[ref32] Abdallah M.; Müllertz O. O.; Styles I. K.; Mörsdorf A.; Quinn J. F.; Whittaker M. R.; Trevaskis N. L. Lymphatic Targeting by Albumin-Hitchhiking: Applications and Optimisation.. JCR. 2020, 327, 117–128. 10.1016/j.jconrel.2020.07.046. 32771478

[ref33] Bludau H.; Czapar A. E.; Pitek A. S.; Shukla S.; Jordan R.; Steinmetz N. F. POxylation as an Alternative Stealth Coating for Biomedical Applications.. Eur. Polym. J. 2017, 88, 679–688. 10.1016/j.eurpolymj.2016.10.041. 28713172 PMC5510027

[ref34] Knop K.; Hoogenboom R.; Fischer D.; Schubert U. S. Poly(ethylene glycol) in Drug Discovery: Pros and Cons as Well as Potential Alternatives.. Angew. Chem., Int. Ed. 2010, 49 (36), 6288–6308. 10.1002/anie.200902672. 20648499

[ref35] D’souza A. A.; Shegokar R. Polyethylene glycol (PEG): A Versatile Polymer for Pharmacuetical Applications.. Expert Opin. Drug Delivery. 2016, 13 (9), 1257–1275. 10.1080/17425247.2016.1182485. 27116988

[ref36] Wyffels L.; Verbrugghen T.; Monnery B. D.; Glassner M.; Stroobants S.; Hoogenboom R.; Staelens S. μPET Imaging of the Pharmacokinetic Behavior of Medium and High Molar Mass 89Zr-Labeled Poly(2-ethyl-2-oxazoline) in Comparison to Poly(ethylene glycol).. J Control Release 2016, 235, 63–71. 10.1016/j.jconrel.2016.05.048. 27235979

[ref37] Moreadith R. W.; Viegas T. X.; Bentley M. D.; Harris J. M.; Fang Z.; Yoon K.; Dizman B.; Weimer R.; Rae B. P.; Li Z.; Rader C.; Standaert D.; Olanow W. Clinical Development of a Poly(2-oxazoline) (POZ) Polymer Therapeutic for the Treatment of Parkinson’s Disease - Proof of Concept of POZ as a Versatile Polymer Platform for Drug Development in Multiple Therapeutic Indications.. Eur. Polym. J. 2017, 88, 524–552. 10.1016/j.eurpolymj.2016.09.052.

[ref38] Viegas T. X.; Bentley M. D.; Harris J. M.; Fang Z.; Yoon K.; Dizman B.; Weimer R.; Mero A.; Pasut G.; Veronese F. M. Polyoxazoline: Chemistry, Properties, and Applications in Drug Delivery.. Bioconjugate Chem. 2011, 22 (5), 976–986. 10.1021/bc200049d. 21452890

[ref39] Verbraeken B.; Monnery B. D.; Lava K.; Hoogenboom R. The Chemistry of Poly(2-oxazoline)s.. Eur. Polym. J. 2017, 88, 451–469. 10.1016/j.eurpolymj.2016.11.016.

[ref40] Proulx S. T.; Luciani P.; Christiansen A.; Karaman S.; Blum K. S.; Rinderknecht M.; Leroux J.; Detmar M. Use of a PEG-Conjugated Bright Near-Infrared Dye for Functional Imaging of Rerouting of Tumor Lymphatic Drainage After Sentinel Lymph Node Metastasis.. Biomaterials. 2013, 34, 5128–5137. 10.1016/j.biomaterials.2013.03.034. 23566803 PMC3646951

[ref41] Bagby T. R.; Duan S.; Cai S.; Yang Q.; Thati S.; Berkland C.; Aires D. J.; Forrest M. L. Lymphatic Trafficking Kinetics and Near-Infrared Imaging Using Star Polymer Architectures with Controlled Anionic Character.. Eur. J. Pharma. Sci. 2012, 47, 287–294. 10.1016/j.ejps.2012.04.016. PMC4301975 22546180

[ref42] Hoffmann S.; Vystrčilová L.; Ulbrich K.; Etrych T.; Caysa H.; Mueller T.; Mäder K. Dual Fluorescent HPMA Copolymers for Passive Tumor Targeting with pH-sensitive Drug Release: Synthesis and Characterization of Distribution and Tumor Accumulation in Mice by Noninvasive Multispectral Optical Imaging.. Biomacromolecules. 2012, 13, 652–663. 10.1021/bm2015027. 22263698

[ref43] Ren J. M.; McKenzie T. G.; Fu Q.; Wong E. H. H.; Xu J.; An Z.; Shanmugam S.; Davis T. P.; Boyer C.; Qiao G. G. Star Polymers.. Chem. Rev. 2016, 116, 6743–6836. 10.1021/acs.chemrev.6b00008. 27299693

[ref44] Li D.; Schreiber C. L.; Smith B. D. Sterically Shielded Heptamethine Cyanine Dyes for Bioconjugation and High Performance Near-Infrared Fluorescence Imaging.. Angew. Chem., Int. Ed. 2020, 59, 12154–12161. 10.1002/anie.202004449. PMC7473488 32324959

[ref45] Yang J.; Wang K.; Zheng Y.; Piao Y.; Wang J.; Tang J.; Shen Y.; Zhou Z. Molecularly Precise, Bright, Photostable, and Biocompatible Cyanine Nanodots as Alternatives to Quantum Dots for Biomedical Applications.. Angew. Chem., Int. Ed. 2022, 61, e202202128 10.1002/anie.202202128. 35652391

[ref46] Wei R.; Dong Y.; Wang X.; Li J.; Lei Z.; Hu Z.; Chen J.; Sun H.; Chen H.; Luo X.; Qian X.; Yang Y. Rigid and Photostable Shortwave Infrared Dye Absorbing/Emitting beyond 1200 nm for High-Contrast Multiplexed Imaging.. J. Am. Chem. Soc. 2023, 145 (22), 12013–12022. 10.1021/jacs.3c00594. 37216464

[ref47] Yang S. K.; Shi X.; Park S.; Ha T.; Zimmerman S. C. A Dendtritic Single-Molecule Fluorescent Probe that is Monovalent, Photostable and Minimally Blinking.. Nat. Chem. 2013, 5, 692–697. 10.1038/nchem.1706. 23881501 PMC4104187

[ref48] Antaris A. L.; Chen H.; Cheng K.; Sun Y.; Hong G.; Qu C.; Diao S.; Deng Z.; Hu X.; Zhang B.; Zhang X.; Yaghi O. K.; Alamparambil Z. R.; Hong X.; Cheng Z.; Dai H. A Small-Molecule Dye for NIR-II Imaging.. Nat. Mater. 2016, 15, 235–242. 10.1038/nmat4476. 26595119

[ref49] Estabrook D. A.; Ennis A. F.; Day R. A.; Sletten E. M. Controlling Nanoemulsion Surface Chemistry with Poly(2-oxazoline) Amphiphiles.. Chem. Sci. 2019, 10, 3994–4003. 10.1039/C8SC05735D. 31015940 PMC6457192

[ref50] Ivanova R.; Komenda T.; Bonné T. B.; Lüdtke K.; Mortensen K.; Pranzas P. K.; Jordan R.; Papadakis C. M. Micellar Structures of Hydrophilic/Lipophilic and Hydrophilic/Fluorophilic Poly(2-oxazoline) Diblock Copolymers in Water.. Macromol. Chem. Phys. 2008, 209, 2248–2258. 10.1002/macp.200800232.

[ref51] Hoogenboom R. Click Chemistry in Polymer Science.. Chem. 2023, 9 (9), 2416–2424. 10.1016/j.chempr.2023.08.010.

[ref52] Li Y.; Zhang B.; Hoskins J. N.; Grayson S. M. Synthesis, Purification, and Characterization of ‘‘Perfect’’ Star Polymers via ‘‘Click’’ Coupling.. J. Polym. Sci. A Polym. Chem. 2012, 50, 1086–1101. 10.1002/pola.25864.

[ref53] Bandi V. G.; Luciano M. P.; Saccomano M.; Patel N. L.; Bischof T. S.; Lingg J. G. P.; Tsrunchev P. T.; Nix M. N.; Ruehle B.; Sanders C.; Riffle L.; Robinson C. M.; Difilippantonio S.; Kalen J. D.; Resch-Genger U.; Ivanic J.; Bruns O. T.; Schnermann M. J. Targeting Multicolor In Vivo Imaging Over 1,000 nm Enabled by Nonamethine Cyanines.. Nat. Methods. 2022, 19, 353–358. 10.1038/s41592-022-01394-6. 35228725

[ref54] Swamy M. M. M.; Murai Y.; Monde K.; Tsuboi S.; Jin T. Shortwave-Infrared Fluorescent Molecular Imaging Probes Based on -Conjugation Extended Indocyanine Green.. Bioconjugate Chem. 2021, 32, 1541–1547. 10.1021/acs.bioconjchem.1c00253. 34309379

[ref55] Li B.; Zhao M.; Feng L.; Dou C.; Ding S.; Zhou G.; Lu L.; Zhang H.; Chen F.; Li X.; Li G.; Zhao S.; Jiang C.; Wang Y.; Zhao D.; Cheng Y.; Zhang F. Organic NIR-II Molecule with Long Blood Half-Life for In Vivo Dynamic Vasculature Imaging.. Nat. Commun. 2020, 11, 3102 10.1038/s41467-020-16924-z. 32555157 PMC7303218

[ref56] Jang H. J.; Song M. G.; Park C. R.; Youn H.; Lee Y.; Cheon G. J.; Kang K. W. Imaging of Indocyanine Green-Human Serum Albumin (ICG-HAS) Complex in Secreted Protein Acidic and Rich in Cysteine (SPARC)-Expressing Glioblastoma.. Int. J. Mol. Sci. 2023, 24, 850 10.3390/ijms24010850. 36614294 PMC9821702

[ref57] Gathje J.; Steuer R. R.; Nicholes K. R. Stability Studies on Indocyanine Green Dye.. J. Appl. Physiol. 1970, 29 (2), 181–185. 10.1152/jappl.1970.29.2.181. 4913806

[ref58] Mindt S.; Karampinis I.; John M.; Neumaier M.; Nowak K. Stability and Degradation of Indocyanine Green in Plasma, Aqueous Solution and Whole Blood.. Photochem. Photobiol. Sci. 2018, 17, 1189–1196. 10.1039/c8pp00064f. 30028469

[ref59] Qi S.; Wang X.; Chang K.; Shen W.; Yu G.; Du J. The Bright Future of Nanotechnology in Lymphatic System Imaging and Image-Guided Surgery.. J. Nanobiotechnol. 2022, 20, 24 10.1186/s12951-021-01232-5. PMC8740484 34991595

[ref60] Proulx S. T.; Ma Q.; Andina D.; Leroux J.; Detmar M. Quantitative Measurement of Lymphatic Function in Mice by Noninvasive Near-Infrared Imaging of a Peripheral Vein.. JCI Insight. 2017, 2 (1), e90861 10.1172/jci.insight.90861. 28097238 PMC5214224

[ref61] Kwon K.; Sevick-Muraca E. M. Noninvasive Quantitative Imaging of Lymph Function in Mice.. Lymphatic Research and Biology. 2007, 5 (4), 219–231. 10.1089/lrb.2007.1013. 18370912

[ref62] Bouta E. M.; Wood R. W.; Brown E. B.; Rahimi H.; Ritchlin C. T.; Schwarz E. M. In Vivo Quantification of Lymph Viscosity and Pressure in Lymphatic Vessels and Draining Lymph Nodes of Arthritic Joints in Mice.. J. Physiol. 2014, 592 (6), 1213–1223. 10.1113/jphysiol.2013.266700. 24421350 PMC3961082

