# Supplementary material for: Chromenylium Star Polymers: Merging Water Solubility and Stealth Properties with Shortwave Infrared Emissive Fluorophores
Source: ACS Cent Sci. 2024 Dec 21;11(2):208–18. doi: 10.1021/acscentsci.4c01570 (PMC11869135; doi:10.1021/acscentsci.4c01570)
Supplement: Supplementary file 1 — oc4c01570_si_001.pdf [file oc4c01570_si_001.pdf]

Supporting Information

for

Chromenylium Star Polymers: Merging Water Solubility and Stealth Properties with Shortwave Infrared Emissive Fluorophores

Emily B. Mobley[1], Eric Y. Lin[1], Ellen M. Sletten[1*]

[1] Department of Chemistry and Biochemistry, University of California, Los Angeles, Los Angeles, CA 90095, United States

*email: sletten@chem.ucla.edu

Table of Contents

I. Supporting Figures 4
II. Supporting Schemes 30
III. Supporting Tables 32
IV. List of Supporting Videos 35
V. General Experimental Procedures and Materials 36
General Abbreviations 36
General Materials Handling and Storage 36
CStar GPC Calibration 36
Photophysical Instrumentation 37
Determination of Fluorescence Quantum Yield 37
Determination of Absorption Coefficient 38
Determination of Copper Concentration 38
SDS-PAGE Assay 38
Native PAGE Assay 38
Fluorophore Stability Assay 39
Photostability Assay 39
Cell Toxicity Assay 39
Animal Procedures 40
SWIR Imaging Apparatus 40
Image Processing 41
VI. Synthetic Procedures and Characterization 43
Synthetic Abbreviations 43
Synthetic Materials 43
Synthetic Instrumentation 43
Synthetic Procedures 44
^{1}H NMR Spectra 52
^{13}C NMR Spectra 64
GPC Spectra 72
MALDI Spectra 76
VII. Supporting References 80
VIII. Appendix A 82
Single Channel Images for Excitation-Multiplexing Experiments 82
IX. Appendix B 90

GPC Calibration via Poly(2-methyl-2-oxazoline) (POx) Polymers 90
MALDI spectra for POx standards 91
GPC spectra for POx standards... 93
^{1}H NMR spectra for POx standards 95
Appendix B References 99

I. Supporting Figures

A.

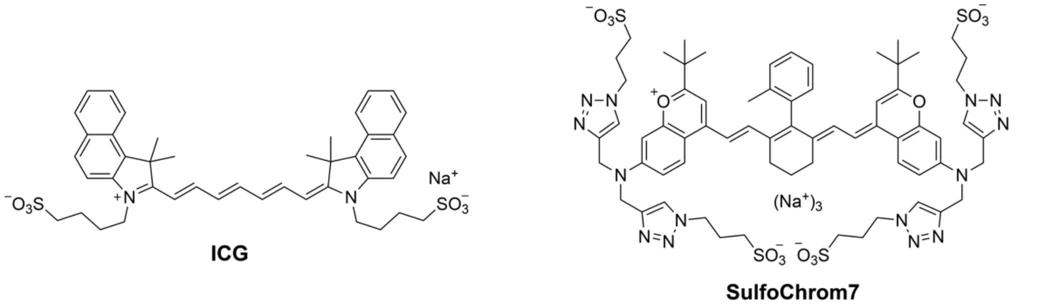

B.

Fluorophore	Solvent	$\lambda_{max, abs}$ (nm)	ε_{max} ($M^{-1}cm^{-1}$)	$\lambda_{max, em}$ (nm)	Φ_F (%)	Brightness ($M^{-1}cm^{-1}$)
**ICG**[a]	EtOH	787	223,000 ± 6,000	815	14 ± 1	31,000 ± 2,000
	H_2O	779	156,000 ± 6,000	805	2.9 ± 0.2	4,500 ± 400
	FBS	798	162,000 ± 3,000	811	12.0 ± 0.9	19,000 ± 2,000
**SulfoChrom7**[b]	MeOH	938	165,000 ± 2,000	964	1.06 ± 0.02	1,750 ± 40
	H_2O	923[c]	24,000[c,d]	941	n.d.[e]	-
	FBS	963	95,000[d]	976	0.56± 0.05	500[d]

a: Previously reported values[1]
b: Previously reported values[2]
c: Reported at the second maximum (monomer absorption)
d: No error reported
e: Not determined (n.d.) due to strong aggregation

Figure S1: Chemical structures (A) and previously reported photophysical properties (B) of **ICG**[1] and **SulfoChrom7**.[2]

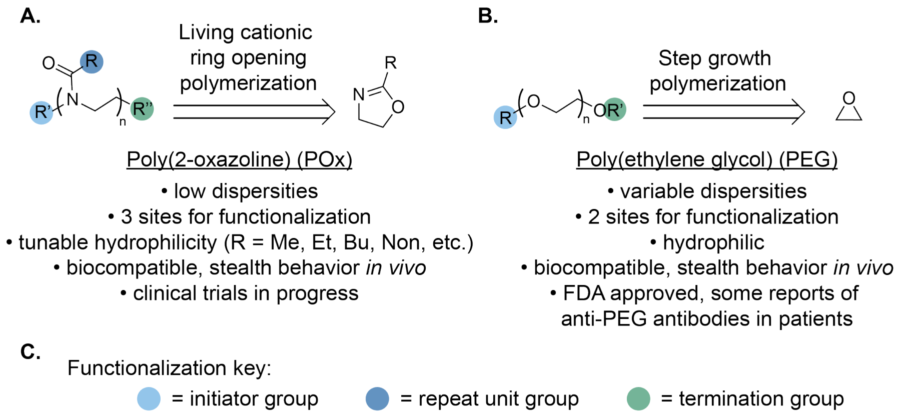

Figure S2: Comparison of poly(2-oxazoline) (POx) and poly(ethylene glycol) (PEG) stealth polymers.[3] (A–B) Synthesis and properties of POx (A) and PEG (B) polymers. (C) Key for functionalization sites in (A, B).

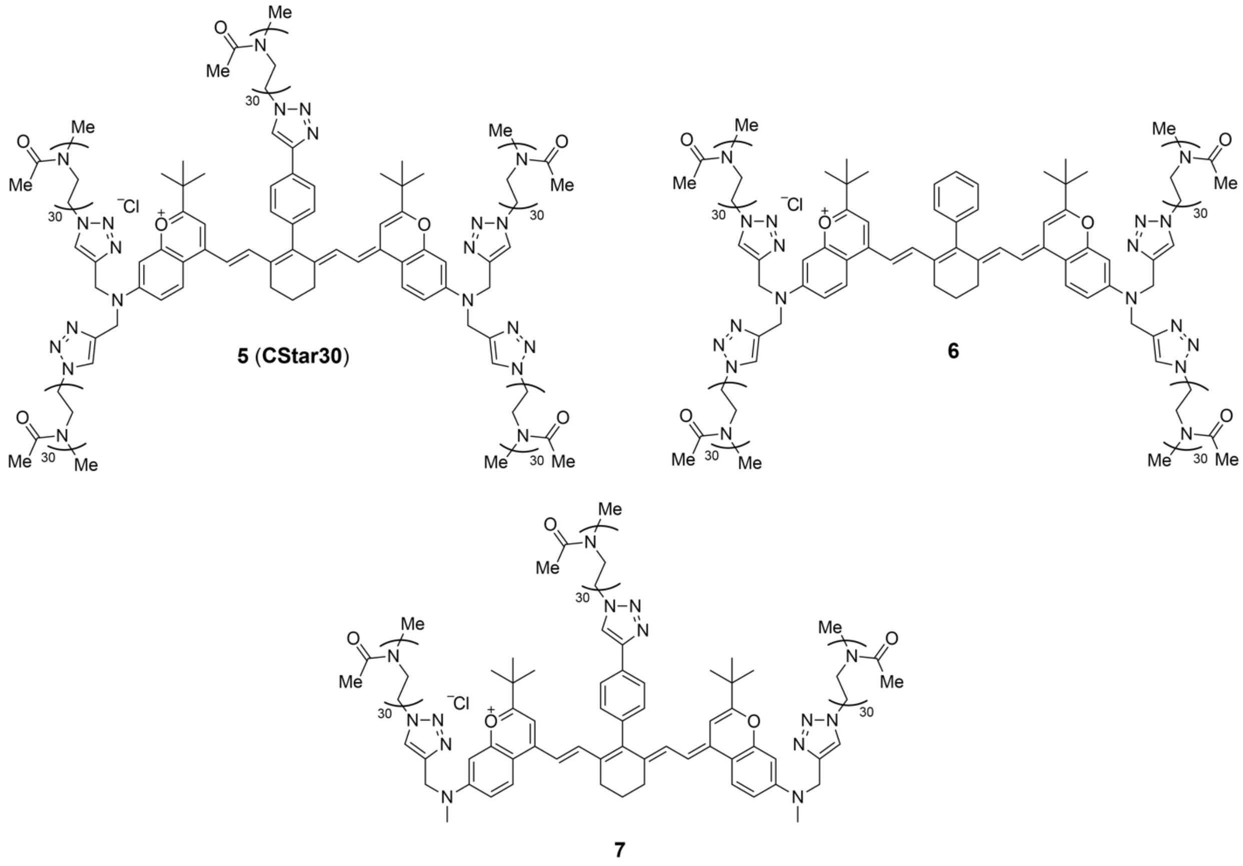

Figure S3: Chemical structures of CStars **5**–**7**.

A.

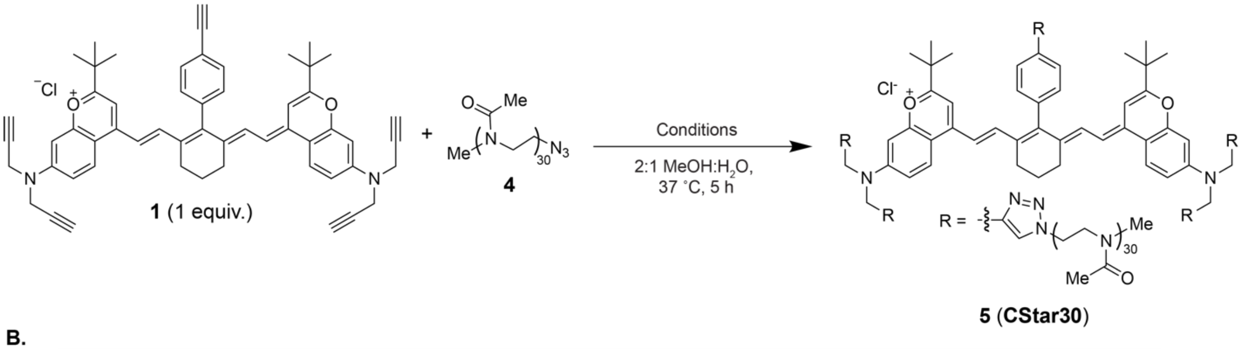

B.

Entry	1 (mM)	Equiv. 4	% Cu(II)	Equiv. THPTA	Equiv. NaAsc	Equiv. DIPEA	Notes
1	1.2	20	8.0	0.4	4.0	-	1-3 species[a]
2	1.2	5	8.0	0.4	4.0	-	**1** degrades
3	1.2	10	8.0	0.4	4.0	-	Same as Entry 1
4	0.60	10	8.0	0.4	4.0	-	1-2 species[a]
5	0.60	10	16	0.8	8.0	-	1 species[a]
6	0.60	10	8.0	0.4	4.0	10	1-4 species[a], promotes bis-dipole of **1**
7	0.60	10	16	0.8	8.0	10	Same as Entry 6

a: Measured by GPC, dUV 550 nm

C.

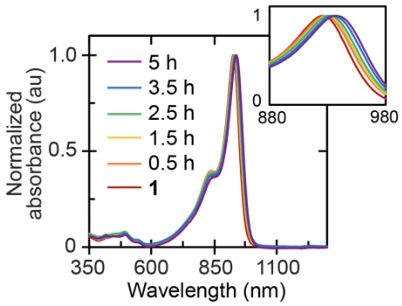

D.

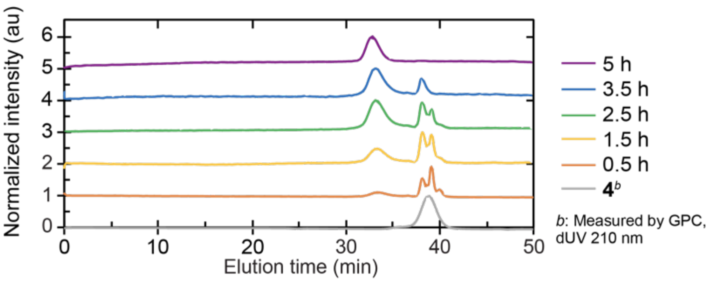

Figure S4: Optimization of CStar synthesis. (A) Synthesis of **5** (**CStar30**) was used to optimize reaction conditions of all CStars (**5**–**7**) at 0.5 mg scale of **1**. (B) Conditions screen for synthesis of **5** (**CStar30**) varying final concentration of **1** (1 equiv.) in solution, equiv. polymer **4**, THPTA ligand, NaAsc, and DIPEA, and % Cu catalyst loading. Entry 5 was found to be the optimal set of conditions. (C) Crude reaction absorbance (in MeOH) with the conditions from Entry 5 showing a gradual bathochromic shift (927 nm to 939 nm) over the course of the 5 h reaction, corresponding to the formation of **5**. Inset with the x-axis scaled from 880 to 980 nm. Measurements were taken in a 1 cm cuvette. (D) Corresponding crude reaction GPC (dUV 550 nm) to absorbance in (C) show a gradual increase in a larger molecular weight species (earlier elution time) over the course of the 5 h reaction, corresponding to the formation of **5**.

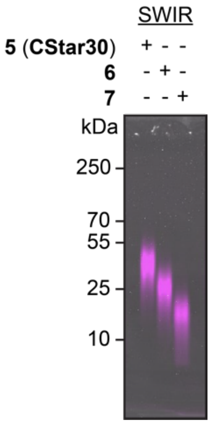

Figure S5: SDS-PAGE of CStars **5**–**7**. SWIR fluorescence was measured via InGaAs camera (merged two-color image: grey = brightfield [no laser], 100 ms ET, 1100 nm LP; magenta = 974 nm ex, 100 mW/cm^2, 10 ms ET, 1100 nm LP).

Discussion: To visualize the size separation of CStars **5**–**7**, an SDS-PAGE gel was run. CStars **5**–**7** are well-separated, decreasing in size from CStar **5** to **6** to **7**. Note that CStars **5**–**7** do not bind Coomassie, hence SWIR imaging was used to detect the relative gel migration of CStars. A pre-stained protein ladder was also visualized via a gel imaging system. We acknowledge that the molecular weights obtained by SDS-PAGE do not match the MALDI and GPC M_n, likely due to the non-protein like nature of the CStars.

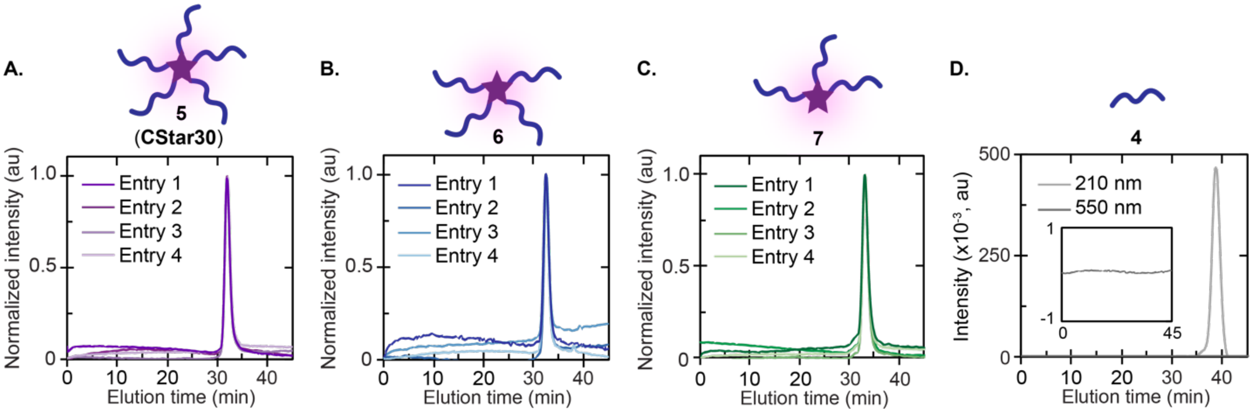

E.

	Entry	M_p (Da)	M_n (Da)	M_w (Da)	$Đ$ (M_w/M_n)
**4**[a]	-	2,315	2,498	2,654	1.06
**5** (**CStar30**)	1	12,854	12,284	12,523	1.02
	2	12,905	12,263	12,507	1.02
	3	12,982	12,380	12,622	1.02
	4	12,649	12,218	12,367	1.01
**6**	1	11,372	11,056	11,183	1.01
	2	11,272	11,085	11,214	1.01
	3	11,340	11,188	11,315	1.01
	4	11,430	11,179	11,345	1.01
**7**	1	9,473	9,148	9,288	1.02
	2	9,500	9,153	9,301	1.01
	3	9,421	9,197	9,330	1.01
	4	9,347	9,045	9,190	1.02

a: Not detectable by GPC, dUV 550 nm (see D). Measured by GPC, dUV 210 nm

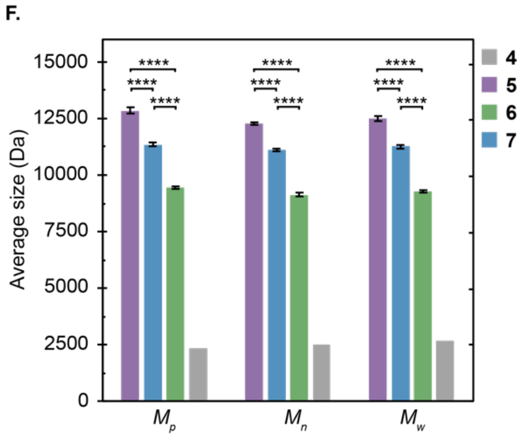

Figure S6: Size comparison of CStars **5**–**7** and purified POx polymer **4** via GPC. (A–C) GPC of crude CStar **5** (**CStar30**) (A), **6** (B), and **7** (C) reactions, with four replicates per CStar (dUV 550 nm) following optimized reaction conditions (1 mg scale of **1**, see Entry 5 in Figure S4). (D) GPC of POx polymer **4** (dUV 210 nm, inset dUV 550 nm with the y-axis scaled from -1 to 1). (E) Table of size parameters calculated by GPC, corresponding to spectra in (A–D). (F) Mean size parameters of CStars **5**–**7** compared to POx polymer **4**, corresponding to data in (E). One-way ANOVA tests amongst CStars **5**–**7** for M_p, M_n, and M_w all exhibited p-values $\leq 1\text{x}10^{-11}$, and unpaired two-tailed t-tests between each group all exhibited p-values ≤ 0.00003 for M_p, M_n, and M_w. T-tests: * $p \leq 0.05$, ** $p \leq 0.01$, *** $p \leq 0.001$, **** $p \leq 0.0001$. Error bars represent the standard deviation (n = 4).

Discussion: Crude CStar (**5**–**7**) GPC traces measured at 550 nm in (A–C) demonstrate > 99% conversion to CStars **5**–**7** with excellent reproducibility. The POx polymer precursor (**4**) GPC trace measured at 550 nm in (D) shows baseline signal, indicating that the larger molecular weight species observed at 550 nm in (A–C) are covalently bound to fluorophore. The statistical analyses described above and plotted in (E) suggest that CStars **5**–**7** are molecularly discrete (i.e., the number of polymer arms clicked onto the core fluorophore was as predicted in all cases; see Figure 2C for overlayed GPC spectra of isolated materials with dUV 210 nm).

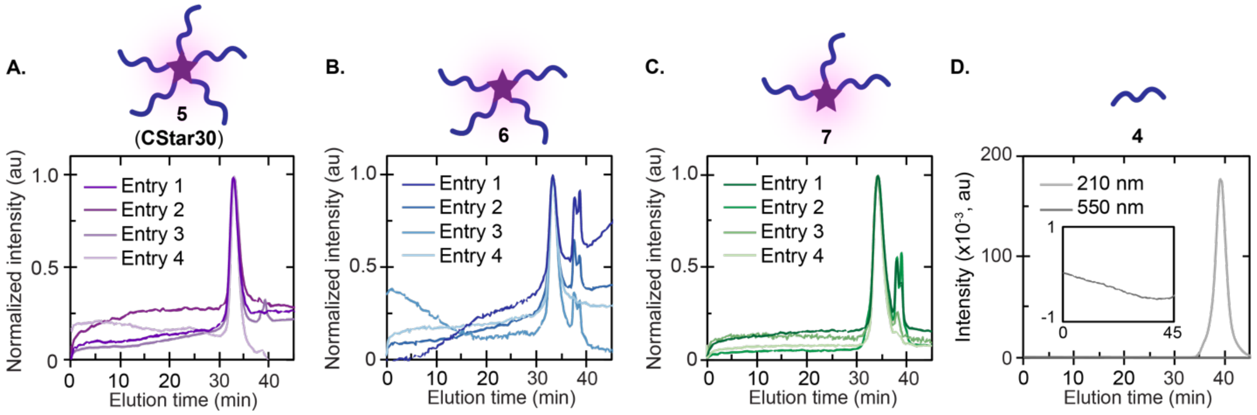

E.

	Entry	M_p (Da)	M_n (Da)	M_w (Da)	$Đ$ (M_w/M_n)
**4**[a]	-	2,249	2,138	2,363	1.11
**5**	1	8,877	8,373	8,588	1.02
	2	8,797	8,593	8,755	1.01
	3	8,813	8,441	8,628	1.02
	4	8,895	8,623	8,755	1.01
**6**	1	8,325	8,179	8,329	1.02
	2	8,510	8,242	8,380	1.02
	3	8,387	8,152	8,296	1.02
	4	8,341	8,203	8,341	1.03
**7**	1	6,963	6,383	6,647	1.04
	2	6,975	6,536	6,916	1.06
	3	7,210	6,837	7,004	1.02
	4	7,066	6,459	6,697	1.03

a: Not detectable by GPC, dUV 550 nm (see D). Measured by GPC, dUV 210 nm

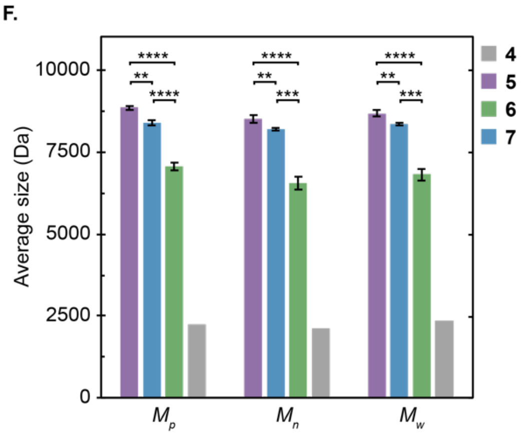

Figure S7: Replicate of size comparison of CStars **5**–**7** and purified POx polymer **4** via GPC. (A–C) GPC of crude CStar **5** (A), **6** (B), and **7** (C) reactions, with four replicates per CStar (dUV 550 nm) following optimized reaction conditions (1 mg scale of **1**, see Entry 5 in Figure S4). (D) GPC of POx polymer **4** (dUV 210 nm, inset dUV 550 nm with the y-axis scaled from -1 to 1). (E) Table of size parameters calculated by GPC, corresponding to spectra in (A–D). (F) Mean size parameters of CStars **5**–**7** compared to POx polymer **4**, corresponding to data in (E). One-way ANOVA tests amongst CStars **5**–**7** for M_p, M_n, and M_w all exhibited p-values ≤ 0.00000002, and unpaired two-tailed t-tests between each group all exhibited p-values ≤ 0.01 for M_p, M_n, and M_w. T-tests: * $p \leq 0.05$, ** $p \leq 0.01$, *** $p \leq 0.001$, **** $p \leq 0.0001$. Error bars represent the standard deviation ($n = 4$).

Discussion: Crude CStar (**5**–**7**) GPC traces measured at 550 nm in (A–C) demonstrate reduced conversion to CStars **5**–**7** using a more polydisperse POx polymer **4**. This suggests that the more monodisperse POx polymer **4** (used for experiments in Figure S6) is important for maximizing conversion and achieving target size of CStars. Still, the POx polymer precursor (**4**) GPC trace measured at 550 nm in (D) shows baseline signal, indicating that the larger molecular weight species observed at 550 nm in (A–C) are covalently bound to fluorophore. The statistical analyses described above and plotted in (E) also still suggest that CStars **5**–**7** are molecularly discrete (i.e., the number of polymer arms clicked onto the core fluorophore was as predicted in all cases).

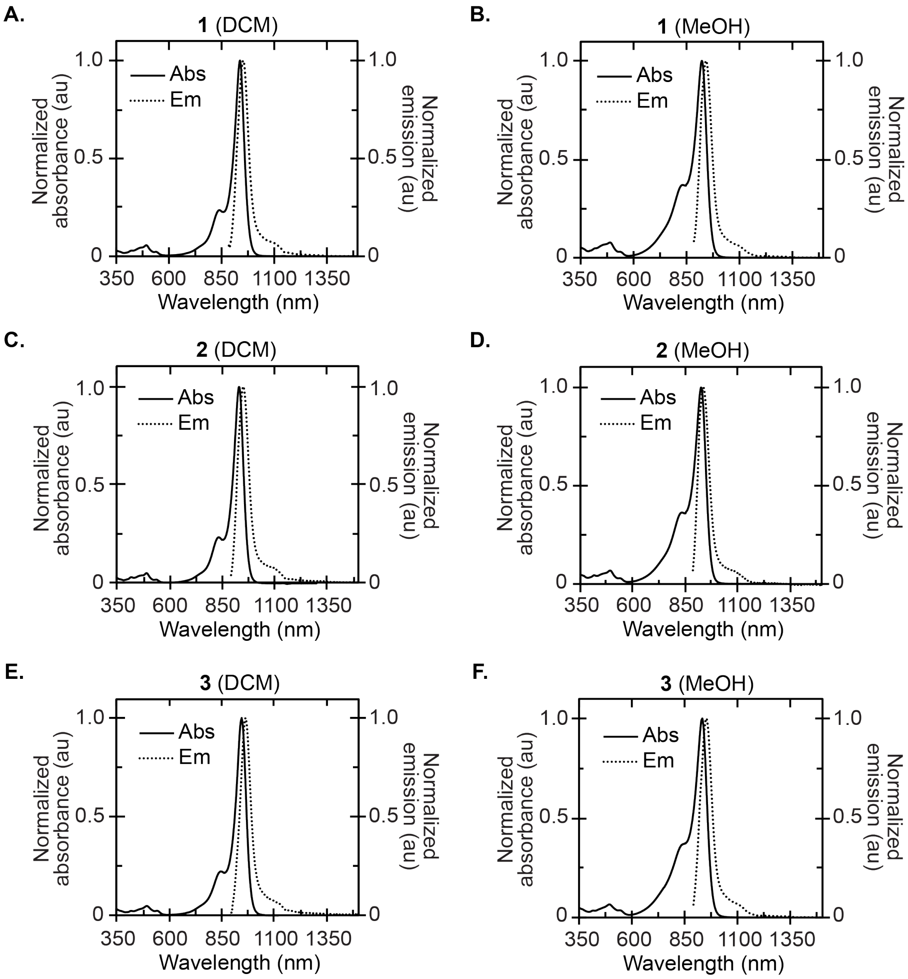

Figure S8: Absorbance and emission spectra of core fluorophores **1**–**3** in organic solvents. (A–B) Absorbance and emission of **1** in DCM (A) and MeOH (B). (C–D) Absorbance and emission of **2** in DCM (C) and MeOH (D). (E–F) Absorbance and emission of **3** in DCM (E) and MeOH (F). All samples are 4 µM, and measurements were taken in a 1 cm cuvette. Emission spectra: 860 nm ex, 885-1500 nm em. *Note*: emission spectra in DCM contain an artifact at 1150 nm, characteristic of the solvent.

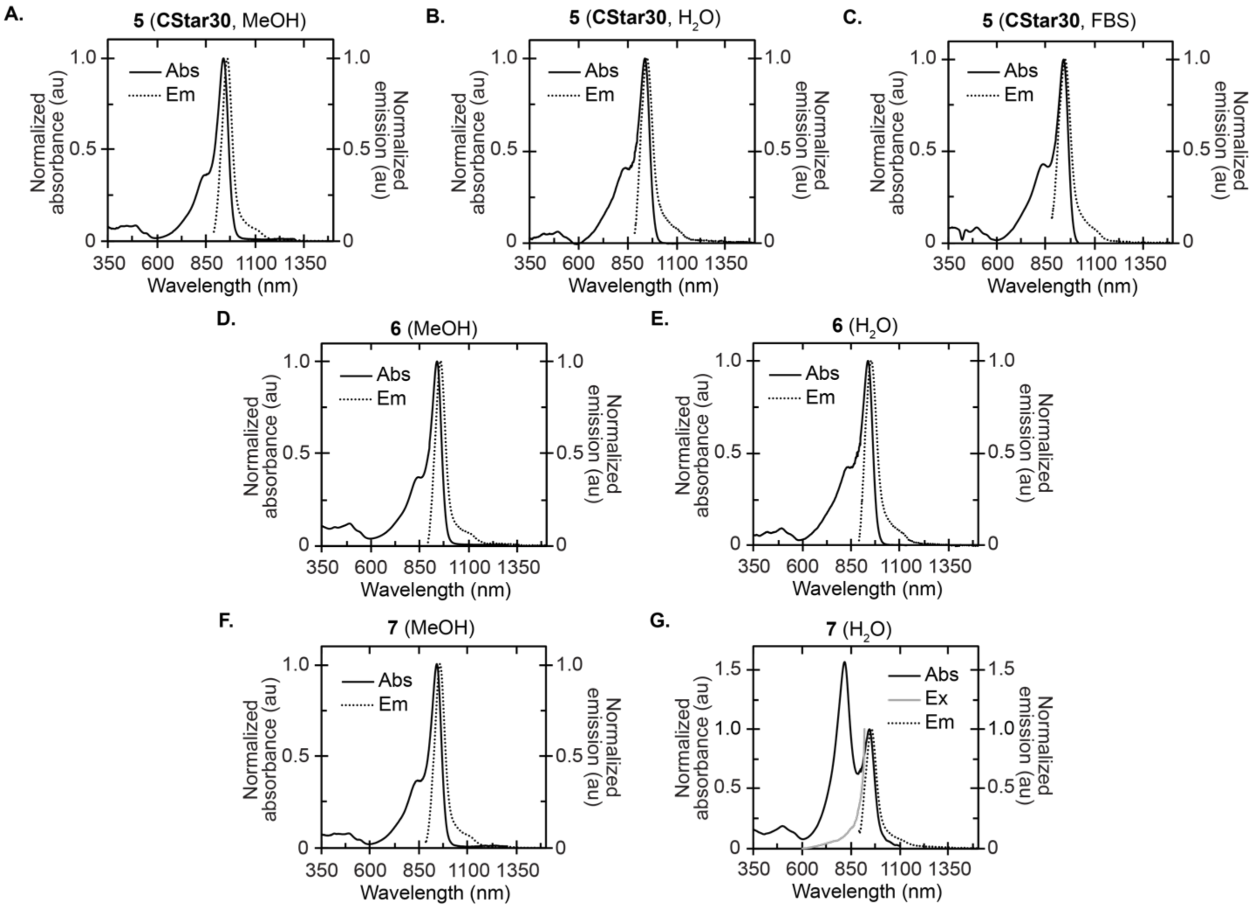

Figure S9: Absorbance and emission profiles of CStars **5**–**7** in organic and aqueous solvents. (A–C) Absorbance and emission of **5** (**CStar30**) in MeOH (A) and H_2O (B), and FBS (C). (D–E) Absorbance and emission of **6** in MeOH (D) and H_2O (E). (F) Absorbance and emission of **7** in MeOH. (G) Absorbance, excitation and emission of **7** in H_2O. All samples are 4 μM, and measurements were taken in a 3 mm cuvette. Emission spectra: 860 nm ex, 885-1500 nm em. Excitation spectra: 940 nm em, 600-920 nm ex.

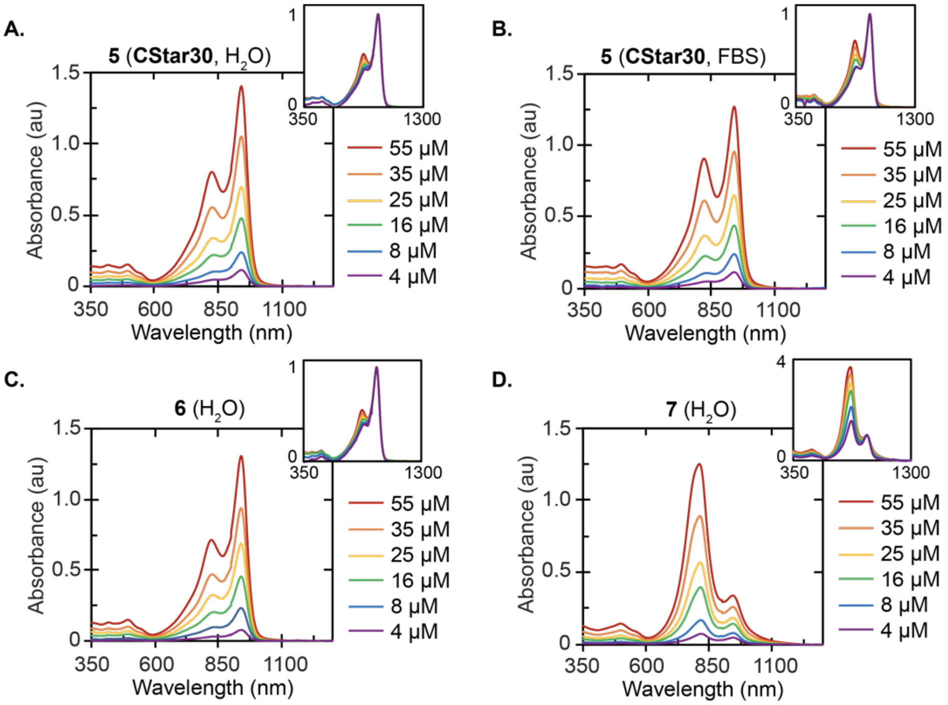

Figure S10: Concentration-dependent absorption profiles of CStars **5**–**7** in aqueous solvents. (A–B) Absorbance of **5** (**CStar30**) in H_2O, $R^2 = 0.9985$ (A) and FBS, $R^2 = 0.9995$ (B). (C) Absorbance of **6** in H_2O, $R^2 = 0.9945$. (D) Absorbance of **7** in H_2O, $R^2 = 0.9933$. All samples are 4–55 µM, and measurements were taken in a 3 mm cuvette. Linear relationship (R^2) was determined from monomer absorbance (au) as a function of concentration (µM). Insets are normalized to the monomer absorbance with y-axis scaled 0 to 1.1 (A–C) or 0 to 4 (D).

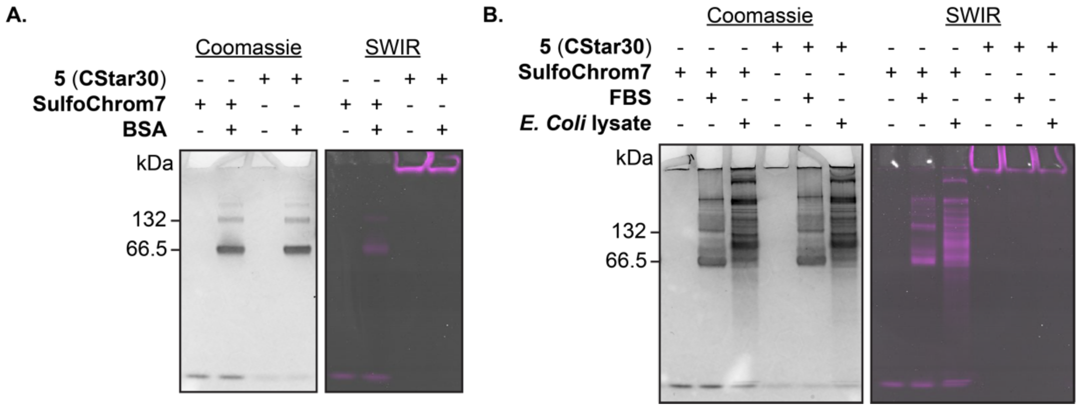

Figure S11: Native PAGE of **5** (**CStar30**) and **SulfoChrom7** in the presence of protein. (A) **CStar30** and **SulfoChrom7** with or without BSA incubation. Coomassie stain (left) and SWIR imaging (right) detection. Loading: 0.6 nmol fluorophore, with or without 5 µg BSA. (B) **CStar30** and **SulfoChrom7** with or without FBS/*E. Coli* cell lysate incubation. Coomassie stain (left) and SWIR imaging (right) detection. Loading: 0.6 nmol fluorophore, with or without 20 µg total protein. Coomassie stain was measured via a gel imaging system. All SWIR fluorescence was measured via InGaAs camera (merged two-color image: grey = brightfield [no laser], 100 ms ET, 1100 nm LP; magenta = 974 nm ex, 100 mW/cm^2, 100 ms ET, 1100 nm LP).

Discussion: To visualize protein association of fluorophores **5** (**CStar30**) and **SulfoChrom7**, native PAGE gels were run with each dye with and without protein incubation. Comparing free dye and dye/protein lanes, **SulfoChrom7** fluorescence overlays with BSA, with some dye remaining unbound. Conversely, there is no difference between the free dye and dye/protein lanes for any **CStar30** samples. It is worth noting that since **CStar30** carries a positive charge and does not bind Coomassie, the fluorophore was unable to run on the native PAGE gel without protein binding. On the other hand, **SulfoChrom7** is negatively charged and did not require protein or Coomassie binding to enter the gel matrix; the free dye runs with the loading buffer dye. Additionally, the sizes of individual proteins in FBS and *E. Coli* lysate were not crucial for data analysis, hence a BSA size standard was used in lieu of a protein ladder. Overall, we conclude that **CStar30** is able to broadly repel protein association.

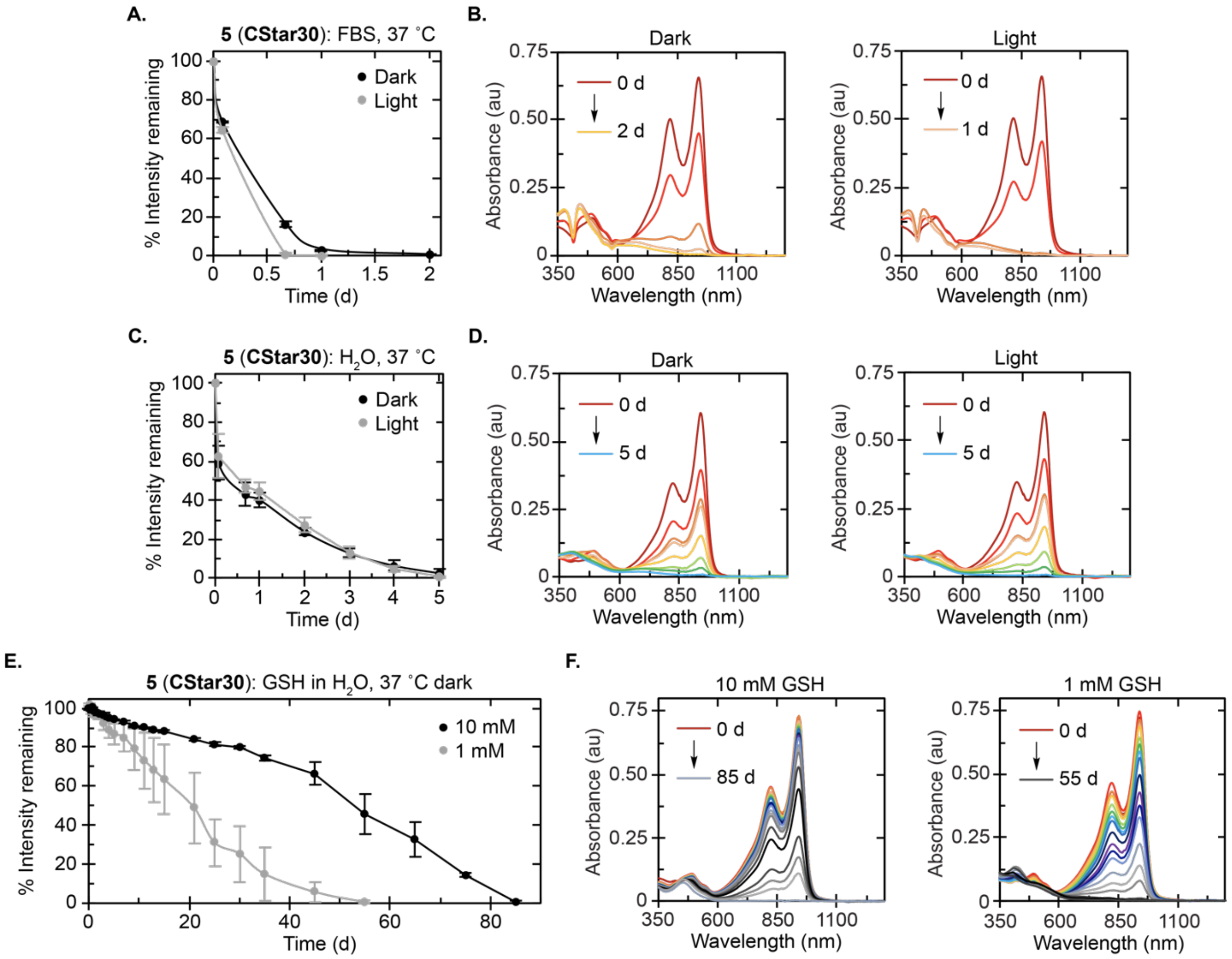

Figure S12: Stability of **5** (**CStar30**) in biologically relevant conditions. (A) Stability in FBS over time (37 ˚C, dark or ambient light). (B) Representative absorbance traces corresponding to timepoints in (A). (C) Stability in H_2O over time (37 ˚C, dark or ambient light). (D) Representative absorbance traces corresponding to timepoints in (C). (E) Stability in H_2O with GSH over time (37 ˚C, dark, 1 mM or 10 mM GSH). (F) Representative absorbance traces corresponding to timepoints in (E). All stock solutions are 0.6–0.75 O.D., and measurements were taken at the given timepoints in a 3 mm cuvette. Error bars represent the standard deviation (n = 3).

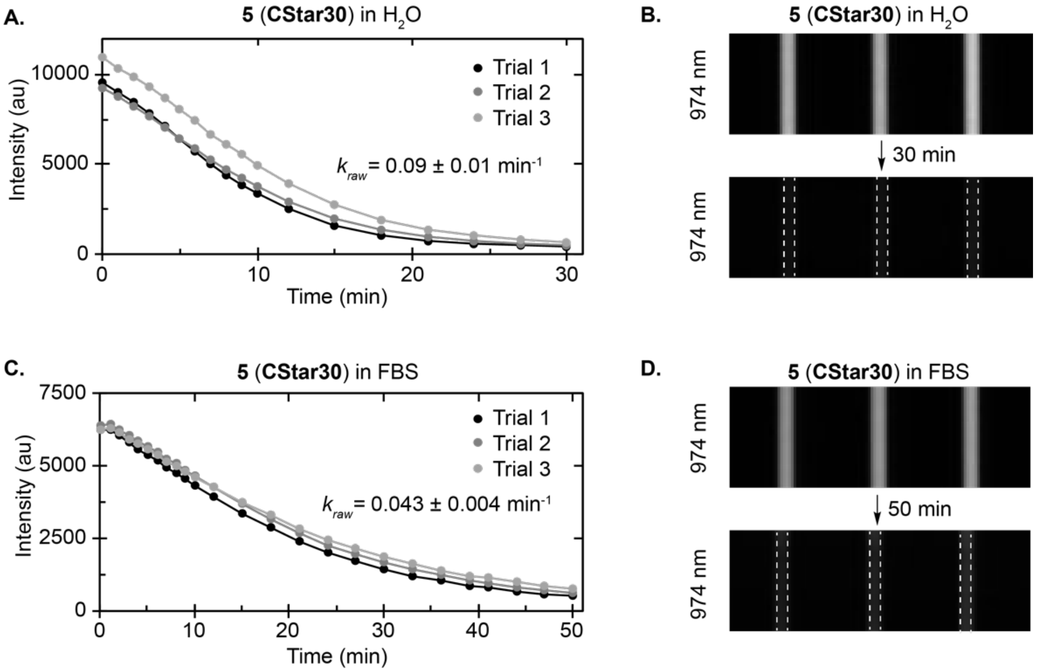

Figure S13: Raw photobleaching data of **5** (**CStar30**), corresponding to Figure 4F. (A) Photobleaching in H_2O. The rate was calculated to be 0.09 ± 0.01 min^{-1}. (B) Capillary images before and after photobleaching in H_2O (corresponding to intensity decay in [A]) measured via InGaAs camera (974 nm ex, 100 mW/cm^2, 5 ms ET, 1100 nm LP). (C) Photobleaching in FBS. The rate was calculated to be 0.043 ± 0.004 min^{-1}. (D) Capillary images before and after photobleaching in FBS (corresponding to intensity decay in [C]) measured via InGaAs camera (974 nm ex, 100 mW/cm^2, 5 ms ET, 1100 nm LP).

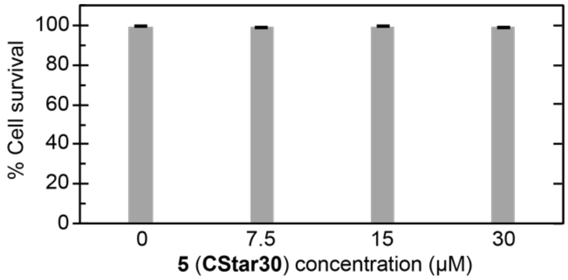

Figure S14: Cell toxicity of **5** (**CStar30**) via trypan blue exclusion assay. Cell survival of RAW 264.7 cells after 4 h incubation within media containing **CStar30** (7.5–30 μM; approximately 0.5x, 1x, and 2x of anticipated *in vivo* concentrations) or PBS as a control (0 μM). Error bars represent the standard deviation (n = 3).

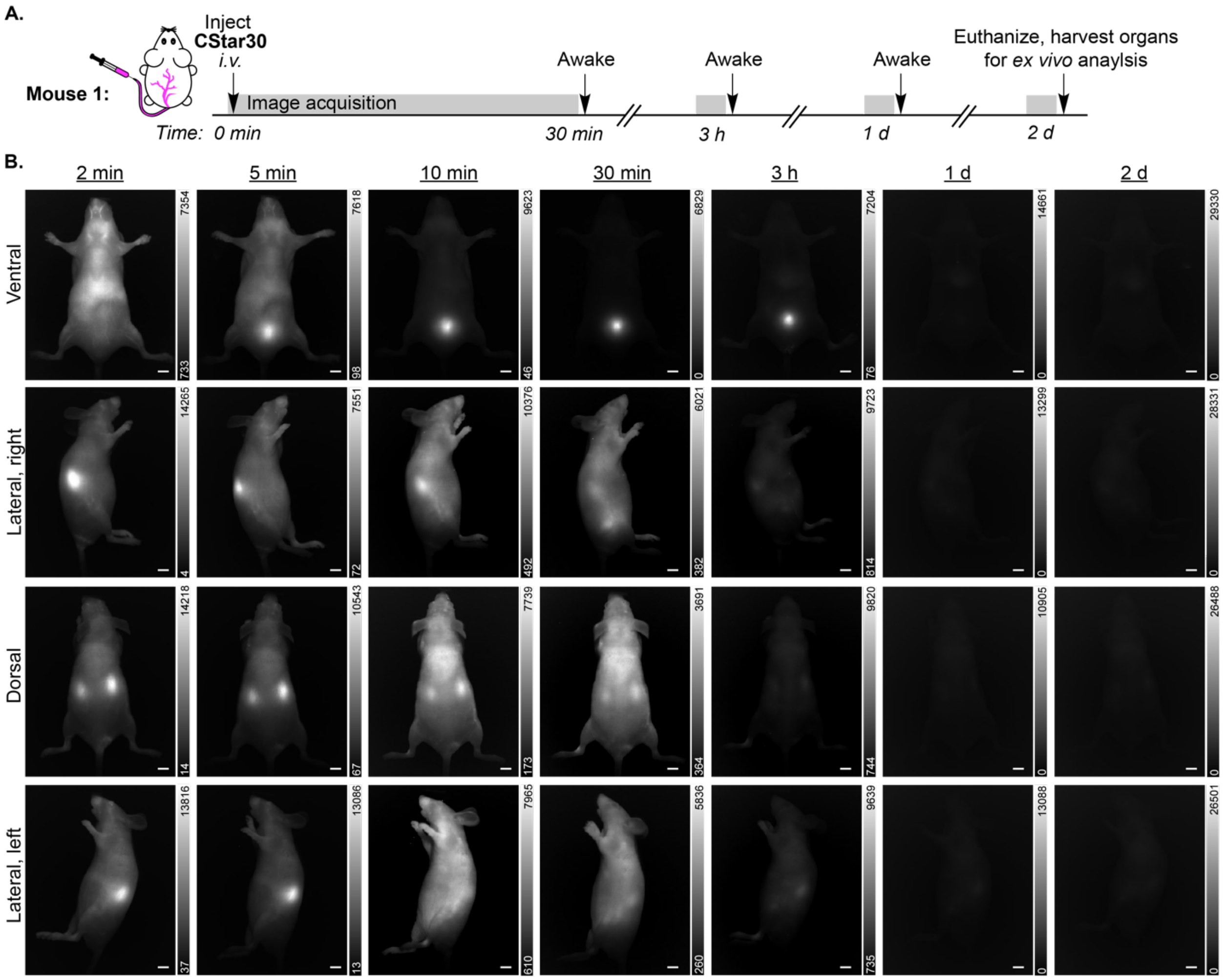

Figure S15: Single channel imaging timecourse of **5** (**CStar30**) injected *i.v.* in mice (replicate "Mouse 1"). (A) Injection scheme (200 µL, 30 nmol in sterile H_2O) and timeline. (B) Still images of the ventral, lateral (right, left) and dorsal views of the animal, timecourse from 2 min to 2 d post-injection, measured via InGaAs camera (974 nm ex, 160 mW/cm^2, 1100 nm LP, 20–100 fps). See Table S2 for sensitivity related parameters. Scale bars: 10 mm.

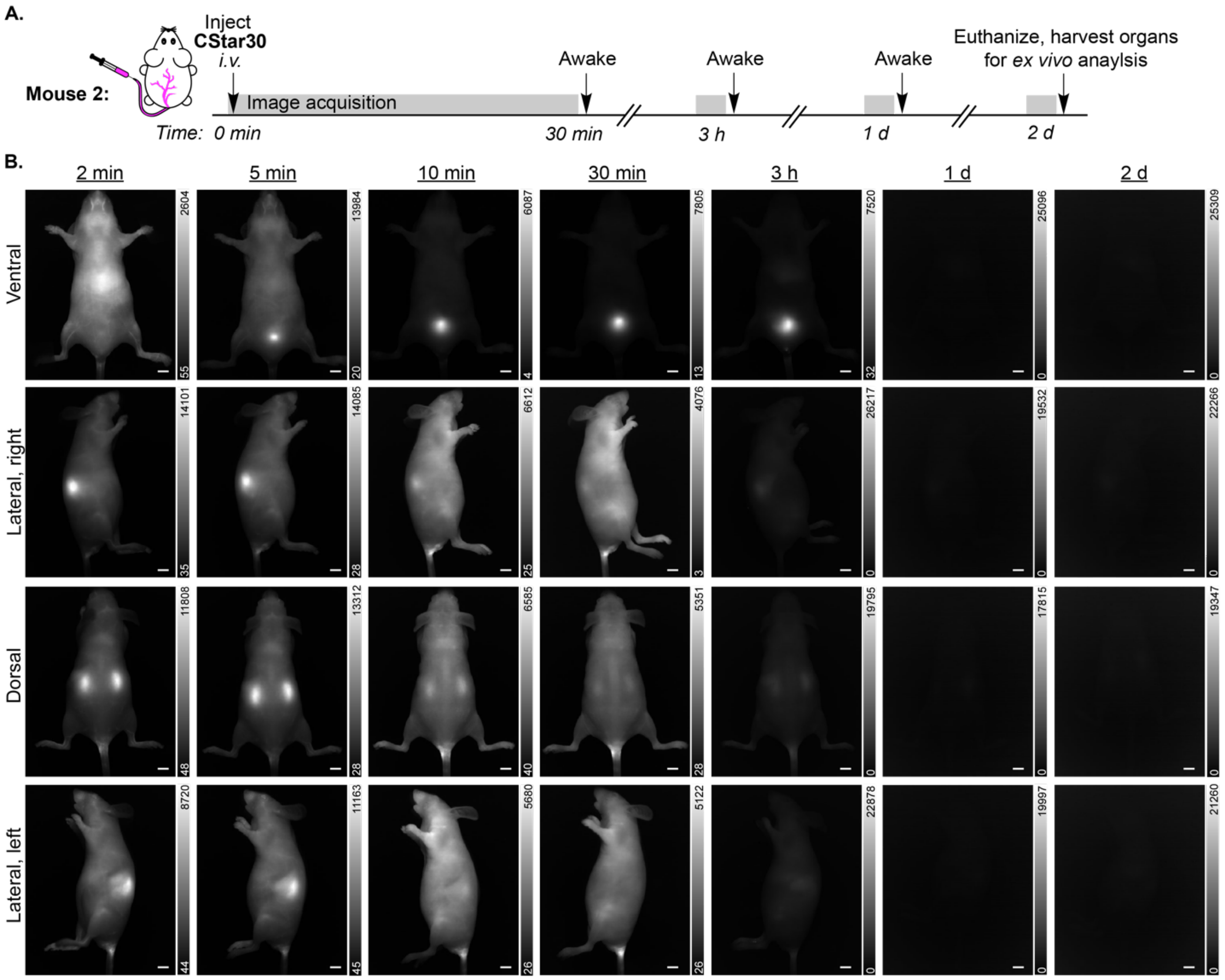

Figure S16: Single channel imaging timecourse of **5** (**CStar30**) injected *i.v.* in mice (replicate "Mouse 2"). (A) Injection scheme (200 µL, 30 nmol in sterile H_2O) and timeline. (B) Still images of the ventral, lateral (right, left) and dorsal views of the animal, timecourse from 2 min to 2 d post-injection, measured via InGaAs camera (974 nm ex, 160 mW/cm^2, 1100 nm LP, 33–100 fps). See Table S2 for sensitivity related parameters. Scale bars: 10 mm. See Video S1 for the dorsal view injection video.

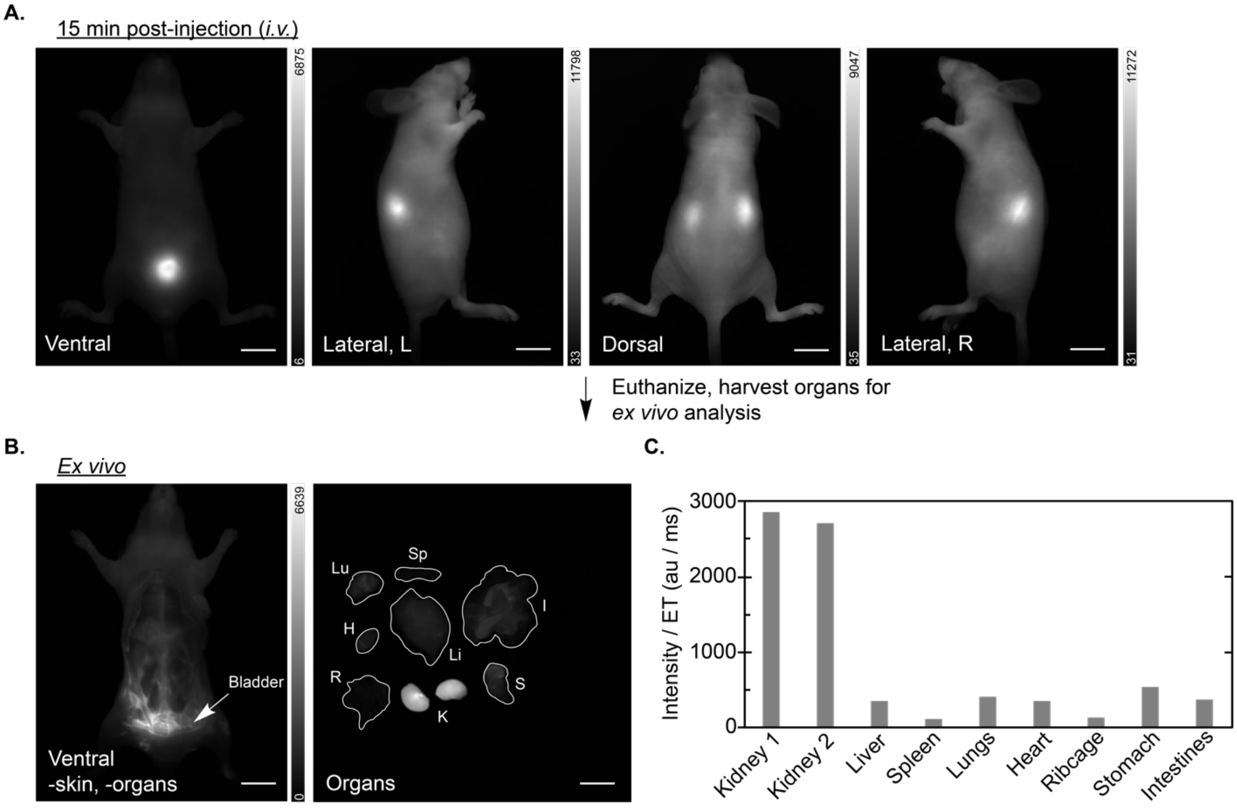

Figure S17: Estimating the blood circulation time of **CStar30** under anesthesia via *i.v.* injection. (A) Still images of the ventral, lateral (right, left) and dorsal views of the animal 15 min post-injection (200 µL, 30 nmol in sterile H_2O), measured via InGaAs camera (974 nm ex, 160 mW/cm^2, 1100 nm LP, 100 fps). See Table S2 for sensitivity related parameters. Scale bars: 10 mm. (B) *Ex vivo* images (euthanized after images in [A] were recorded) of the ventral side (left, skin and organs removed) and resected organs (right) measured via InGaAs camera (both images: 974 nm ex, 160 mW/cm^2, 1100 nm LP, 1 ms ET). Scale bars: 10 mm. *Organ key: H, heart; I, intestines; K, kidney; Li, liver; Lu, lungs; R, ribcage; S, stomach, Sp, spleen.* (C) Quantification of (B, right) for individual organs, normalized by ET.

Discussion: To estimate the blood circulation time of **CStar30** when introduced to mice via an *i.v.* injection, we first analyzed non-invasive, *in vivo* single color images of the replicate *i.v.* injection animals (see Figure 5, Figure S15, S16) for disappearance of vasculature signal post-injection. From these data, **CStar30** appears to complete flow out of the circulatory system and into the renal system ~10 min post-injection. To confirm this observation, another mouse was injected with **CStar30** and imaged until fluorescence in the vasculature was indistinguishable (A). At this time (15 minutes post-injection), the animal was euthanized and individual organs were carefully resected without external saline perfusion, to preserve any **CStar30** within the vasculature. The bladder was quite delicate and burst when resection was attempted, evident by the fluorescence signal from **CStar30** throughout the lower body cavity (B, left image). Analyzing fluorescence in the mouse carcass and individual resected organs (B, C), there was no significant perfusion of **CStar30** in any veins or major organs, apart from the kidneys and bladder. This observation is consistent with a blood circulation time on the order of 10–15 min for **CStar30** while under anesthesia.

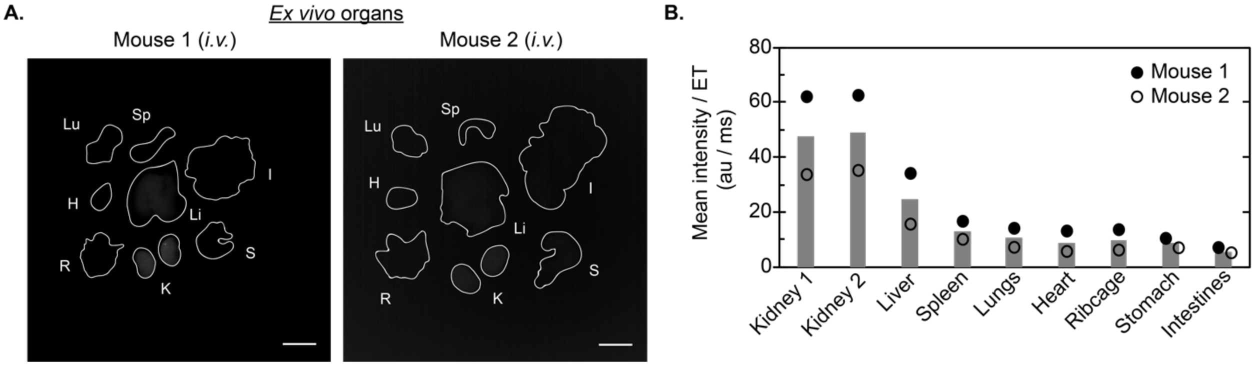

Figure S18: *Ex vivo* organs from single channel imaging of **5** (**CStar30**) injected *i.v.*, "Mouse 1" and "Mouse 2" after 2 d post-injection. (A) Fluorescence of resected organs, measured via InGaAs camera (974 nm ex, 160 mW/cm^2, 10 ms ET, 1100 nm LP). (B) Quantification of (A) for individual organs, normalized by ET. Scale bars: 10 mm. *Organ key: H, heart; I, intestines; K, kidney; Li, liver; Lu, lungs; R, ribcage; S, stomach, Sp, spleen.*

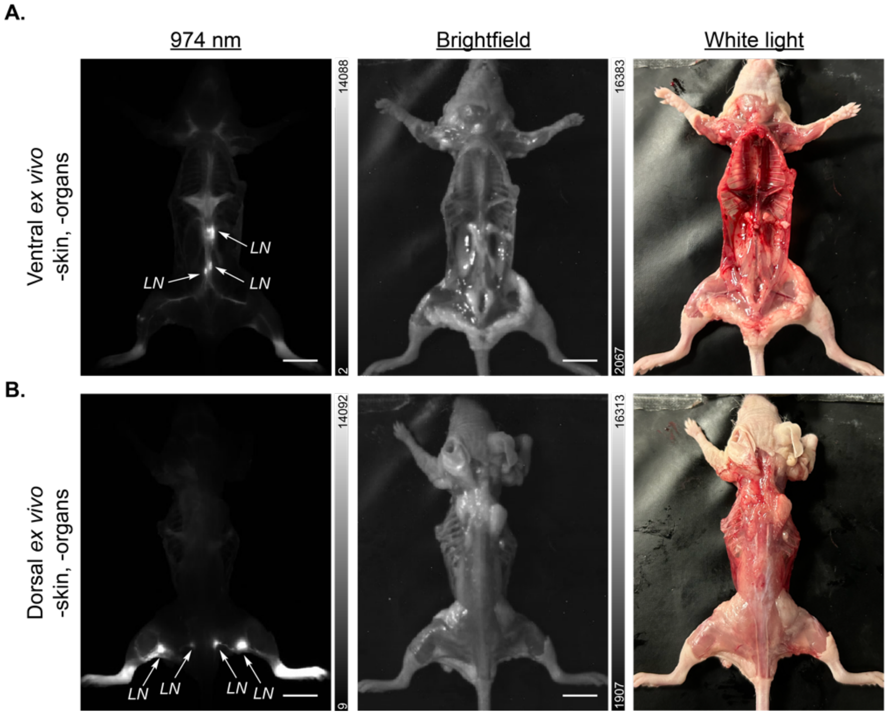

Figure S19: SWIR *vs*. brightfield *vs*. white light contrast for lymph node identification. (A–B) *Ex vivo* images (euthanized 5 min post-injection, hind feet uncovered, skin and organs removed) of the ventral (A) and dorsal side (B). Brightfield (ambient light, 100 ms ET, 1100 nm LP) and 974 nm (974 nm ex, 160 mW/cm^2, 3 ms ET, 1100 nm LP) measured via InGaAs camera. White light

recorded with an iPhone 14 camera. See Video S2 for image-guided lymph node resection. Scale bars: 10 mm. *Organ key: LN, lymph node.*

Discussion: Comparing the images collected via InGaAs camera (974 nm excitation versus brightfield with an 1100 nm LP filter) and iPhone 14 camera (white light), drastically different levels of contrast are observed for lymph node detection. To the naked eye, lymph nodes are virtually indistinguishable from other tissues due to their small size. Visualizing lymph nodes via fluorophore labeling with SWIR detection provides the necessary contrast to identify and resect lymph nodes, which is an important metric for disease staging.

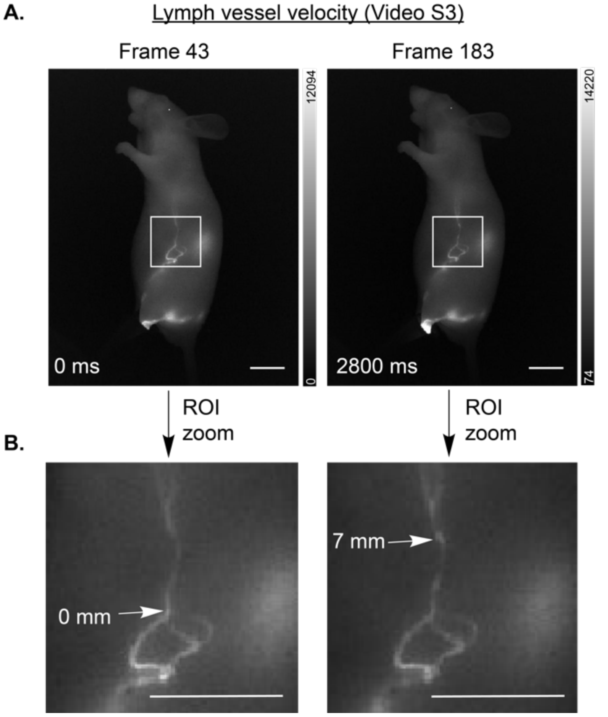

Figure S20: Single frames from Video S3 used for lymphatic vessel velocity calculation, with inhalation of 2–4% isoflurane/oxygen. (A) Still *in vivo* images of frame 43 (time = 0 ms) and frame 183 (time = 2800 ms) of the lateral left side of the animal. (B) Square ROI from (A) zoomed in to track fluorescence traversing lymph vessel at each frame. A horizontal section was chosen, with a distance of 7 mm traveled in 2800 ms. A lymph vessel velocity of 2.5 mm/s was calculated from these frames. Images correspond to injection of "Mouse 1" at the 20 min timepoint (see Figure 5D, S18, Video S3). All images were measured via InGaAs camera (974 nm ex, 160 mW/cm^2, 20 ms ET, 1100 nm LP). Scale bars: 10 mm.

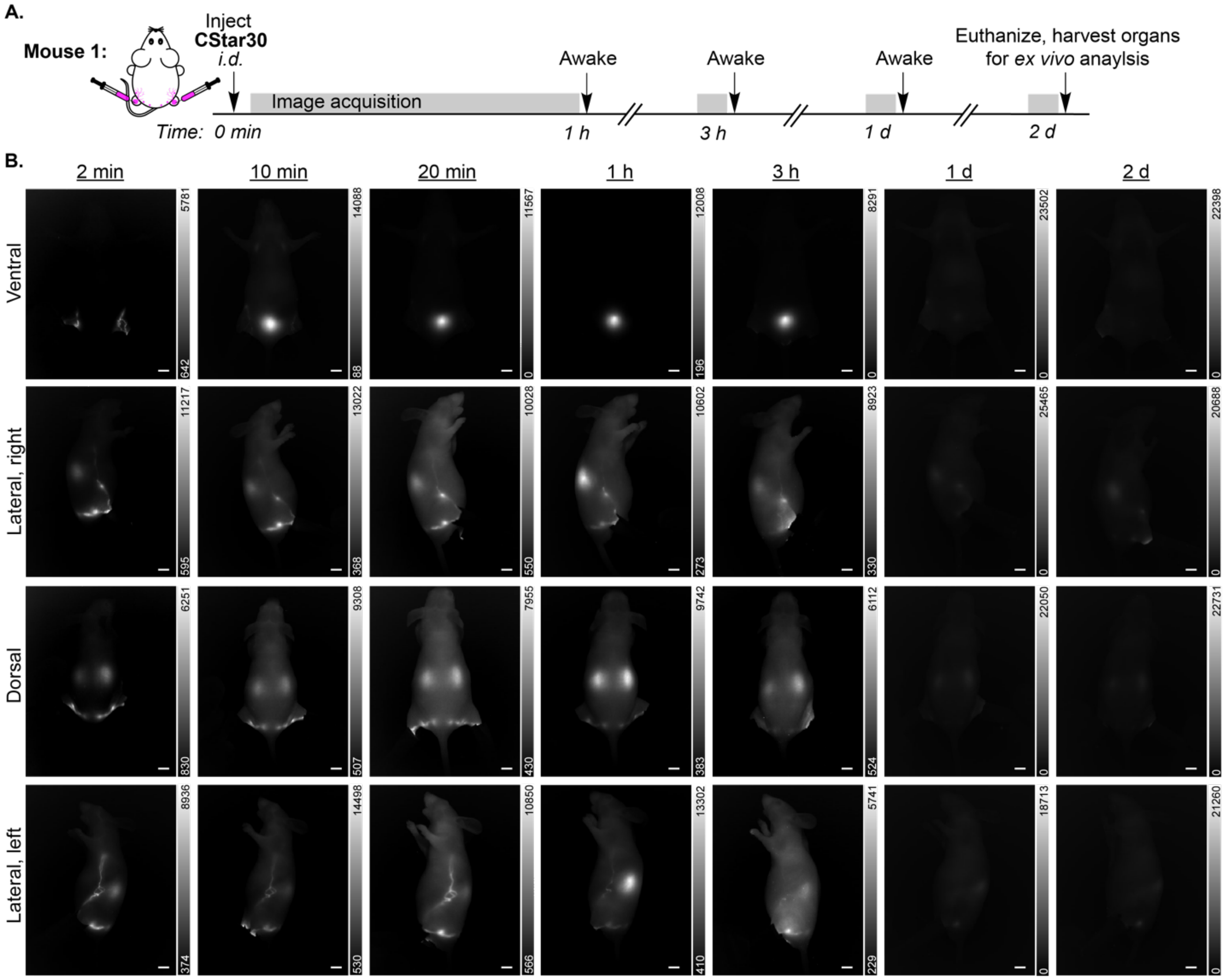

Figure S21: Single channel imaging timecourse of **5** (**CStar30**) injected *i.d.* in mice (replicate "Mouse 1"). (A) Injection scheme (50 µL each hind footpad, 30 nmol total in sterile H_2O) and timeline. (B) Still images of the ventral, lateral (right, left) and dorsal views of the animal, timecourse from 2 min to 2 d post-injection, measured via InGaAs camera (974 nm ex, 160 mW/cm^2, 1100 nm LP, 50–100 fps). See Table S2 for sensitivity related parameters. Scale bars: 10 mm.

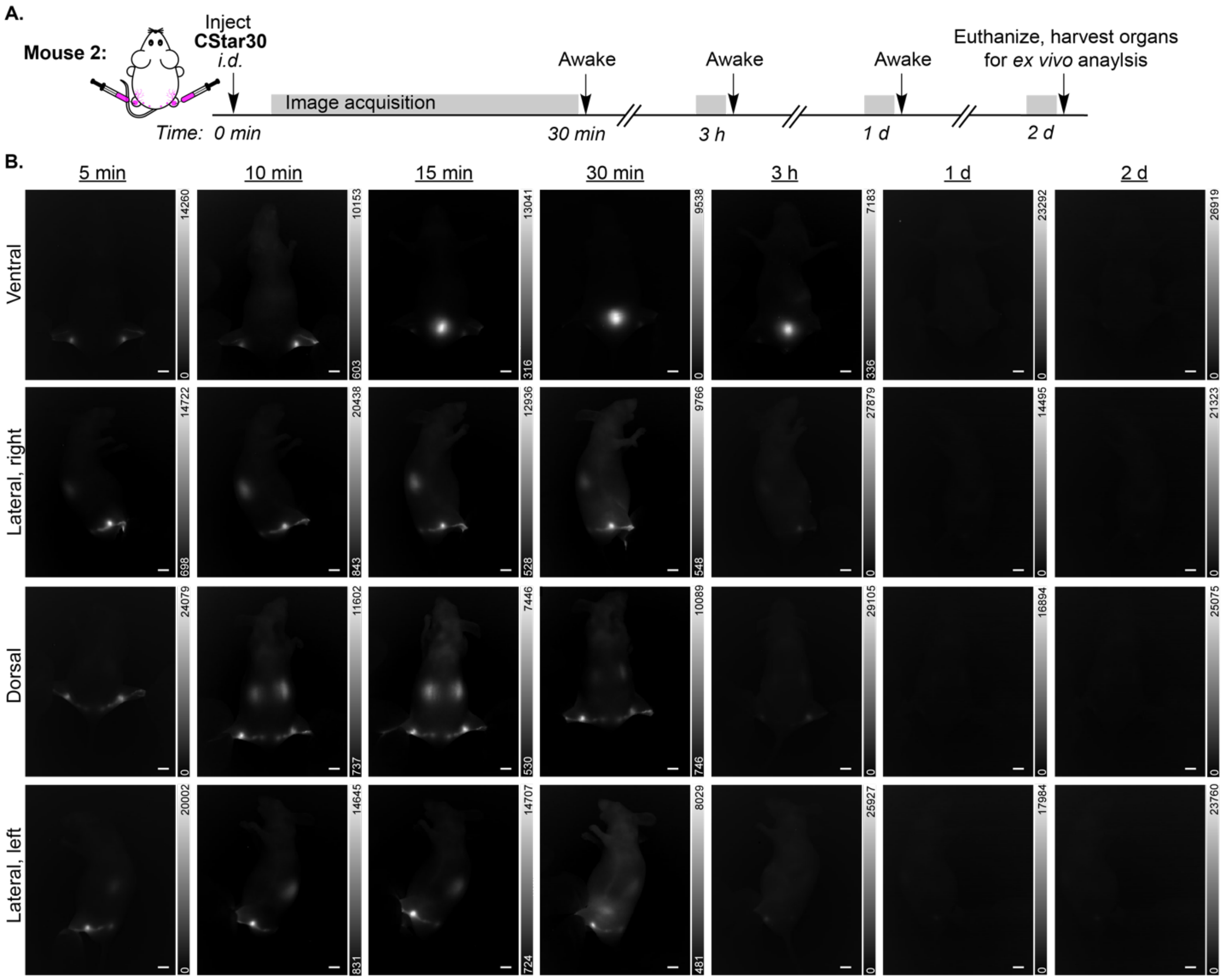

Figure S22: Single channel imaging timecourse of **5** (**CStar30**) injected *i.d.* in mice (replicate "Mouse 2"). (A) Injection scheme (50 µL each hind footpad, 30 nmol total in sterile H_2O) and timeline. (B) Still images of the ventral, lateral (right, left) and dorsal views of the animal, timecourse from 2 min to 2 d post-injection, measured via InGaAs camera (974 nm ex, 160 mW/cm^2, 1100 nm LP, 33–100 fps). See Table S2 for sensitivity related parameters. Scale bars: 10 mm.

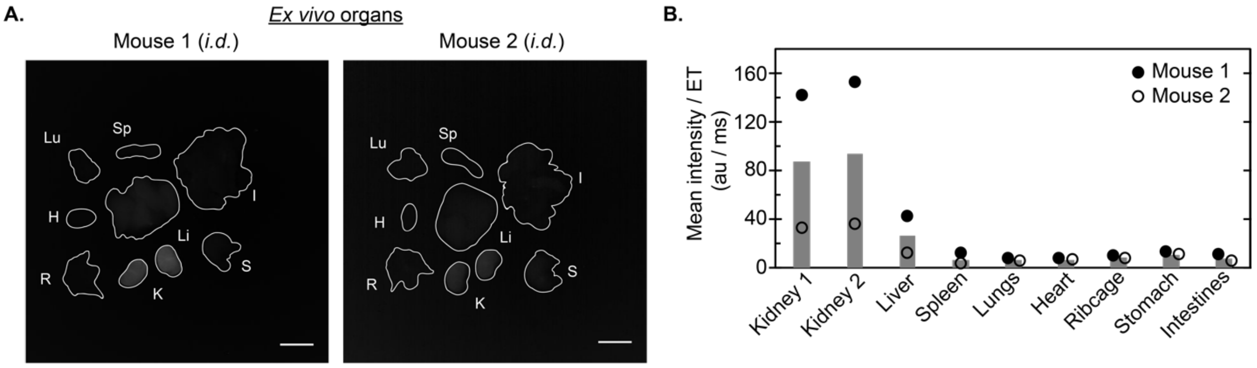

Figure S23: *Ex vivo* organs from single channel imaging of **5** (**CStar30**) injected *i.d.*, "Mouse 1" and "Mouse 2" after 2 d post-injection. (A) Fluorescence of resected organs, measured via InGaAs camera (974 nm ex, 160 mW/cm^2, 15 ms ET, 1100 nm LP). (B) Quantification of (A) for individual organs, normalized by ET. Scale bars: 10 mm. *Organ key: H, heart; I, intestines; K, kidney; Li, liver; Lu, lungs; R, ribcage; S, stomach, Sp, spleen.*

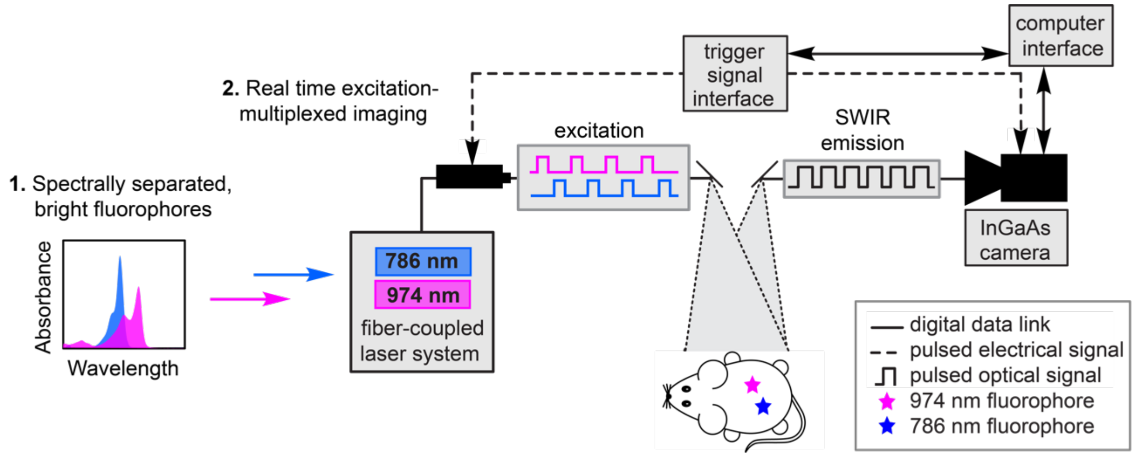

Figure S24: Real time excitation-multiplexed imaging scheme[4] for **5** (**CStar30**, magenta) and **ICG** (blue). Briefly, spectrally separated, bright fluorophores that are well-aligned to commercial laser lines (786 nm and 974 nm) are alternately excited through a pulsed/triggered laser system. Emission from each channel is collected simultaneously with a single SWIR emission window via InGaAs camera. The entire setup is controlled by an external computer interface.

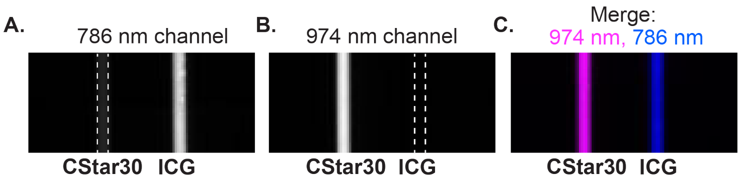

Figure S25: Capillary images of **5** (**CStar30**, 15 µM) in H_2O and **ICG** (100 µM) in FBS measured via InGaAs camera, in preparation for two-color multiplexed imaging in mice. (A) Single channel image with 786 nm ex (100 mW/cm^2, 1 ms ET, 1100 nm LP). (B) Single channel image with 974 nm ex (160 mW/cm^2, 5 ms ET, 1100 nm LP). (C) Merged 786 nm (blue) and 974 nm (magenta) channels.

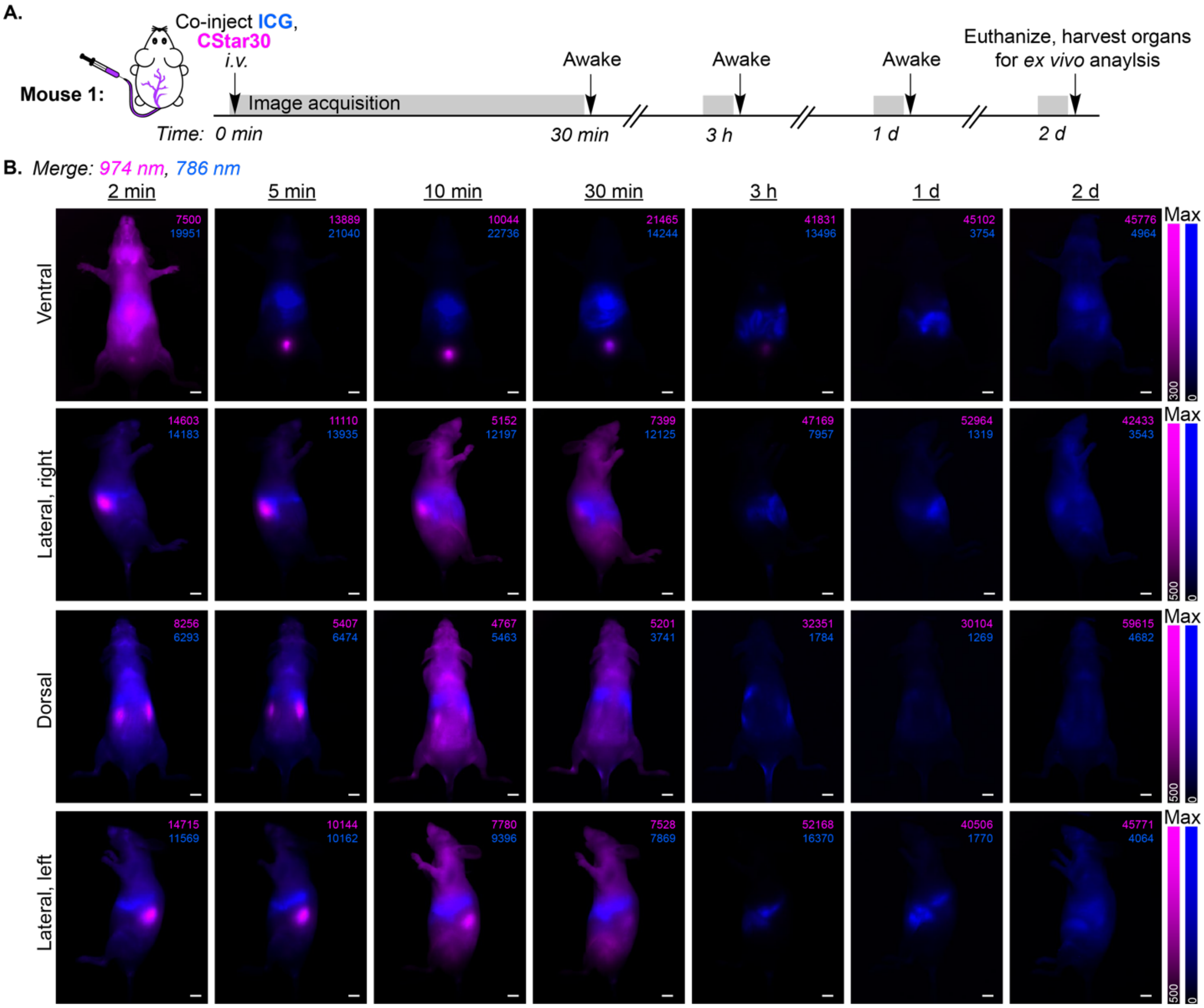

Figure S26: Two-color imaging timecourse of **5** (**CStar30**) and **ICG** co-injected *i.v.* in mice (replicate "Mouse 1"). (A) Injection scheme (200 µL, 30 nmol **CStar30** and 200 nmol **ICG** in

sterile H_2O) and timeline. (B) Still images of the ventral, lateral (right, left) and dorsal views of the animal, timecourse from 2 min to 2 d post-injection, measured via InGaAs camera (786 nm and 974 nm ex, 100-160 mW/cm^2, 1100 nm LP, 15–100 fps). See Table S3 for sensitivity related parameters. Max intensity: upper right of each image. Scale bars: 10 mm.

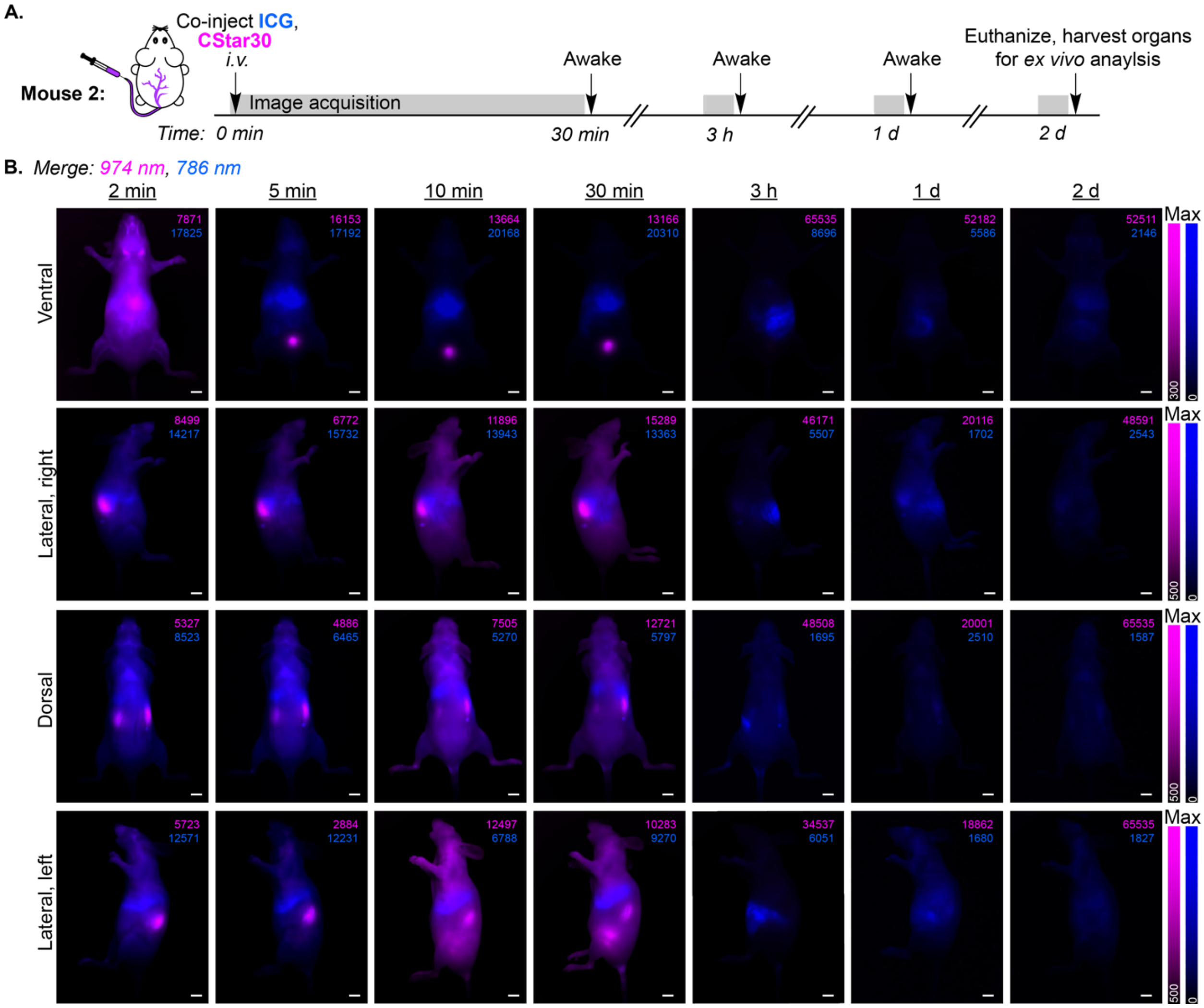

Figure S27: Two-color imaging timecourse of **5** (**CStar30**) and **ICG** co-injected *i.v.* in mice (replicate "Mouse 2"). (A) Injection scheme (200 µL, 30 nmol **CStar30** and 200 nmol **ICG** in sterile H_2O) and timeline. (B) Still images of the ventral, lateral (right, left) and dorsal views of the animal, timecourse from 2 min to 2 d post-injection, measured via InGaAs camera (786 nm and 974 nm ex, 100-160 mW/cm^2, 1100 nm LP, 33–100 fps). See Table S3 for sensitivity related parameters. Max intensity: upper right of each image. Scale bars: 10 mm.

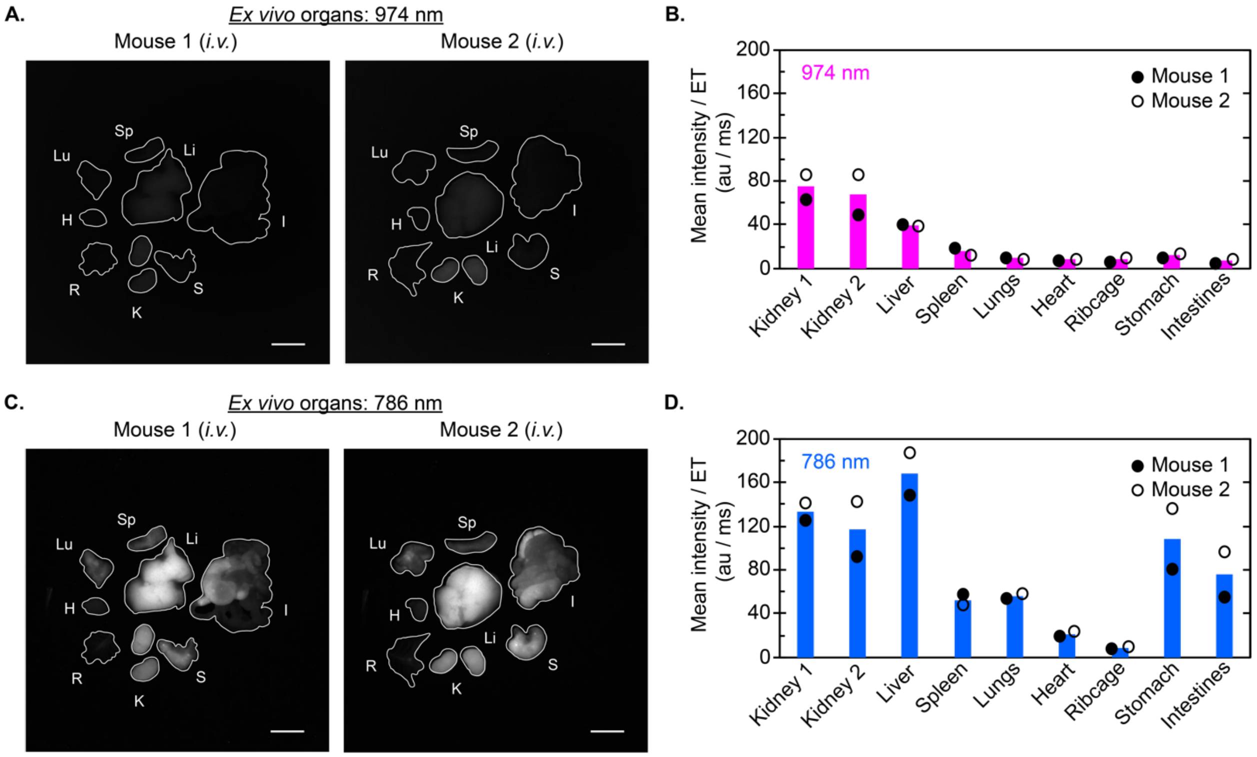

Figure S28: *Ex vivo* organs from two-color imaging of **5** (**CStar30**) at 974 nm and **ICG** at 786 nm injected *i.v.*, “Mouse 1” and “Mouse 2” after 2 d post-injection. (A) Fluorescence of resected organs, measured via InGaAs camera (974 nm ex, 160 mW/cm^2, 15 ms ET, 1100 nm LP). (B) Quantification of (A) for individual organs, normalized by ET. (C) Fluorescence of resected organs, measured via InGaAs camera (786 nm ex, 100 mW/cm^2, 20 ms ET, 1100 nm LP). (D) Quantification of (C) for individual organs, normalized by ET. Scale bars: 10 mm. *Organ key: H, heart; I, intestines; K, kidney; Li, liver; Lu, lungs; R, ribcage; S, stomach, Sp, spleen.*

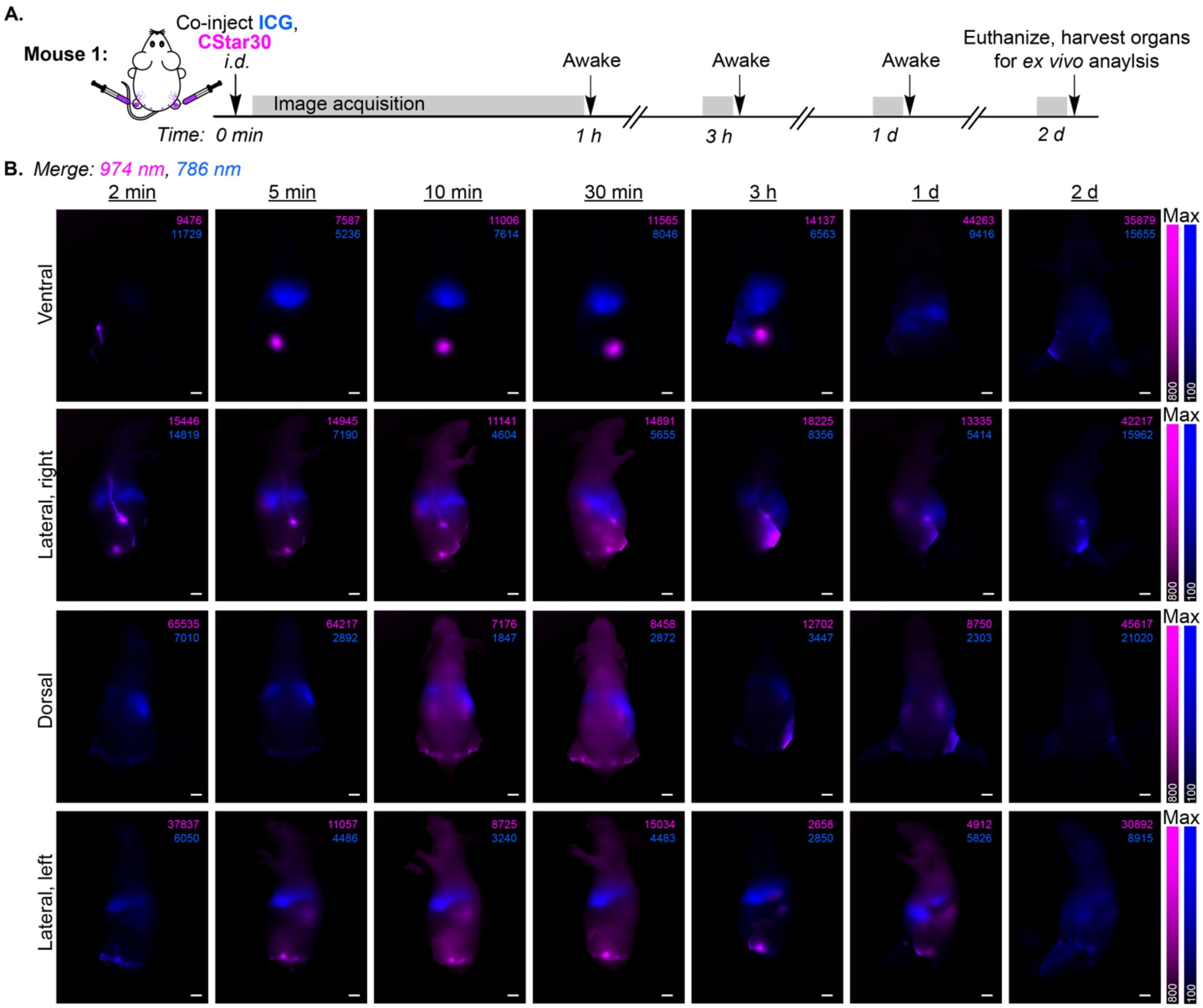

Figure S29: Two-color imaging timecourse of **5** (**CStar30**) and **ICG** co-injected *i.d.* in mice (replicate "Mouse 1"). (A) Injection scheme (50 µL per hind footpad, 30 nmol **CStar30** and 200 nmol **ICG** total in sterile H_2O) and timeline. (B) Still images of the ventral, lateral (right, left) and dorsal views of the animal, timecourse from 2 min to 2 d post-injection, measured via InGaAs camera (786 nm and 974 nm ex, 50-160 mW/cm^2, 1100 nm LP, 17–100 fps). See Table S3 for sensitivity related parameters. Max intensity: upper right of each image. Scale bars: 10 mm.

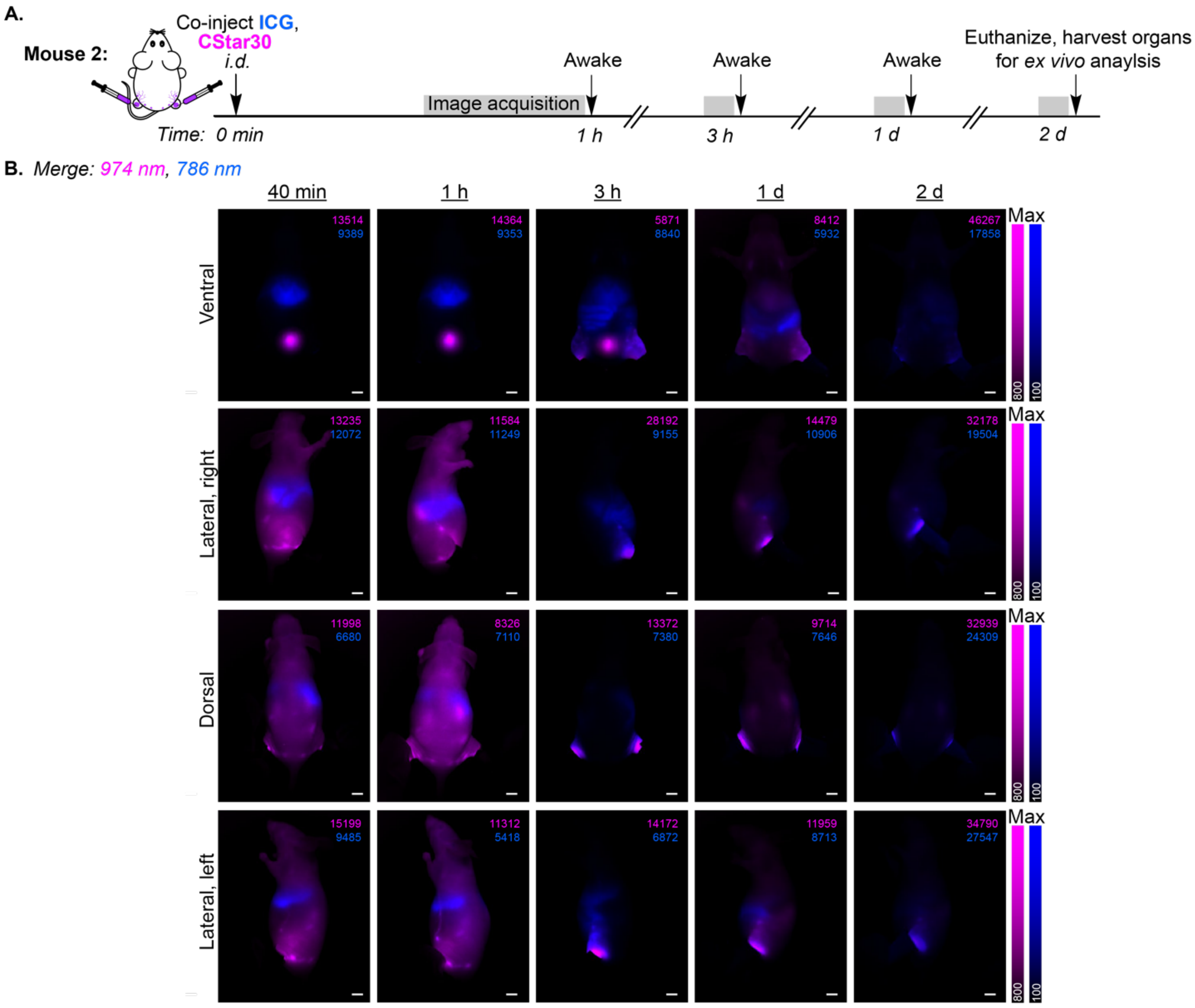

Figure S30: Two-color imaging timecourse of **5** (**CStar30**) and **ICG** co-injected *i.d.* in mice (replicate "Mouse 1"). (A) Injection scheme (50 µL per hind footpad, 30 nmol **CStar30** and 200 nmol **ICG** total in sterile H_2O) and timeline. (B) Still images of the ventral, lateral (right, left) and dorsal views of the animal, timecourse from 40 min to 2 d post-injection, measured via InGaAs camera (786 nm and 974 nm ex, 50-160 mW/cm^2, 1100 nm LP, 17–100 fps). See Table S3 for sensitivity related parameters. Max intensity: upper right of each image. Scale bars: 10 mm. *Note*: Although images were also recorded from 2 min to 30 min post-injection, deinterleaving of single excitation channels was not possible due to computer interface error. Data shown above are consistent with "Mouse 1" (see Figure S29), and therefore another replicate was not performed to minimize the use of animals.

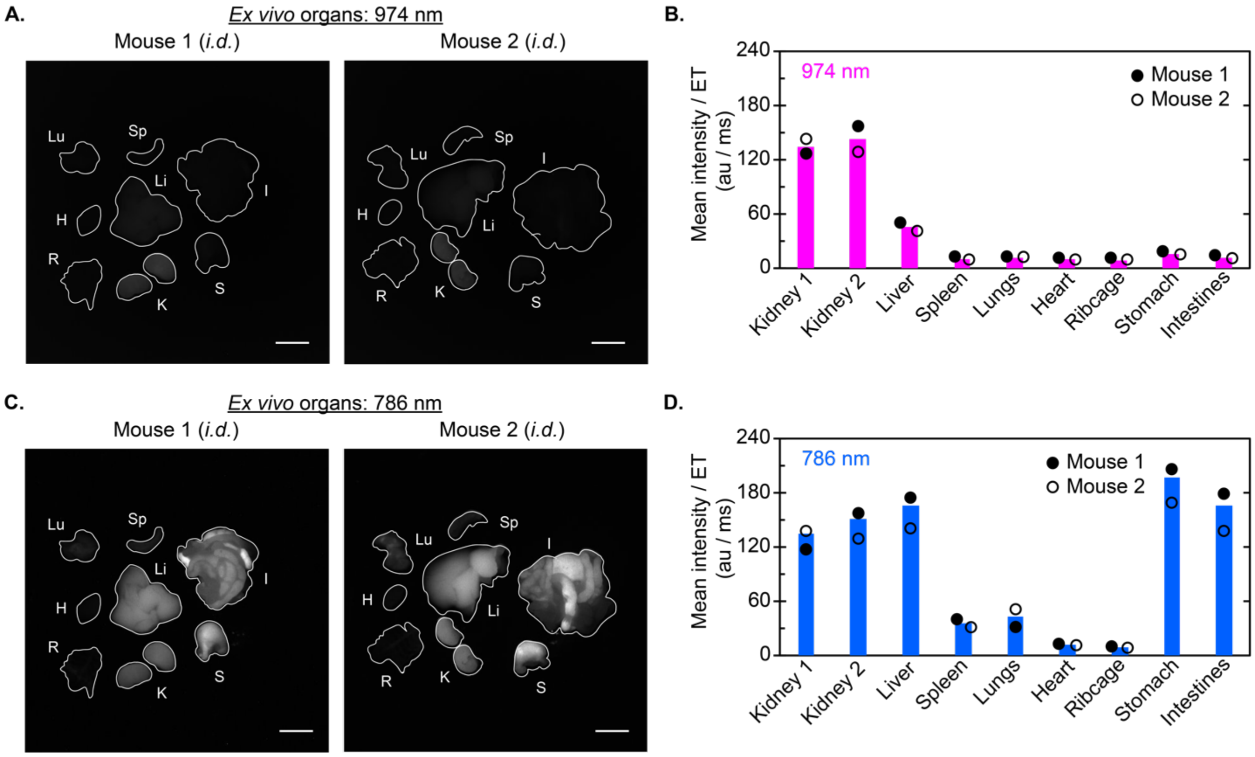

Figure S31: *Ex vivo* organs from two-color imaging of **5** (**CStar30**) at 974 nm and **ICG** at 786 nm injected *i.d.*, "Mouse 1" and "Mouse 2" after 2 d post-injection. (A) Fluorescence of resected organs, measured via InGaAs camera (974 nm ex, 160 mW/cm^2, 15 ms ET, 1100 nm LP). (B) Quantification of (A) for individual organs, normalized by ET. (C) Fluorescence of resected organs, measured via InGaAs camera (786 nm ex, 100 mW/cm^2, 20 ms ET, 1100 nm LP). (D) Quantification of (C) for individual organs, normalized by ET. Scale bars: 10 mm. *Organ key: H, heart; I, intestines; K, kidney; Li, liver; Lu, lungs; R, ribcage; S, stomach, Sp, spleen.*

II. Supporting Schemes

Note: Yields are reported for novel procedures. See "Synthetic Materials" for previously reported procedures, performed without further modification.

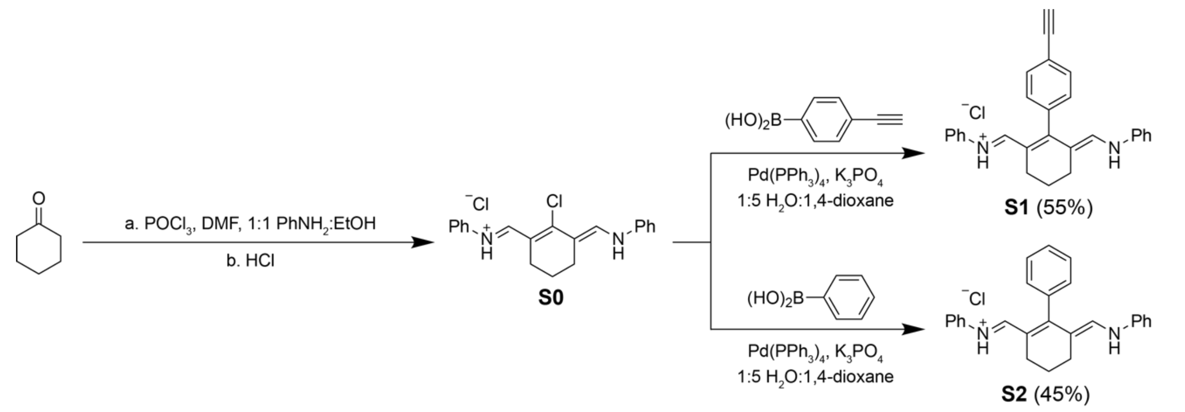

Scheme S1: Synthesis of heptamethine linkers **S1** and **S2**.

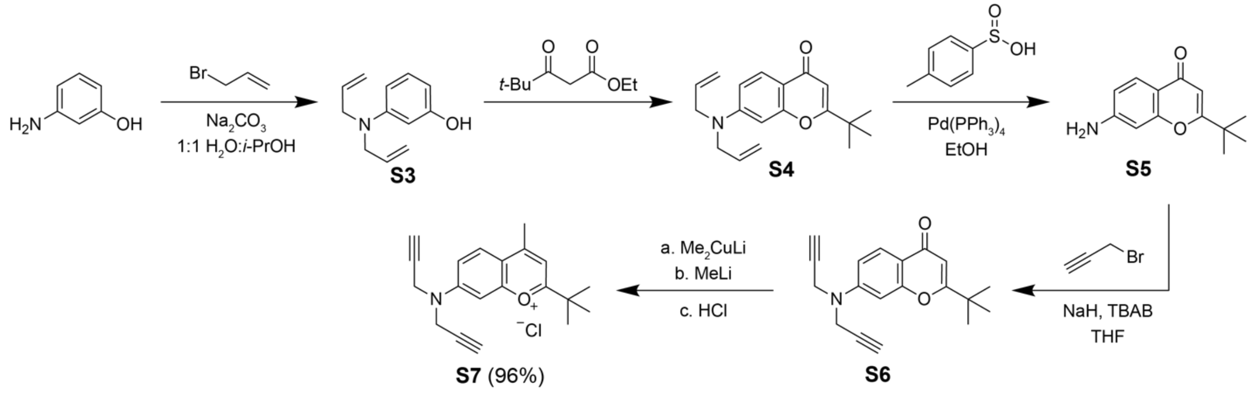

Scheme S2: Synthesis of chromenylium heterocycle **S7**.

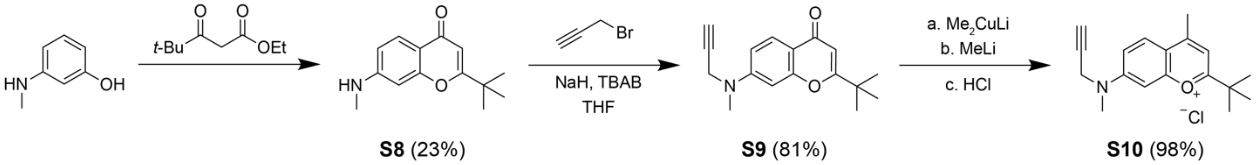

Scheme S3: Synthesis of chromenylium heterocycle **S10**.

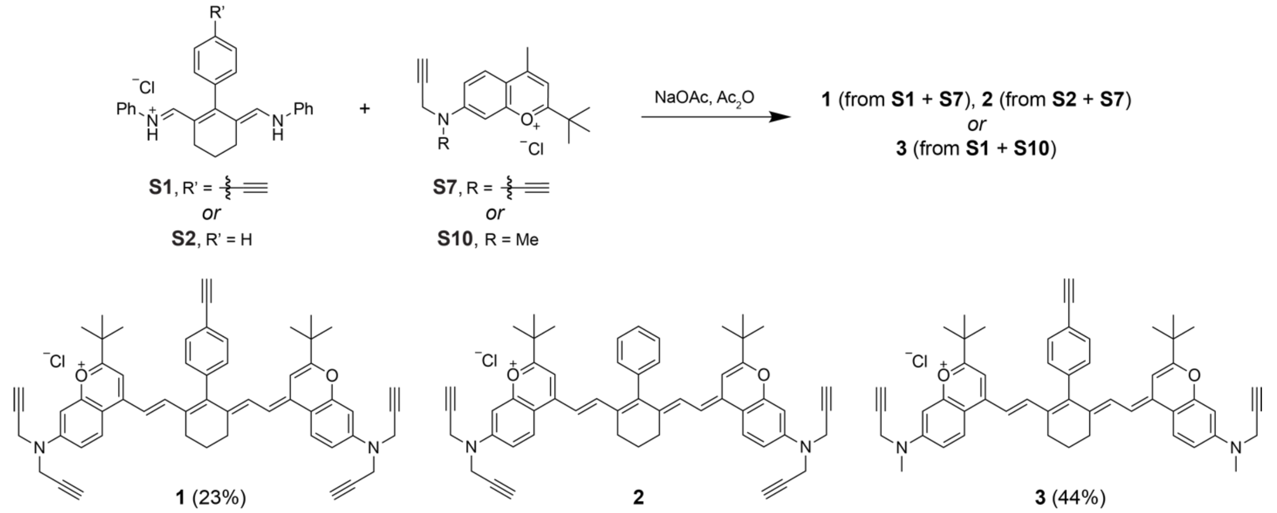

Scheme S4: Synthesis of heptamethine chromenylium core fluorophores **1**, **2**, and **3**.

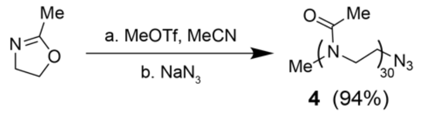

Scheme S5: Polymerization to access polymer **4**.

III. Supporting Tables

Table S1: Photophysical properties of all fluorophores in this work, with error.

Fluorophore	Solvent	$\lambda_{max, abs}$ (nm)	ε_{max} ($M^{-1}cm^{-1}$)	$\lambda_{max, em}$ (nm)	Φ_F (%)	Brightness ($M^{-1}cm^{-1}$)[a]
**1**	DCM	937	195,000 ± 7,000	952	2.26 ± 0.09	4,200 ± 200
	MeOH	927	132,000 ± 7,000	943	0.87 ± 0.02	1,150 ± 70
**5** (**CStar30**)	MeOH	939	128,000 ± 3,000	958	0.89 ± 0.03	1,140 ± 50
	H_2O	940	93,000 ± 3,000	953	0.63 ± 0.02	590 ± 30
	FBS	940	79,000 ± 3,000	954	0.58 ± 0.02	460 ± 20
**2**[b]	DCM	934	125,000 ± 3,000	953	1.72 ± 0.08	2,200 ± 100
	MeOH	924	108,000 ± 2,000	946	0.57 ± 0.05	620 ± 60
**6**	MeOH	938	136,000 ± 4,000	953	0.98 ± 0.07	1,300 ± 100
	H_2O	939	71,000 ± 2,000	951	0.50 ± 0.03	350 ± 20
**3**	DCM	944	174,000 ± 4,000	962	1.58 ± 0.09	2,800 ± 200
	MeOH	933	129,000 ± 3,000	957	0.90 ± 0.05	1,160 ± 70
**7**	MeOH	941	117,000 ± 4,000	965	1.00 ± 0.07	1,200 ± 800
	H_2O	945	n.d.[c]	961	n.d.[c]	n.d.[c]

Φ_F measured relative to Chrom7 in DCM[5]
a: Brightness = $\Phi_F \times \varepsilon_{max}$
b: Previously reported values in MeOH[2], Φ_F measured relative to Flav7 in DCM[4]
c: Not determined (n.d.) due to strong aggregation

Table S2: Sensitivity related parameters for single color imaging of **CStar30** (*in vivo* still images).[a]

Figure	Time[b]	Position	ET (ms)[c]	Figure	Time[b]	Position	ET (ms)[c]
5B	5 min	Ventral	10	S20A[d]	20 min	Lateral, left	20
		Lateral, Dorsal	10	S21B	2 min	Ventral	20
5D	20 min	Ventral	2			Lateral, Dorsal	10
		Lateral, Dorsal	20		10 min	Ventral	10
S15B	2 min	Ventral	15			Lateral, Dorsal	2
		Lateral, Dorsal	15		20 min	Ventral	20
	5 min	Ventral	10			Lateral, Dorsal	1
		Lateral, Dorsal	10		1 h	Ventral	10
	10 min	Ventral	5			Lateral, Dorsal	1
		Lateral, Dorsal	20		3 h	Ventral	10
	30 min	Ventral	2			Lateral, Dorsal	20
		Lateral, Dorsal	15		1 d	Ventral	20
	3 h	Ventral	10			Lateral, Dorsal	20
		Lateral, Dorsal	30		2 d	Ventral	20
	1 d	Ventral	40			Lateral, Dorsal	20
		Lateral, Dorsal	40	S22B	5 min	Ventral	30
	2 d	Ventral	50			Lateral, Dorsal	30
		Lateral, Dorsal	50		10 min	Ventral	30
S16B	2 min	Ventral	5			Lateral, Dorsal	10
		Lateral, Dorsal	5		10 min	Ventral	20
	5 min	Ventral	5			Lateral, Dorsal	3
		Lateral, Dorsal	5		30 min	Ventral	20
	10 min	Ventral	1			Lateral, Dorsal	10
		Lateral, Dorsal	5		3 h	Ventral	30
	30 min	Ventral	1			Lateral, Dorsal	30
		Lateral, Dorsal	5		1 d	Ventral	30
	3 h	Ventral	5			Lateral, Dorsal	30
		Lateral, Dorsal	20		2 d	Ventral	30
	1 d	Ventral	20			Lateral, Dorsal	20
		Lateral, Dorsal	20				
	2 d	Ventral	30				
		Lateral, Dorsal	30				
S17A	15 min	Ventral	1				
		Lateral, Dorsal	5				

a: All images recorded via InGaAs camera used a 1100 nm LP filter, 974 nm excitation (160 mW/cm^2) and are background subtracted with 200 frames with 0 mW/cm^2 irradiance. Raw brightness values associated with each image are measured from 100 averaged frames at the 16-bit depth and linearly projected to a 0-255 range for display with the grey LUT color scale.
b: Time post-injection of fluorophore
c: ET = exposure time
d: 1 single frame for each image

Table S3: Sensitivity related parameters for two-color excitation-multiplexed imaging of **CStar30** and **ICG** (*in vivo* still images).[a]

Figure	Time[b]	Position	Excitation (nm)	Irradiance (mW/cm^2)	ET (ms)[c]
6E[e]	2 min	Lateral, left	974 / 786	160 /100	8 / 1
	5 min	Lateral, left	974 / 786	160 / 100	5 / 1
	10 min	Lateral, left	974 / 786	160 / 100	10 / 1
	30 min	Lateral, left	974 / 786	160 / 100	15 / 1
	2 d	Lateral, left	974 / 786	160 / 100	20 / 50
6F[e]	2 min	Lateral, right	974 / 786	160 /100	20 / 5
	5 min	Lateral, right	974 / 786	160 / 100	20 / 1
	10 min	Lateral, right	974 / 786	160 / 50	20 / 1
	30 min	Lateral, right	974 / 786	160 / 50	20 / 1
	2 d	Lateral, right	974 / 786	160 / 100	20 / 5
S26B	2 min	Ventral	974 / 786	160 / 100	15 / 1
		Lateral, Dorsal	974 / 786	160 / 100	8 / 1
	5 min	Ventral	974 / 786	160 / 100	2 / 1
		Lateral, Dorsal	974 / 786	160 / 100	5 / 1
	10 min	Ventral	974 / 786	160 / 100	1 / 1
		Lateral, Dorsal	974 / 786	160 / 100	10 / 1
	30 min	Ventral	974 / 786	160 / 100	1 / 1
		Lateral, Dorsal	974 / 786	160 / 100	15 / 1
	3 h	Ventral	974 / 786	160 / 100	5 / 1
		Lateral, Dorsal	974 / 786	160 / 100	10 / 1
	1 d	Ventral	974 / 786	160 / 100	20 / 5
		Lateral, Dorsal	974 / 786	160 / 100	20 / 5
	2 d	Ventral	974 / 786	160 / 100	20 / 50
		Lateral, Dorsal	974 / 786	160 / 100	20 / 50
S27B	2 min	Ventral	974 / 786	160 / 100	15 / 1
		Lateral, Dorsal	974 / 786	160 / 100	5 / 1
	5 min	Ventral	974 / 786	160 / 100	2 / 1
		Lateral, Dorsal	974 / 786	160 / 100	5 / 1
	10 min	Ventral	974 / 786	160 / 100	1 / 1
		Lateral, Dorsal	974 / 786	160 / 100	15 / 1
	30 min	Ventral	974 / 786	160 / 100	1 / 1
		Lateral, Dorsal	974 / 786	160 / 100	20 / 1
	3 h	Ventral	974 / 786	160 / 100	20 / 1
		Lateral, Dorsal	974 / 786	160 / 100	20 / 1
	1 d	Ventral	974 / 786	160 / 100	20 / 10
		Lateral, Dorsal	974 / 786	160 / 100	20 / 10
	2 d	Ventral	974 / 786	160 / 100	20 / 10
		Lateral, Dorsal	974 / 786	160 / 100	20 / 10
S29B	2 min	Ventral	974 / 786	160 / 100	20 / 5
		Lateral, Dorsal	974 / 786	160 / 100	20 / 5
	5 min	Ventral	974 / 786	160 / 50	1 / 1
		Lateral, Dorsal	974 / 786	160 / 100	20 / 1
	10 min	Ventral	974 / 786	160 / 50	1 / 1
		Lateral, Dorsal	974 / 786	160 / 50	20 / 1
	30 min	Ventral	974 / 786	160 / 50	1 / 1
		Lateral, Dorsal	974 / 786	160 / 50	20 / 1
	3 h	Ventral	974 / 786	160 / 50	2 / 1
		Lateral, Dorsal	974 / 786	160 / 50	5 / 1
	1 d	Ventral	974 / 786	160 / 100	20 / 5
		Lateral, Dorsal	974 / 786	160 / 100	20 / 5
	2 d	Ventral	974 / 786	160 / 100	20 / 40
		Lateral, Dorsal	974 / 786	160 / 100	20 / 40
S30B	40 min	Ventral	974 / 786	160 / 50	1 / 1
		Lateral, Dorsal	974 / 786	160 / 100	20 / 1
	1 h	Ventral	974 / 786	160 / 50	1 / 1
		Lateral, Dorsal	974 / 786	160 / 100	20 / 1
	3 h	Ventral	974 / 786	160 / 50	2 / 1
		Lateral, Dorsal	974 / 786	160 / 100	2 / 1
	1 d	Ventral	974 / 786	160 / 100	20 / 40
		Lateral, Dorsal	974 / 786	160 / 100	20 / 40
	2 d	Ventral	974 / 786	160 / 100	20 / 40
		Lateral, Dorsal	974 / 786	160 / 100	20 / 40

a: All images recorded via InGaAs camera used a 1100 nm LP filter and are background subtracted with 200 frames with 0 mW/cm^2 irradiance. Raw brightness values associated with each image are measured from 100 averaged frames at the 16-bit depth and linearly projected to a 0-255 range for display with either the magenta or blue LUT color scale. See Figure SA1-SA8 for corresponding single channel images.
b: Time post-injection of fluorophore
c: ET = exposure time

IV. List of Supporting Videos

Video S1: Single channel injection of **CStar30** (*i.v.*) in mice.
The animal was positioned such that the dorsal side was facing the camera. **CStar30** was injected through the tail vein and video was recorded at 100 fps (974 nm ex, 160 mW/cm^2, 10 ms ET, 1100 nm LP) until the fluorophore began accumulating strongly in the kidneys. Kidney accumulation began immediately following clearance from the vasculature of the animal, and was prominent within 51 s. By 1 min 20 s, kidney accumulation predominated as the fluorophore continued clearing from the vasculature. Video corresponds to injection of "Mouse 2" (see Figure S16).

Video S2: Single channel *ex vivo* image-guided lymph node resection.
CStar30 was injected *i.d.* (50 µL per hind footpad, 30 nmol total in sterile water) and imaged until fluorophore began clearing lymphatic vessels and passing through the renal system (5 min). After 5 min, the animal was euthanized, and skin and organs were removed to visualize lymph nodes (eliminating contribution of tissue scattering and depth). The animal was positioned such that the ventral side was facing the camera, and image-guided resection of lymph nodes was performed in real time using an InGaAs camera at 100 fps (974 nm ex, 160 mW/cm^2, 3 ms ET, 1100 nm LP). Image-guided resection allows for increased contrast, which can enable surgeons to selectively identify and remove tissue. Video corresponds to the animal from Figure S19.

Video S3: Single channel lymphatic vessel velocity of **CStar30** in mice.
CStar30 was injected *i.d.* and the animal was imaged at the given timepoints as the fluorophore traversed lymphatic vessels, was reintroduced to vasculature, and passed through the renal system (kidneys to bladder). At 20 min post-injection, fluorophore can easily be visualized traversing lymphatic vessels with the lateral left view of the animal facing the camera at 50 fps (974 nm ex, 160 mW/cm^2, 20 ms ET, 1100 nm LP). Video corresponds to injection of "Mouse 1" at the 20 min timepoint (see Figure 5D, S20, S21).

V. General Experimental Procedures and Materials

No unexpected or unusually high safety hazards were encountered in the following procedures, unless stated otherwise.

General Abbreviations

BSA: bovine serum albumin; Chrom: chromenylium; cm: centimeter; CStar: chromenylium star; d: days; em: emission; DIPEA: *N,N*-diisopropylethylamine; ET: exposure time; ex: excitation; FBS: fetal bovine serum; fps: frames per second; GPC: gel permeation chromatography; GSH: L-glutathione; h: hours; H: heat; I: intestines; *i.d.*: intradermal; *i.v.*: intravenous; ICG: indocyanine green; InGaAs: indium gallium arsenide alloy; K: kidney; Li: liver; LN: lymph node; LP: longpass filter; Lu: lungs; LUT: look up table; MALDI-TOF/TOF: matrix-assisted laser desorption ionization tandem time of flight mass spectrometry; min: minutes; mm: millimeter; ms: milliseconds; mW/cm^2: milliWatts per centimeter squared; nm: nanometer; PAGE: poly(acrylamide) gel electrophoresis; POx: poly(2-methyl-2-oxazoline); R: ribcage; ROI: region of interest; s: seconds; S: stomach; SDS: sodium dodecyl sulfate; Sp: spleen; SP: shortpass filter; SWIR: shortwave infrared.

General Materials Handling and Storage

Unless otherwise noted, all synthesized materials were stored dry, under atmosphere in a -20 ˚C freezer. Solutions were made fresh and used within a few hours. All commercially available materials were stored according to manufacturing recommendations.

CStar GPC Calibration

Star polymers are known to have lower solution viscosities than linear polymers of the same size, often resulting in artificially low sizes measured via GPC, which obscures direct quantitative analysis using common linear polymer calibration curves.[6] Size determination by ^1H NMR is also complicated by intense shielding and large molecular weights.[6] Although MALDI TOF-TOF measurements support the expected size of CStars **5–7** was achieved, we also performed a GPC calibration to further corroborate these data, as well as reproducibility of the synthetic methods to yield well-defined CStars. Thus, CStars **5** (**CStar30**), **6**, and **7** were each synthesized according to "General Procedure for CuAAC" (see "Synthetic Procedures"), with the modification of just 1 d dialysis (1 kDa MWCO) against H_2O to remove only small molecule reaction materials. The solution was then lyophilized to dryness and analyzed by GPC (see "Synthetic Instrumentation"). The 550 nm channel signal was used to assess reaction conversion via visibility of other fluorophore containing intermediates. Four replicate syntheses were performed for each **5** (**CStar30**), **6**, and **7**, and the mean and standard deviation of each size metric (M_n, M_w, and M_p) were determined. One-way ANOVA and unpaired two-tailed t-tests were performed across all size metrics and CStars. This calibration was also replicated for all CStars with a higher dispersity ($Đ$) version of polymer **4** (synthesized following the procedure outlined in "Synthetic Procedures", with the modification of 4 M concentration).

CStar **5–7** GPC analyses are reported in Figure S6. The replicate experiment is reported in Figure S7.

Photophysical Instrumentation

Absorption spectra were collected on a JASCO V-770 UV–visible/NIR spectrophotometer after blanking with the appropriate solvent. Unless otherwise noted, absorbance spectra were collected with a scan range of 350–1300 nm, step size of 1 nm, and a scan rate of 1000 nm/min. Photoluminescence spectra were obtained on a Horiba Instruments PTI Quanta Master Series fluorometer with a liquid nitrogen cooled InGaAs detector (Horiba Edison DSS IGA 020L). Unless otherwise noted, photoluminescence spectra were collected with excitation slit widths of 15 nm × 15 nm, emission slit widths of 30 nm × 30 nm, step size of 1 nm, and integration time of 0.1 s. Traces were acquired with the default excitation correction (bias = -1.046, to set the base signal to 0), and with emission correction LUT "Em2Corr_1250 nm grating_Sample Holder_quanta". For excitation and emission spectra, excitation/emission wavelength and collection ranges vary (described for each experiment). Quartz cuvettes (10 mm × 10 mm [Thorlabs or Starna Cells Inc.], or 3 mm × 3 mm [Starna Cells Inc.]) were used for both absorption and photoluminescence measurements. All spectra were obtained at ambient temperature.

Photophysical data are reported in Figures 3, 4, 6, S4, S8–S10, S12 and Table S1.

Determination of Fluorescence Quantum Yield

Relative photoluminescence quantum yield (Φ_F) of fluorophores in this work were determined in accordance with the reported procedure.[7] Briefly, Φ_F was determined for each unknown fluorophore with a known standard fluorophore that emits in the same region of the electromagnetic spectrum. Here, we used Chrom7 as a standard reference fluorophore (Φ_F = 1.70% in DCM).[4] A stock solution of each fluorophore was prepared, and serial dilutions were performed. Each fluorophore solution was diluted to have an optical density of less than or equal to 0.1 to minimize the effects of reabsorption. In total, five solutions of each fluorophore, varying in optical density, were prepared in the appropriate solvent and the absorbance and photoluminescence spectra were acquired (10 mm × 10 mm quartz cuvette (Starna Cells Inc.), 860 nm excitation, 950 nm SP filter (Thorlabs FES 950, see "Photophysical Instrumentation") for the excitation beam, emission collection 885-1500 nm). Photoluminescence spectra were baseline corrected (at 1500 nm) and the traces were integrated. Raw integrals of each trace were corrected by subtracting the integral (of the same range) from photoluminescence traces of the blank solvent. Absorbance spectra were baseline corrected (at 1300 nm) and the intensity at the excitation wavelength (860 nm) was recorded to generate a linear plot of integrated fluorescence intensity versus absorbance at the excitation wavelength for the reference and unknown fluorophores. The slope and error in the slope were obtained ($R^2 > 0.99$ for all traces), and the unknown Φ_F was determined by the following relationship:

$$\Phi_{F,x} = \Phi_{F,r}\,(m_x/m_r)(\eta_x^2/\,\eta_r^2) \qquad \text{(Eq. 1)}$$

where m represents the slope of the line ($y = mx + b$) of integrated fluorescence intensity versus absorbance at the excitation wavelength, η is the refractive index of the solvent at ambient temperature, and the subscripts x and r correspond to the values of the unknown and reference fluorophore, respectively. Error measurements were propagated from the error in slope of the reference and unknown fluorophore samples.

Calculated Φ_F for each fluorophore are reported in Figure 3D and Table S1.

Determination of Absorption Coefficient
The absorption coefficient at the maximum wavelength of absorption (ε_{max}) of fluorophores in this work was calculated according to Beer Lambert's law:

$$A = \varepsilon \text{ b c} \qquad \text{(Eq. 2)}$$

where A is the absorbance in O.D., b is the path length in cm, and c is the concentration in molarity (M). Briefly, for one ε determination, fluorophore was weighed on a Sartorius MSE6.6S-000-DM S13 Cubis Micro Balance. A stock solution was prepared with the appropriate solvent in a volumetric flask and capped to prevent evaporation. Serial dilutions into smaller volumetric flasks were performed with Hamilton syringes, and the absorbance was measured immediately (10 mm × 10 mm quartz cuvette [Thorlabs], or 3 mm × 3 mm quartz cuvette [Starna Cells Inc.], see "Photophysical Instrumentation"). A minimum of five concentrations were measured, with $R^2 \geq 0.99$. Absorption coefficient (ε_{max}) is reported as the mean of three independent determinations, with standard deviation as the error.

Calculated ε_{max} for each fluorophore are reported in Figure 3D and Table S1.

Determination of Copper Concentration
Inductively coupled plasma mass spectrometry (ICP-MS, NexION 2000, PerkinElmer) analysis was performed to detect copper (Cu) in ng/mg. All samples were weighed as a solid on a Sartorius MSE6.6S-000-DM S13 Cubis Micro Balance. Each sample was then transferred to a clean Teflon vessel for acid digestion. Digestion was carried out with a concentrated HNO_3 (65-70%, Trace Metal Grade, Fisher Scientific) with a supplement of H_2O_2 (30%, Certified ACS, Fisher Scientific) at room temperature for 2 h. Once the sample was completely digested, it was subsequently diluted to make a final volume of 5 mL by adding filtered DI water for analysis. The calibration curve was established using a standard solution while the dwell time was 50 ms with thirty sweeps and three replicates with background correction. Copper content is reported as the mean of three measurements, with error as the standard deviation.

The copper content of **5** (**CStar30**) was determined to be 11.3 ± 0.2 ng/mg.

SDS-PAGE Assay
All CStar **5–7** samples were prepared separately in milliQ H_2O and then mixed in the appropriate ratio with 4X Laemmli sample buffer with 10% 2-mercaptoethanol (Bio-Rad). CStars **5–7** were loaded into separate lanes at 0.2 nmol each on an 8-16% precast gel (Criterion, Bio-Rad). The gel was run at 150 V at 25 ˚C in SDS buffer (Bio-Rad) and imaged via a ChemiDoc gel imaging system (Coomassie blue) and InGaAs camera (see "SWIR Imaging Apparatus").

SDS-PAGE of CStars **5-7** are reported in Figure S5.

Native PAGE Assay
All fluorophore (**5** [**CStar30**] or **SulfoChrom7**) samples were prepared separately in milliQ H_2O and then mixed in 4X Native sample buffer (Thermo Fisher). For all protein-fluorophore samples, the fluorophore and protein-containing solutions (BSA, FBS, or *E. Coli* cell lysate) were each mixed and incubated for 1 hour at room temperature with gentle shaking before mixing with sample buffer in the appropriate ratio. Samples were then loaded into separate lanes at 0.6 nmol

fluorophore, with or without 5 µg BSA or 20 µg total protein (FBS or *E. Coli* lysate) on a 12% precast gel (Criterion, Bio-Rad). The gel was run at 150 V at 0 ˚C in Native running buffer (Thermo Fisher), destained, and then imaged via a ChemiDoc gel imaging system (Coomassie blue) and InGaAs camera (see "SWIR Imaging Apparatus").

Native PAGE of **CStar30** and **SulfoChrom7** with protein are reported in Figure S11.

Fluorophore Stability Assay

Fluorophores were diluted to an O.D. of approx. 0.60–0.75 in 2 mL of the appropriate solvent (FBS containing 0.02% w/v NaN_3, milliQ water, or milliQ water with 1 or 10 mM GSH). This stock solution was split into 600 µL aliquots, and each aliquot was sealed with a cap and parafilm in a dram vial. Vials were placed in a 37 ˚C incubator until the given time points. At each timepoint, absorption spectra were recorded (see "Photophysical Instrumentation"). The absorption intensity at the fluorophore's maximum absorbance wavelength was used to represent relative fluorophore concentration. Each condition was performed in triplicate.

Fluorophore stability data for **5** (**CStar30**) are reported in Figure S12.

Photostability Assay

Solutions of **5** (**CStar30**) were prepared in either FBS or milliQ water (15 µM). These solutions were placed in capillary tubes (Disposable Micro Capillary Pipets, 50 µL (DWK Life Sciences Kimble, Cat#71900-50)), sealed, and positioned onto a holder under an InGaAs camera and 974 nm laser (see "SWIR Imaging Apparatus"). Samples were irradiated at 974 nm (100 mW/cm^2) and emission intensity (1100 nm LP) was recorded at the given timepoints at 100 fps (5 ms ET), until an 80% decrease in emission intensity was observed (the same conditions reported for **SulfoChrom7**,[2] for comparison). Raw imaging files were processed according to "Image Processing" to generate emission intensity for each sample at the given timepoints. The % emission intensity remaining was plotted against time. Data from time points 1–10 min were fit to a mono-exponential decay and the rate of photobleaching (k_{raw}) was determined via the following relationship:

$$\ln[A] = -kt + \ln[A]_0 \qquad \text{(Eq. 3)}$$

where A and A_0 represent collection at time *t* and initial emission collected, respectively. All R^2 values ≥ 0.98. Photobleaching rate is reported as the mean of three replicate measurements, with standard deviation as the error.

Photobleaching data for **5** (**CStar30**) are reported in Figure 4F and S13. Figure 4F also includes photobleaching data for **SulfoChrom7**, replotted from our previous work.[2]

Cell Toxicity Assay

RAW 264.7 cells stock were purchased from ATCC (Catalog number: TIB-71) and cultured in complete Dulbecco's Modified Eagle Media (DMEM, Life Technologies, Catalog number: 11995073) supplemented with 10% FBS (Corning), sodium pyruvate (Thermo Fisher, Catalog number: 11360070) and 1% penicillin-streptomycin (Life Technologies, Catalog number: 15070063) at 37 ˚C and 5% CO_2 in Heracell 150i CO_2 incubators. For passaging, cells were trypsinized, scraped, and centrifuged using a Sorvall ST40R series centrifuge (526 g for 3 min). The pellet was resuspended in fresh media and plated onto a fresh tissue culture dish. For cell

toxicity, cells were plated at a cell density of 2×10^6 cells per well in 12-well plates. Media was removed, and fresh media containing **CStar30** (7.5-30 μM) or media containing 2% volume PBS (0 μM) was added. Cells were incubated for 4 h, after which media was removed. Cells were then trypsinized, treated with trypan blue, and counted using the Countess 3 Automated Cell Counter. Each concentration was performed in triplicate.

Cell toxicity data for **5** (**CStar30**) are reported in Figure S14.

Animal Procedures

Animal experiments were conducted in conformity with guidelines from the University of California, Los Angeles IACUC, with protocols approved by the Animal Research Committee (protocol number ARC-2018-047). Non-invasive, whole mouse imaging was performed on athymic female mice (NU/J, 6-16 weeks old), purchased from The Jackson Laboratories. Mice were anesthetized by inhalation of 2-4% isoflurane/oxygen and placed on a heating pad (to maintain body normal temperature under anesthesia) with a nose cone affixed to the imaging stage (see "SWIR Imaging Apparatus"). Artificial tears were added to each eye to prevent corneas from drying out during imaging experiments. For tail vein (*i.v.*) injections, a catheter was assembled from a 29-gauge needle (VetriJec™) connected through plastic tubing to a syringe (29 gauge, VetriJec™ insulin syringe) prefilled with sterile isotonic saline solution. The bevel of the needle was inserted into either of the lateral or dorsal tail veins and secured with a tissue adhesive. The catheter was then connected to a syringe (29-gauge, VetriJec™ insulin syringe) prefilled with the compound of interest in sterile water, followed by injection. After injection, the catheter was chased with sterile isotonic saline to ensure full dosage. The total injection volume for *i.v.* injections never exceeded 200 μL. For footpad (*i.d.*) injections, the bevel of a syringe (29-gauge, VetriJec™ insulin syringe) prefilled with the compound of interest in sterile water was inserted to the intradermal space in either hind footpad, followed by injection. The total injection volume for *i.d.* injections never exceeded 50 μL per footpad. Unless otherwise noted, hind feet are covered for *i.d.* injection images. All solutions were filtered through a 0.22 μm syringe filter prior to injection (*i.v.* and *i.d.*). Mice were briefly imaged (see "SWIR Imaging Apparatus") at given time points during and post-injection (defined for each experiment, with no more than 1 h at a time under anesthesia) for up to 2 d. Euthanasia was performed at the given time point while under anesthesia with inhalation of excess isoflurane followed by cervical dislocation. Individual organs were then excised for *ex vivo* fluorescence analysis.

Animal data are reported in Figures 5, 6, S15–23, S26–S31, Tables S2, S3, and Appendix A.

SWIR Imaging Apparatus

For whole mouse imaging (see "Animal Procedures"), a custom-built imaging setup was used. Lumics laser units: LU0975DLU350-S30AN03 (35 W) "975 nm"; LU0785D250-U70AN (25 W) "785 nm". *Note*: excitation wavelengths are reported as "974 nm" and "786 nm", respectively, according to maximum wavelength intensity via spectrometer measurement. Lasers were coupled in a 4-in-1 fan out fiber-optic bundle of 600 μm core diameter (Thorlabs BF46LS01) for each optical path. The fiber bundle output was fixed through an excitation cube (Thorlabs KCB1E), reflected off of a mirror (Thorlabs BBE1- E03), and passed through a positive achromat (Thorlabs AC254-050-AB-ML), SP filters (2x, High Performance OD 4.0 1100 nm SP, Edmund Optics #64-339) and an engineered 20° square diffuser (Thorlabs ED1-S20-MD) to provide uniform

illumination over the imaging stage. This working area was covered by a heating mat, nose cone (to deliver anesthesia to mice), and non-reflective blackout fabric (Thorlabs BK5). The excitation flux was measured over the illumination area with a digital optical power and energy meter (Thorlabs PM100D) in mW/cm^2, and adjusted in accordance with ICNIRP guidelines for whole mouse imaging.[8] An InGaAs camera (Allied Vision Goldeye G-032 Cool TEC2) camera was mounted vertically above the imaging stage to detect emitted light. The camera was fitted with a C-mount camera lens (Kowa LM35HC-SW) and a set of LP emission filters (filter set for "1100 nm LP": 2x High Performance OD 4.0 1100 nm LP, #84-768 Edmund Optics; 1x FELH1100, Thorlabs). The camera used a sensor temperature set point of -30 °C and gain = 1. Exposure time was defined for each experiment. The assembly was partially enclosed to avoid excess light while enabling manipulation of the field of view during operation. Both the camera and lasers were externally controlled and synchronized by delivering trigger pulses of 5V Transistor-Transistor Logic to the laser drivers and camera, using a programmable trigger controller with pulses generated with an Atmel Atmega328 micro-controller unit and programmed using Arduino Nano Rev 3 MCU (A000005) in the Arduino integrated development environment (IDE). Imaging data acquired by this system are then transferred to the PC via a Gigabit Ethernet (GigE), or CameraLink (CL) interface. For image acquisition, the toolbox of MATLAB programming environment was used in combination with a MATLAB script (CCDA V3, https://gitlab.com/brunslab/ccda) to preview and collect image data in 14-bit depth.

Data collected with the SWIR imaging apparatus are reported in Figure 3–6, S5, S11, S13, S15–23, S25–31, Tables S2, S3, and Appendix A. "Image Processing" procedures for data displayed is described below.

Image Processing

Raw image files (collected using the setup described in "SWIR Imaging Apparatus", above) were processed using the FIJI[9] distribution of ImageJ.[10] All images and videos were subtracted with a no-laser background (200 frames taken at the beginning of every imaging session) to correct for non-linearities in the detector and/or excitation. With background subtracted frames, frames of interest were averaged using the "Z-project" feature (100 frames, unless otherwise stated). Frames of interest were generally the dorsal, lateral (left and right), and ventral sides of the mice for whole live animal imaging, and still frames for capillaries, PAGE gels, and *ex vivo* organ images. Single channel images underwent no further processing. For excitation-multiplexed imaging, averaged frames of interest from the 974 nm and 786 nm excitation channels were merged via the "Merge channels" feature, with 974 nm assigned to the magenta LUT scale and 786 nm assigned to the blue LUT scale. As crosstalk between channels was estimated to be less than 10% from the capillary imaging experiment (Figure S22), no unmixing correction was used. Brightness values displayed with the appropriate LUT scale (grey, magenta, or blue) were obtained from the 14-bit images through the "Brightness/contrast..." window. All images were then compressed to the 8-bit depth for display.

For *in vivo* quantification, regions of interest were highlighted across relevant areas of the animal using the line ROI tool. For averaged frames, fluorescence intensity across the ROI was plotted using the "Plot profile" feature. For a set of frames, mean fluorescence intensity over time across the ROI was plotted using the "Plot Z-axis profile" feature.

For *ex vivo* organ quantification, regions of interest were highlighted around individual organs using the hand-drawn ROI tool. The mean intensities were calculated from the "Measure" function

and reported as the mean of three hand-drawn measurements, after subtracting background (measured within the illumination window in-between organs in the same manner). For each animal, mean fluorescence intensity values were normalized by dividing by the exposure time (ms) for comparison. Normalized fluorescence intensity values for replicate animals were then averaged to generate quantitative bar graphs.

For capillary quantification, regions of interest were highlighted via the line ROI tool, drawing a line horizontally across a set of capillary tubes. Fluorescence intensity across this ROI was plotted using the "Plot profile" feature.

For videos, frames of interest from background subtracted frames were selected using the "Slice keeper" tool. For the injection video (Video S1), frames of interest spanned from before dye was injected until the biodistribution was no longer actively changing post-injection (approximately). For the *ex vivo* lymph node dissection video (Video S2), frames of interest spanned from before, during, and after image-guided lymph node resection. For the lymphatic vessel video (Video S3), frames of interest spanned the fluorophore traversing the vessel. Quantification of lymphatic flow rate from this video is outlined in Figure S20. For all videos, the brightness/contrast was adjusted without pixel saturation via the last frame of interest, and all frames were then compressed to a .AVI file at the appropriate fps.

All raw and processed files can be found on BioImage Archive, accession number: S-BIAD1368.

VI. Synthetic Procedures and Characterization

No unexpected or unusually high safety hazards were encountered in the following procedures, unless stated otherwise.

Synthetic Abbreviations

Ac_2O: acetic anhydride; AcCl: acetyl chloride; CD_2Cl_2: deuterated dichloromethane; CD_3OD: deuterated methanol; $CDCl_3$: deuterated chloroform; $CHCl_3$: chloroform; CStar: chromenylium star; CuI: copper iodide; $CuSO_4$: copper sulfate; d: days; DCM: dichloromethane; DCTB: *trans*-2-[3-(4-*tert*-butylphenyl)-2-methyl-2-propenylidene]malononitrile; Dioxane: 1,4-dioxane; Et_2O: diethyl ether; EtOAc: ethyl acetate; EtOH: ethanol; GPC: gel permeation chromatography; h: hours; H_2O: deionized water; HCl: hydrochloric acid; HPLC: high performance liquid chromatography; HRMS: high resolution mass spectrometry; Hz: Hertz; *i*-PrOH: isopropylalcohol; ICP-MS: inductively coupled mass spectrometry; K_3PO_4: potassium phosphate; LCMS: liquid chromatography mass spectrometry; MALDI-TOF: matrix-assisted laser desorption ionization time of flight mass spectrometry; MALDI-TOF/TOF: matrix-assisted laser desorption ionization tandem time of flight mass spectrometry; MeCN: acetonitrile; MeLi: methyl lithium; MeOH: methanol; MeOTf: methyl triflate; MeOTs: methyl tosylate; MHz: Mega Hertz; min: minutes; MWCO: molecular weight cut-off; N_2: dinitrogen gas; Na_2EDTA: sodium ethylenediaminetetraacetic acid salt; $Na_2S_2O_3$: sodium thiosulfate, Na_2SO_4: sodium sulfate; NaAsc: sodium ascorbate; NaH: sodium hydride; $NaHCO_3$: sodium bicarbonate; NaOAc: sodium acetate; NaTFA: sodium trifluoroacetate; NMR: nuclear magnetic resonance; $Pd(PPh_3)_4$: palladium-tetrakis(triphenylphosphine); POx: poly(2-methyl-2-oxazoline); Silica: silicon dioxide; TBAB: tetrabutylammonium bromide; TFA: trifluoroacetic acid; TFE: trifluoroethanol; THF: tetrahydrofuran; THPTA: tris(benzyltriazolylmethyl)amine; TLC: thin-layer chromatography, α-cin: α-cyano-4-hydroxycinnamic acid.

Synthetic Materials

Unless otherwise noted, all commercial reagents were used directly without further purification. All reactions utilizing air- or moisture-sensitive reagents were performed in dried glassware under an atmosphere of dry N_2. Chemical reagents were purchased from Sigma-Aldrich, Fisher Scientific, TCI America, CombiBlocks, Cytiva, AK Scientific, VWR, and Acros Organics. Dialysis membranes were purchased from VWR (Spectra/Por®6 Dialysis Membrane, Pre-wetted RC tubing). Anhydrous solvents were dispensed from a Grubb's-type Phoenix Solvent Drying System constructed by the late JC Meyer or kept dry under sieves and N_2 in a Schlenk bomb. Compounds **S0**;[11] **3**, **S3**, **S4**, **S5** and **S6**[2] were synthesized based on published procedures, with no modifications. Synthesis of compounds **4**;[12] **S2** and **S7**[2] were adapted from published procedures, with modifications to arrive at higher yields and/or purity of isolated materials (see "Synthetic procedures").

Synthetic Instrumentation

Microwave reactions were performed using a CEM Discover SP microwave synthesis reactor. All reactions were performed in glass microwave reactor vials purchased from CEM with silicone/PTFE caps. TLC was performed using Silica Gel 60 F254 (EMD Millipore) plates and visualized with UV or ambient light. Flash column chromatography was executed with technical

grade silica gel with 60 Å pores and 40–63 μm mesh particle size (Sorbtech Technologies). All aqueous size exclusion chromatography was performed with Sephadex G50 resin (superfine), hand-packed into a 12 mL syringe column and eluted with gravity. GPC was performed with a 20 mM NaTFA in TFE mobile phase, at 25 ˚C (ambient temperature) with a 0.5 mL/min flow rate on a PU-4180 LC pump system equipped with two 300 mm PFG analytical linear S columns with a 5 μm stationary phase pore size (PSS Agilent) and tandem UV-4075 and RI-4030 detectors (JASCO). All GPC samples were prepared at ~0.5 mg/mL in the mobile phase and filtered through a 0.22 μm syringe filter. POx, synthesized in-house (see "Appendix B") were used as standards for calibration. Reverse phase preparatory HPLC separation was carried out on a PU-4087 LC pump system equipped with a Zorbax SB-C18 column and UV-4070 detector (JASCO). NMR (^{1}H NMR, ^{13}C NMR) spectra were taken on a Bruker DRX500, AV500, or NEO600 (500 or 600 MHz) spectrometer and processed with MestReNova software. All chemical shifts in ^{1}H NMR and ^{13}C NMR spectra are reported in the standard notation, with ppm relative to residual solvent signals ($CDCl_3$ δH=7.26, δC=77.16; CD_3OD δH=3.31, δC=49.00; CD_2Cl_2 δH=5.32, δC=53.84). Multiplicities are as indicated: s (singlet), d (doublet), dd (doublet of doublets), t (triplet), q (quartet), and m (multiplet). Coupling constants, *J*, are reported in Hz and integration is provided. HRMS was acquired on a 6545 LC/Q-ToF LCMS (ESI$^+$, Agilent), Ultraflex MALDI-TOF (Bruker), or MALDI-TOF/TOF (AB Sciex MALDI 5800). Unless otherwise noted, all LCMS samples were prepared at ~1 mg/mL in 40% MeCN in H_2O. For polymer **4**, MALDI-TOF samples were prepared in 50:5:1 mixtures of 20 mg/mL DCTB in $CHCl_3$, 20 mg/mL polymer in $CHCl_3$, and 10 mg/mL NaTFA in *i*-PrOH, respectively. For CStars **5**–**7**, MALDI-TOF/TOF samples were prepared in 1:1 mixtures of 20 mg/mL α-cin in 1:1 MeCN:0.1% TFA in H_2O, and ~1 mg/mL CStar in H_2O. Masses for analytical measurements were taken on a Sartorius MSE6.6S-000-DM S13 Cubis Micro Balance. Organic solvent was removed with a Büchi or Ika Rotovapor equipped with a Welch self-cleaning dry vacuum pump, and further dried with a Welch DuoSeal vacuum pump attached to a Schlenk manifold. Water was removed by lyophilization of flash-frozen solutions with a LABCONCO FreeZone Benchtop Freeze Dryer.

Synthetic Procedures

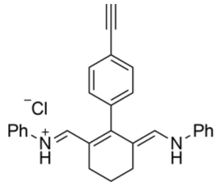

S1

N-((*E*)-((*E*)-4'-ethynyl-6-((phenylamino)methylene)-3,4,5,6-tetrahydro-[1,1'-biphenyl]-2-yl)methylene)benzenaminium chloride (**S1**): Compound **S0** (400 mg, 1.11 mmol, 1.00 equiv.), (4-ethynylphenyl)boronic acid (195 mg, 1.33 mmol, 1.20 equiv.), $Pd(PPh_3)_4$ (64 mg, 0.056 mmol, 0.050 equiv.), and K_3PO_4 (707 mg, 3.33 mmol, 3.00 equiv.) were dissolved in a 1:5 H_2O/Dioxane mixture (30 mL) followed by three freeze-pump-thaw cycles to fill in N_2. The reaction mixture was stirred at 80 ˚C for 14 h. The reaction mixture was then cooled to 25 ˚C and diluted in H_2O (150 mL). The solution was extracted with DCM (3x50 mL). The organic phase was dried (Na_2SO_4) and concentrated. The crude product was evaporated onto silica and purified by column chromatography (gradient 1:50 to 1:20 to 1:15 acetone/toluene) and acidified by dry HCl in MeOH (prepared by dissolving 0.5 mL of AcCl in 5 mL of cold, anhydrous MeOH) to give **S1** as a dark

red, iridescent solid (260 mg, 0.612 mmol, 55%) that was used without further purification. R_f in 1:5 acetone/toluene: 0.4. ^{1}H NMR (600 MHz, CD_3OD) δ 7.87 (d, *J* = 8.8 Hz, 1H), 7.67 (d, *J* = 7.7 Hz, 2H), 7.41 (d, *J* = 8.1 Hz, 2H), 7.35 (t, *J* = 7.9 Hz, 4H), 7.19 (t, *J* = 7.4 Hz, 2H), 7.07 (d, *J* = 7.6 Hz, 4H), 6.83 (d, *J* = 8.8 Hz, 1H), 3.72 (s, 1H), 2.73 (t, *J* = 6.1 Hz, 4H), 2.08 (t, *J* = 6.3 Hz, 2H). ^{13}C NMR (151 MHz, CD_3OD) δ 169.2, 150.9, 139.3, 135.8, 131.7, 130.7, 129.6, 126.0, 118.7, 117.9, 114.8, 82.1, 79.5, 22.8, 20.0. HRMS (ESI$^+$) calcd 389.2012, found 389.2023 for $C_{28}H_{25}N_2$ ([M$^+$]). *Note*: Heptamethine linker modifications are synthetically challenging to isolate in the aniline form due to hydrolysis and/or acetylation. However, both side products are synthetically competent for condensation with heterocycle, and are present in ca. < 5% in the final material.

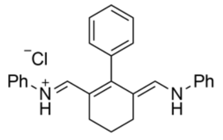

S2

N-((*E*)-((*E*)-6-((phenylamino)methylene)-3,4,5,6-tetrahydro-[1,1'-biphenyl]-2-yl)methylene)benzenaminium chloride (**S2**): This synthesis has been previously reported.[2] Altering flash chromatography conditions resulted in a higher isolated yield, with comparable purity. Compound **S0** (400 mg, 1.11 mmol, 1.00 equiv.), phenylboronic acid (163 mg, 1.34 mmol, 1.20 equiv.), $Pd(PPh_3)_4$ (64 mg, 0.056 mmol, 0.050 equiv.), and K_3PO_4 (707 mg, 3.33 mmol, 3.00 equiv.) were dissolved in a 1:5 H_2O/Dioxane mixture (30 mL) followed by three freeze-pump-thaw cycles to fill in N_2. The reaction mixture was stirred at 80 ˚C for 14 h. The reaction mixture was cooled to 25 ˚C and diluted in H_2O (150 mL). The solution was extracted with DCM (3×50 mL). The organic phase was dried (Na_2SO_4) and concentrated. The crude product was evaporated onto silica and purified by column chromatography (gradient 1:50 to 1:20 to 1:15 acetone/toluene) and acidified by dry HCl in MeOH (prepared by dissolving 0.5 mL of AcCl in 5 mL of cold, anhydrous MeOH) to give **S2** as a dark red, iridescent solid (201 mg, 0.501 mmol, 45%) that was used without further purification. R_f in 1:5 acetone/toluene: 0.3. ^{1}H NMR (500 MHz, CD_3OD) δ 7.63 – 7.61 (m, 2H), 7.45 – 7.42 (m, 4H), 7.38 – 7.33 (m, 5H), 7.23 – 7.19 (m, 2H), 7.07 – 7.04 (m, 4H), 2.75 (t, *J* = 5.9 Hz, 4H), 2.14 – 2.08 (m, 2H). ^{13}C NMR (126 MHz, CD_3OD) δ 171.9, 152.6, 140.8, 136.8, 131.8, 131.0, 129.6, 127.4, 121.0, 120.1, 119.2, 24.1, 21.4. HRMS (ESI$^+$) calcd 365.2012, found 365.2013 for $C_{26}H_{25}N_2^+$ ([M]$^+$). *Note*: Heptamethine linker modifications are synthetically challenging to isolate in the aniline form due to hydrolysis and/or acetylation. However, both side products are synthetically competent for condensation with heterocycle, and are present in ca. < 5% in the final material.

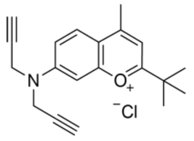

S7

2-(*tert*-butyl)-7-(di(prop-2-yn-1-yl)amino)-4-methylchromenylium chloride (**S7**): This synthesis has been previously reported.[2] Altering extraction conditions resulted in higher isolated yield and

purity. To a flask containing CuI (292 mg, 1.53 mmol, 3.00 equiv.), anhydrous THF (25 mL) was added and cooled in dry ice/acetone bath to -78 ˚C. MeLi (1.6 M in Et_2O, 1.9 mL, 3.0 mmol, 6.0 equiv.) was added dropwise and the mixture was stirred for 10 min. A solution of **S6** (150 mg, 0.511 mmol, 1.00 equiv.) in anhydrous THF (5 mL) was transferred to the reaction system dropwise and stirred for another 10 min. MeLi (1.6 M in Et_2O, 2.9 mL, 4.6 mmol, 9.0 equiv.) was then added. The reaction mixture was allowed to warm up to room temperature and stirred for another 15 min (or until the color changed from yellow to brown), followed by quenching by addition of 1:10 HCl (15 mL). The THF was removed by rotary evaporation, and the remaining solution was diluted with aqueous $Na_2S_2O_3$ (0.3 g/mL, 50 mL). This solution was extracted with DCM (4×50 mL), and the combined organic phase was dried (Na_2SO_4), concentrated, and triturated with Et_2O to give **S7** as a brown solid (161 mg, 0.491 mmol, 96%). ^{1}H NMR (500 MHz, $CDCl_3$) δ 8.33 (d, *J* = 10.7 Hz, 1H), 7.63 (dd, *J* = 9.6, 2.6 Hz, 1H), 7.48 (s, 1H), 7.20 (d, *J* = 2.6 Hz, 1H), 4.57 (s, 4H), 3.05 (s, 3H), 2.52 (t, *J* = 2.4 Hz, 2H), 1.57 (s, 9H). ^{13}C NMR (126 MHz, $CDCl_3$) δ 182.5, 167.7, 159.2, 156.2, 129.6, 119.5, 118.4, 113.7, 98.5, 76.9, 75.2, 42.1, 38.6, 28.6, 21.7. HRMS (ESI$^+$) calcd 292.1696, found 292.1689 for $C_{20}H_{22}NO^+$ ([M]$^+$). *Note*: organolithiates (such as MeLi) present an unusually high safety concern. To mitigate this, MeLi-containing vessels were handled carefully under an inert atmosphere until quenched with acid, and reaction scale was minimized.

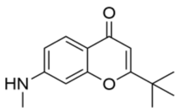

S8

2-(*tert*-butyl)-7-(methylamino)-4*H*-chromen-4-one (**S8**): 3-(methylamino)phenol) (470 mg, 3.82 mmol, 1.00 equiv.) and ethyl-4,4-dimethyl-3-oxopentanoate (1.97 g, 11.4 mmol, 3.00 equiv.) were combined in a 35 mL microwave reaction vessel. The reaction was carried out at 200 ˚C under 300 W microwave irradiation for 45 min, keeping pressure under 200 psi. The resulting dark brown oil was evaporated onto silica and purified by column chromatography (1:2 to 1:1 EtOAc/hexanes) to give **S8** as a red-brown solid (206 mg, 0.891 mmol, 23%). R_f in 1:1 EtOAc/hexanes: 0.2. ^{1}H NMR (500 MHz, $CDCl_3$) δ 7.89 (d, *J* = 8.7 Hz, 1H), 6.58 (dd, *J* = 8.7, 2.3 Hz, 1H), 6.39 (d, *J* = 2.1 Hz, 1H), 6.11 (s, 1H), 4.68 (s, 1H), 2.90 (s, 3H), 1.30 (s, 9H). ^{13}C NMR (126 MHz, $CDCl_3$) δ 178.4, 174.7, 159.1, 153.9, 126.3, 113.8, 112.5, 106.0, 95.7, 36.2, 30.1, 27.9. HRMS (ESI$^+$) calcd 232.1332, found 232.1335 for $C_{14}H_{18}NO_2^+$ ([M+H]$^+$).

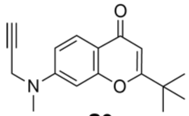

S9

2-(*tert*-butyl)-7-(methyl(prop-2-yn-1-yl)amino)-4*H*-chromen-4-one (**S9**): Compound **S8** (233 mg, 1.01 mmol, 1.00 equiv.), propargyl bromide (0.6 mL of 80% solution in toluene, 5.0 mmol, 5.0 equiv.), TBAB (973 mg, 3.02 mmol, 3.00 equiv.) and NaH (322 mg of 60% dispersion in mineral oil, 8.05 mmol, 8.00 equiv.) were mixed with anhydrous THF (25 mL). The reaction was stirred under N_2 at 25 ˚C for 6 h, and then quenched by careful addition of MeOH and H_2O, until bubbling ceased. The solution was concentrated, diluted in saturated $NaHCO_3$ (50 mL), and extracted with DCM (4×50 mL). The combined organic phase was dried (Na_2SO_4) and concentrated. The mixture

was evaporated onto silica and purified by column chromatography (1:2 EtOAc/hexanes) to give **S9** as a yellow solid (219 mg, 0.813 mmol, 81%). R_f in 1:1 EtOAc/hexanes: 0.5. ^{1}H NMR (500 MHz, $CDCl_3$) δ 8.04 (dd, *J* = 9.0, 2.0 Hz, 1H), 6.86 (dt, *J* = 9.0, 2.2 Hz, 1H), 6.67 – 6.63 (m, 1H), 6.17 (d, *J* = 2.0 Hz, 1H), 4.18 – 4.15 (m, 2H), 3.13 (d, *J* = 1.8 Hz, 3H), 2.26 (q, *J* = 2.4 Hz, 1H), 1.34 (s, 9H). ^{13}C NMR (126 MHz, $CDCl_3$) δ 178.2, 175.4, 158.5, 152.9, 126.6, 114.0, 111.7, 105.9, 98.8, 78.1, 72.6, 41.9, 38.5, 36.4, 27.9. HRMS (ESI$^+$) calcd 270.1489, found 270.1499 for $C_{17}H_{20}NO_2^+$ ([M+H]$^+$). Note: metal hydrides (such as NaH) are water-reactive and present an unusually high safety concern. To mitigate this, NaH-containing vessels were handled carefully under a dry, inert atmosphere until quenched with water.

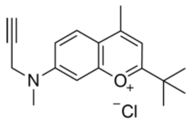

S10

2-(*tert*-butyl)-4-methyl-7-(methyl(prop-2-yn-1-yl)amino)chromenylium chloride (**S10**): To a flask containing CuI (212 mg, 1.11 mmol, 3.00 equiv.), anhydrous THF (25 mL) was added and cooled in dry ice/acetone bath to -78 ˚C. MeLi (1.6 M in Et_2O, 1.4 mL, 2.2 mmol, 6.0 equiv.) was added dropwise and the mixture was stirred for 10 min. A solution of **S9** (100 mg, 0.371 mmol, 1.00 equiv.) in anhydrous THF (5 mL) was transferred to the reaction system dropwise and stirred for another 10 min. MeLi (1.6 M in Et_2O, 2.1 mL, 3.3 mmol, 9.0 equiv.) was then added. The reaction mixture was allowed to warm up to room temperature and stirred for another 15 min (or until the color changed from yellow to brown), followed by quenching by addition of 1:10 HCl (12 mL). The mixture was then concentrated until precipitate begins to form, and then diluted with aqueous $Na_2S_2O_3$ (0.3 g/mL, 40 mL). This solution was extracted with DCM (4×40 mL), and the combined organic phase was dried (Na_2SO_4), concentrated, and triturated with Et_2O to give **S10** as a brown solid (110 mg, 0.362 mmol, 98%). ^{1}H NMR (600 MHz, $CDCl_3$) δ 8.24 (d, *J* = 9.6 Hz, 1H), 7.55 (d, *J* = 12.2 Hz, 1H), 7.32 (s, 1H), 7.03 (s, 1H), 4.47 (s, 2H), 3.43 (s, 3H), 2.97 (s, 3H), 2.45 (s, 1H), 1.52 (s, 9H). ^{13}C NMR (126 MHz, $CDCl_3$) δ 181.3, 166.1, 159.4, 157.3, 129.4, 119.2, 118.1, 112.6, 97.3, 76.7, 74.6, 43.4, 40.0, 38.4, 28.5, 21.4. HRMS (ESI$^+$) calcd 268.1696, found 268.1697 for $C_{18}H_{22}NO^+$ ([M]$^+$). *Note*: organolithiates (such as MeLi) present an unusually high safety concern. To mitigate this, MeLi-containing vessels were handled carefully under an inert atmosphere until quenched with acid, and reaction scale was minimized.

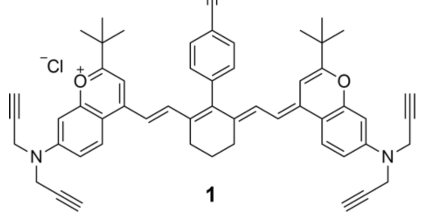

1

2-(*tert*-butyl)-4-((*E*)-2-((*E*)-6-(2-((*E*)-2-(*tert*-butyl)-7-(di(prop-2-yn-1-yl)amino)-4*H*-chromen-4-ylidene)ethylidene)-4'-ethynyl-3,4,5,6-tetrahydro-[1,1'-biphenyl]-2-yl)vinyl)-7-(di(prop-2-yn-1-yl)amino)chromenylium chloride (**1**): To a Schlenk tube containing **S1** (30 mg, 0.071 mmol, 0.45 equiv.), **S7** (50 mg, 0.15 mmol, 1.0 equiv.), and NaOAc (38 mg, 0.46 mmol, 3.0 equiv.), Ac_2O (3 mL) was added, followed by three freeze-pump-thaw cycles to fill in N_2. The reaction mixture was

stirred at 37 ˚C for 45 min, followed by evaporating onto silica for column chromatography (1% EtOH in DCM). The fluorophore was then isolated with reverse phase preparatory HPLC, running a gradient from 80 to 100% MeCN in H_2O with 0.1% TFA to give **1** (13 mg, 0.016 mmol, 23%) as a dark red/brown solid. R_f in 1:50 EtOH/DCM: 0.1. ^{1}H NMR (500 MHz, CD_2Cl_2) δ 7.98 (d, *J* = 9.5 Hz, 2H), 7.70 (d, *J* = 8.4 Hz, 2H), 7.34 (d, *J* = 8.5 Hz, 2H), 7.26 (d, *J* = 13.6 Hz, 2H), 7.09 (dd, *J* = 9.3, 2.6 Hz, 2H), 6.91 (d, *J* = 13.7 Hz, 2H), 6.83 (d, *J* = 2.6 Hz, 2H), 6.25 (s, 2H), 4.33 (d, *J* = 2.4 Hz, 8H), 3.28 (s, 1H), 2.83 (t, *J* = 5.9 Hz, 4H), 2.47 – 2.41 (m, 4H), 2.10 (t, *J* = 5.8 Hz, 2H), 1.29 (s, 18H). ^{13}C NMR (126 MHz, CD_2Cl_2) δ 170.6, 160.8, 156.2, 151.7, 146.4, 144.7, 138.8, 135.1, 131.4, 131.0, 125.4, 122.2, 113.9, 113.3, 110.3, 100.2, 99.8, 82.6, 77.9, 77.5, 73.3, 40.4, 36.5, 27.4, 25.3, 21.3. HRMS (ESI$^+$) calcd 785.4102, found 785.4105 for $C_{56}H_{53}N_2O_2^+$ ([M]$^+$). Absorbance (DCM, MeOH): 937 nm, 927 nm. Emission (DCM, MeOH): 952 nm, 943 nm.

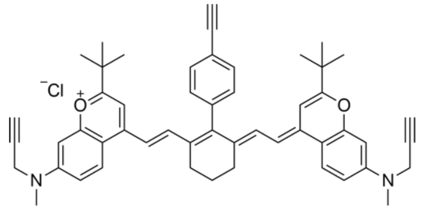

3

2-(*tert*-butyl)-4-((*E*)-2-((*E*)-6-(2-((*E*)-2-(*tert*-butyl)-7-(methyl(prop-2-yn-1-yl)amino)-4*H*-chromen-4-ylidene)ethylidene)-4'-ethynyl-3,4,5,6-tetrahydro-[1,1'-biphenyl]-2-yl)vinyl)-7-(methyl(prop-2-yn-1-yl)amino)chromenylium chloride (**3**): To a Schlenk tube containing **S1** (10 mg, 0.024 mmol, 0.45 equiv.), **S10** (35 mg, 0.12 mmol, 2.2 equiv.), and NaOAc (17 mg, 0.21 mmol, 4.0 equiv.), Ac_2O (4 mL) was added followed by three freeze-pump-thaw cycles to fill in N_2. The reaction was stirred at 25 ˚C for 14 h, followed by evaporating onto silica for column chromatography (1.2% EtOH in DCM). The fluorophore was then isolated with reverse phase preparatory HPLC, running a gradient from 80% to 100% MeCN in H_2O with 0.1% TFA to give **3** as a dark red/brown solid (8 mg, 0.01 mmol, 44%). R_f in 1:50 EtOH/DCM: 0.1. ^{1}H NMR (600 MHz, CD_2Cl_2) δ 7.93 (d, *J* = 9.5 Hz, 2H), 7.69 (d, *J* = 8.4 Hz, 2H), 7.33 (d, *J* = 8.4 Hz, 2H), 7.22 (d, *J* = 13.6 Hz, 2H), 7.00 (dd, *J* = 9.4, 2.6 Hz, 2H), 6.88 (d, *J* = 13.7 Hz, 2H), 6.70 (d, *J* = 2.5 Hz, 2H), 6.22 (s, 2H), 4.26 (d, *J* = 2.5 Hz, 4H), 3.27 (s, 1H), 3.21 (s, 6H), 2.82 (t, *J* = 6.0 Hz, 4H), 2.39 (t, *J* = 2.4 Hz, 2H), 2.09 (t, *J* = 6.2 Hz, 2H), 1.28 (s, 18H). ^{13}C NMR (126 MHz, CD_2Cl_2) δ 170.7, 160.4, 156.7, 153.5, 146.5, 144.5, 139.4, 134.9, 131.8, 131.4, 125.7, 122.5, 113.7, 112.9, 110.4, 99.9, 99.4, 83.0, 78.2, 78.1, 73.1, 42.3, 38.9, 36.8, 30.0, 27.8, 25.8. HRMS (ESI$^+$) calcd 737.4102, found 737.4125 for $C_{52}H_{53}N_2O_2^+$ ([M$^+$]). Absorbance (DCM, MeOH): 944 nm, 933 nm. Emission (DCM, MeOH): 962 nm, 957 nm.

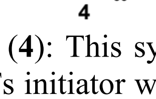

4

Methyl-poly(2-methyl-2-oxazoline)-azide (**4**): This synthesis has been previously reported.[12] In this work the reported rates for the MeOTs initiator were adapted to a MeOTf initiator. Reaction concentrations and purification conditions were also modified to result in a more monodisperse

polymer population. To a flame-dried microwave reaction vessel under N_2, anhydrous MeCN (9.7 mL, 2.0 M), and distilled 2-methyl-2-oxazoline (2.00 g, 23.5 mmol, 30.0 equiv.) were added. Distilled methyl triflate (89 µL, 0.78 mmol, 1.0 equiv.) was then added and the solution was heated at 140 ˚C under 300 W microwave irradiation for 6.7 min (98% monomer consumption). The reaction was then cooled to 25 ˚C before adding sodium azide (509 mg, 7.83 mmol, 10.0 equiv.). The slurry was stirred at 25 ˚C for 16 h, filtered over celite (to remove excess azide salts) and then concentrated. The solid mixture was redissolved in a minimal volume of $CHCl_3$ (2-4 mL) and precipitated into cold Et_2O (40-80 mL). The precipitate was collected, redissolved in water, and dialyzed against water with a 1 kDa MWCO membrane for 24 h. Lyophilization gave polymer **4** as a fluffy, white hygroscopic powder (1.87 g, 0.716 mmol, 94% mass recovery). ^{1}H NMR (500 MHz, CD_3OD) δ 3.67 – 3.49 (m, 120H), 3.14 – 2.96 (m, 3H), 2.20 – 2.09 (m, 91H). Target molecular weight is 2608.62 for $C_{121}H_{213}N_{33}O_{30}$ (n = 30). NMR calcd 2609 (M_n). MALDI-TOF calcd 2656 (M_n), 2730 (M_w), and *Đ* of 1.03. GPC (dUV 210 nm) calcd 2498 (M_n), 2654 (M_w), and *Đ* of 1.06. The average of these values is closest to n = 30, which was used to determine stoichiometry for post-polymerization modifications (see "General Procedure for CuAAC", below). *Note*: NaN_3 presents an unusually high safety concern. To mitigate this, NaN_3-containing vessels were handled carefully under a dry, inert atmosphere (away from metals and acid) until removal of excess azide salts (disposed as a basic, aqueous hazardous waste solution).

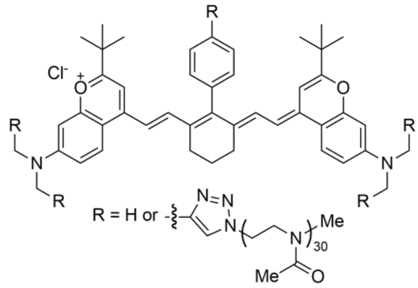

General Procedure for CuAAC: To a dram vial containing **1**, **2**, or **3** (1.0 mg, 1.0 equiv.), was added **4** (10 equiv.), $CuSO_4$ (0.16 equiv.), THPTA (0.80 equiv.), NaAsc (8.2 equiv.), and 2:1 MeOH/H_2O for a total volume of 2 mL. The solution was degassed by three freeze-pump-thaw cycles to fill in N_2, and then stirred at 37 ˚C for 5 h. After 5 h Na_2EDTA (4.1 equiv.) was added and the solution was stirred at 25 ˚C for 10-20 min, transferred to a dialysis membrane (2 kDa MWCO) and dialyzed against H_2O for 4 d. Size exclusion chromatography with H_2O followed by lyophilization was used to isolate **5** (**CStar30**), **6**, or **7**. These materials were analyzed via absorbance, emission, NMR, MALDI-TOF/TOF, and GPC (dUV 210 nm to detect other species containing precursor **4**, and dUV 550 nm to detect other species containing fluorophore), as well as an internal GPC calibration to better represent the star polymer architecture (see "CStar GPC Calibration" and Figure S6, S7).

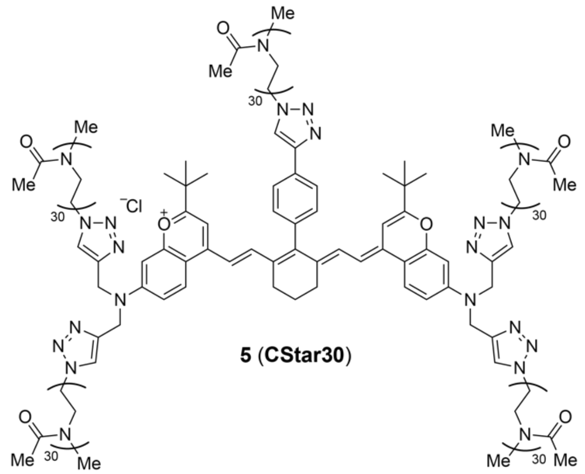

CStar30 (**5**): Following the "General Procedure for CuAAC," **1** (1.0 mg, 1.2 µmol, 1.0 equiv.) was reacted with **4** (32 mg, 12 µmol, 10 equiv.), $CuSO_4$ (20 µL of 10 mM solution in H_2O, 0.20 µmol, 0.16 equiv.), THPTA (20 µL of 50 mM solution in H_2O, 1.0 µmol, 0.80 equiv.), and NaAsc (20 µL of 500 mM solution made fresh in H_2O, 10 µmol, 8.2 equiv.) to afford **CStar30** (**5**) as a fluffy pink/brown powder (9.5 mg, 0.68 µmol, 57%). ^1H NMR (600 MHz, CD_3OD) δ 8.54 (m, 1H), 8.05 – 7.97 (m, 2 H), 7.40 (s, 1 H), 7.30 (s, 1H), 7.18 (s, 1H), 6.93 (m, 1 H), 6.19 (s, 1H), 3.67 – 3.43 (m, 73H), 2.23 – 2.00 (m, 59H), 1.29 (s, 11H). Target molecular weight is 13863.46 for $C_{661}H_{1118}N_{167}O_{152}$ (n = 30). MALDI-TOF/TOF calcd 13733 (M_n), 14013 (M_w), and $Đ$ of 1.02. GPC (dUV 210 nm) calcd 12306 (M_n), 12541 (M_w), and $Đ$ of 1.02. GPC (dUV 550 nm) calcd 12286 (M_n), 12492 (M_w), and $Đ$ of 1.02. The average of four replicate experiments via GPC is 12290 ± 70 (M_n), 12500 ± 100 (M_w). Absorbance (MeOH, H_2O, FBS): 939 nm, 940 nm, 940 nm. Emission (MeOH, H_2O, FBS): 958 nm, 953 nm, 954 nm. Cu (ICP-MS): 11.3 ± 0.2 ng/mg. *Note*: ^1H NMR is not reliable for large polymer species size determination.[6,13]

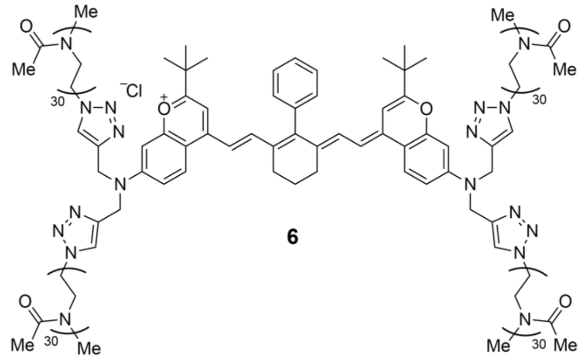

CStar **6**: Following the "General Procedure for CuAAC," **2** (1.0 mg, 1.3 µmol, 1.0 equiv.) was reacted with **4** (33 mg, 13 µmol, 10 equiv.), $CuSO_4$ (20 µL of 10 mM solution in H_2O, 0.20 µmol, 0.16 equiv.), THPTA (20 µL of 50 mM solution in H_2O, 1.0 µmol, 0.80 equiv.), and NaAsc (20 µL of 500 mM solution made fresh in H_2O, 10 µmol, 8.2 equiv.) to afford **6** as a fluffy pink/brown powder (7.0 mg, 0.62 µmol, 48%). ^1H NMR (600 MHz, CD_3OD) δ 8.56 (m, 1H), 8.09 – 8.01 (m, 2H), 7.43 (s, 1H), 7.31 (s, 1H), 7.13 (s, 1H), 6.99 (s, 1H), 6.78 (s, 1H), 6.21 (s, 1H), 3.70 – 3.46 (m, 86H), 3.14 – 2.96 (m, 3H), 2.21 – 2.07 (m, 64H), 1.31 (s, 2H). Target molecular weight is 11230.84 for $C_{538}H_{905}N_{134}O_{122}$ (n = 30). MALDI-TOF/TOF calcd 11282 (M_n), 11522 (M_w), and $Đ$

of 1.02. GPC (dUV 210 nm) calcd 11000 (M_n), 11146 (M_w), and *Đ* of 1.01. GPC (dUV 550 nm) calcd 11060 (M_n), 11137 (M_w), and *Đ* of 1.01. The average of four replicate experiments via GPC is 11130 ± 70 (M_n), 11260 ± 80 (M_w). Absorbance (MeOH, H_2O): 938 nm, 939 nm. Emission (MeOH, H_2O): 953 nm, 951 nm. *Note*: ^{1}H NMR is not reliable for large polymer species size determination.[6,13]

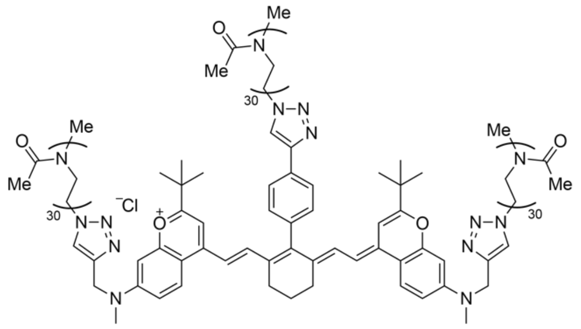

7

CStar **7**: Following the "General Procedure for CuAAC," **3** (1.0 mg, 1.3 µmol, 1.0 equiv.) was reacted with **4** (34 mg, 13 µmol, 10 equiv.), $CuSO_4$ (20 µL of 10 mM solution in H_2O, 0.20 µmol, 0.16 equiv.), THPTA (20 µL of 50 mM solution in H_2O, 1.0 µmol, 0.80 equiv.), and NaAsc (20 µL of 500 mM solution made fresh in H_2O, 10 µmol, 8.2 equiv.) to afford **7** as a fluffy purple/brown powder (3.6 mg, 0.42 µmol, 32%). ^{1}H NMR (600 MHz, CD_3OD) δ 8.11 – 7.99 (m, 2H), 7.60 – 7.57 (m, 1H), 7.34 – 7.19 (m, 2H), 6.98 (s, 2H), 6.19 (s, 1H), 3.69 – 3.46 (m, 93H), 3.14 – 2.96 (m, 3H), 2.21 – 2.08 (m, 70H), 1.31 (s, 1H). Target molecular weight is 8598.23 for $C_{415}H_{692}N_{101}O_{92}$ (n = 30). MALDI-TOF/TOF calcd 8687 (M_n), 8762 (M_w), and *Đ* of 1.01. GPC (dUV 210 nm) calcd 9180 (M_n), 9357 (M_w), and *Đ* of 1.02. GPC (dUV 550 nm) calcd 9195 (M_n), 9323 (M_w), and *Đ* of 1.01. The average of four replicate experiments via GPC is 9140 ± 60 (M_n), 9280 ± 60 (M_w). Absorbance (MeOH, H_2O): 941 nm, 945 nm. Emission (MeOH, H_2O): 965 nm, 961 nm. *Note*: ^{1}H NMR is not reliable for large polymer species size determination.[6,13]

^{1}H NMR Spectra

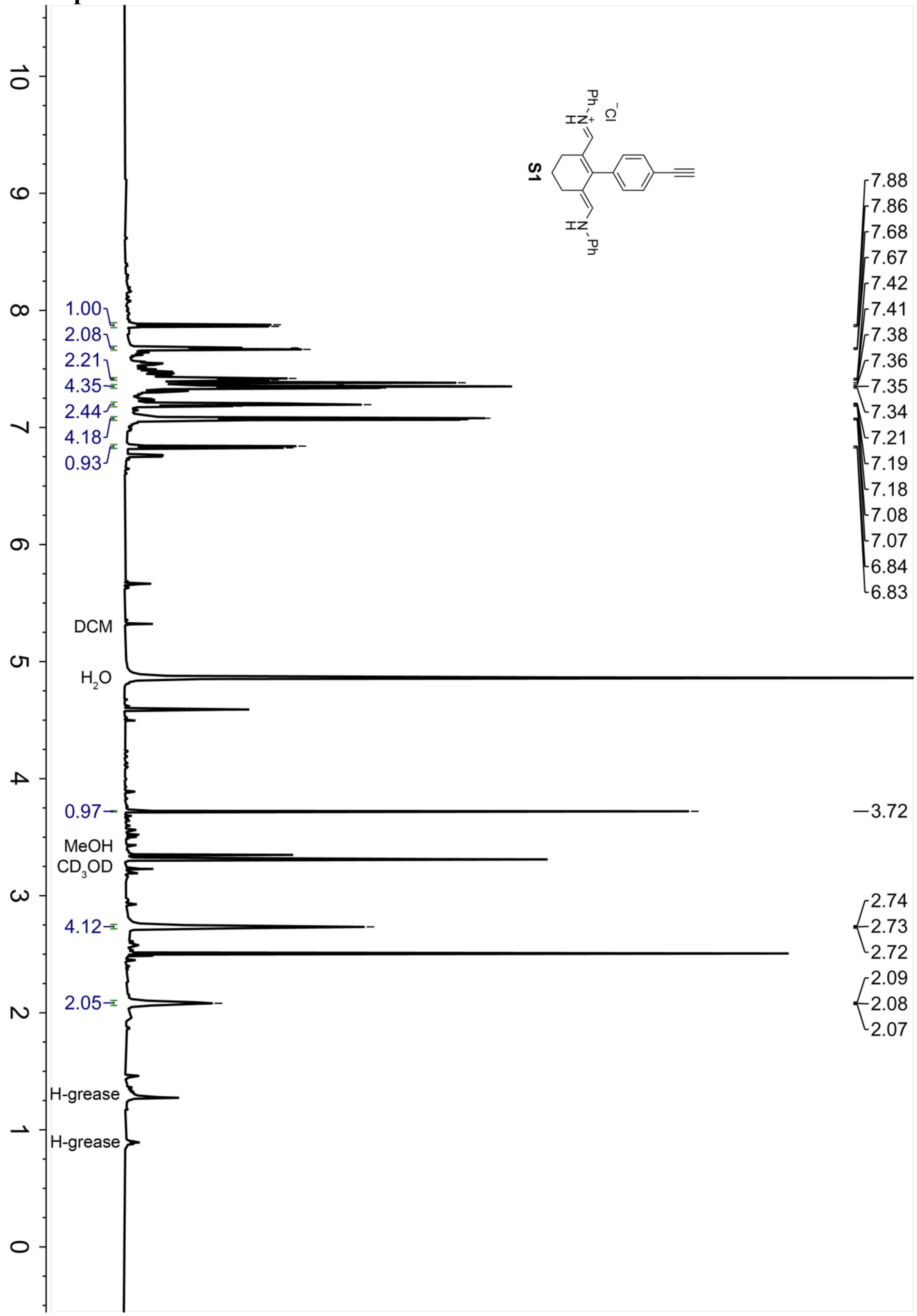

^{1}H NMR (600 MHz, CD_3OD) of **S1**.

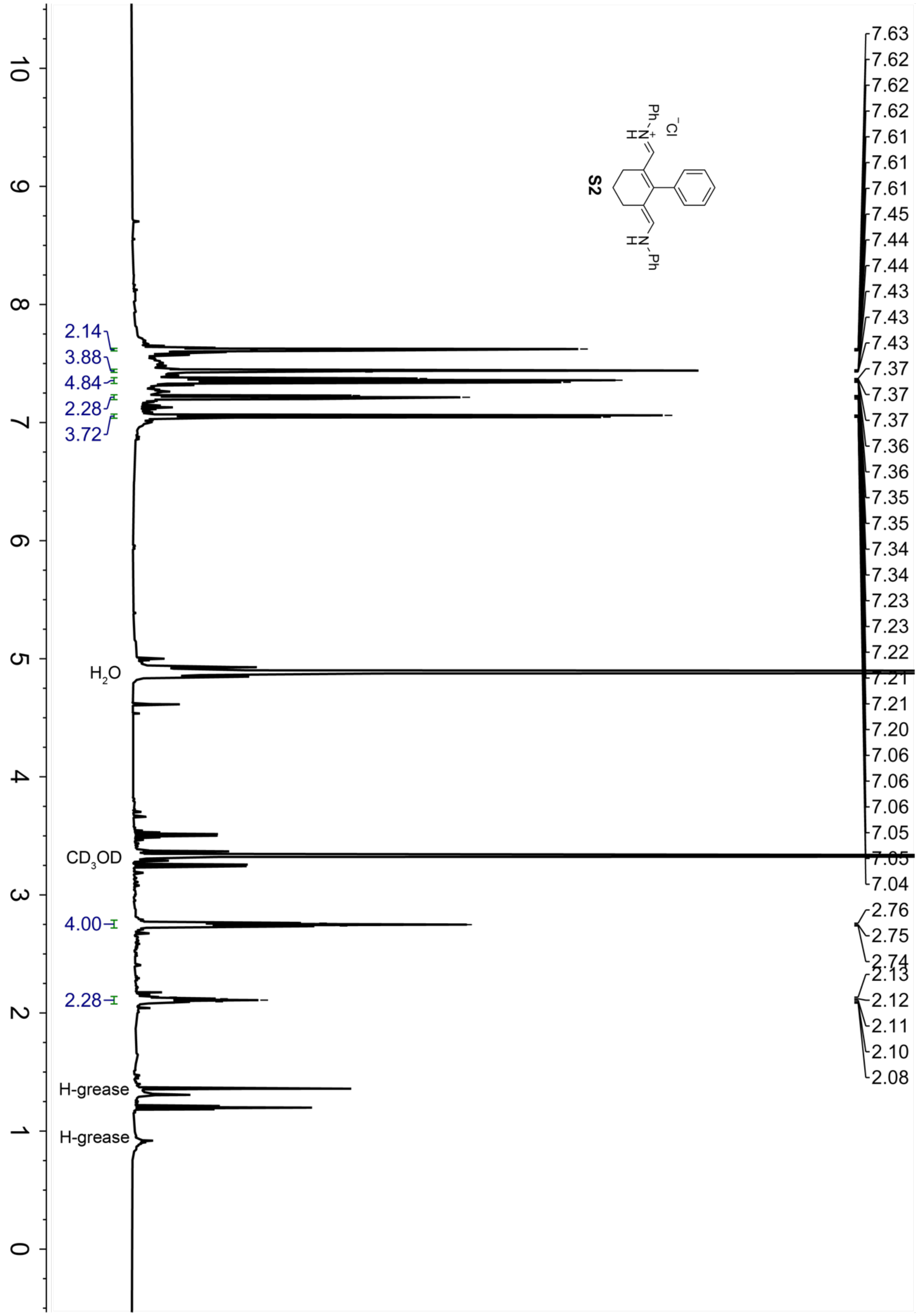

^{1}H NMR (500 MHz, CD_3OD) of **S2**.

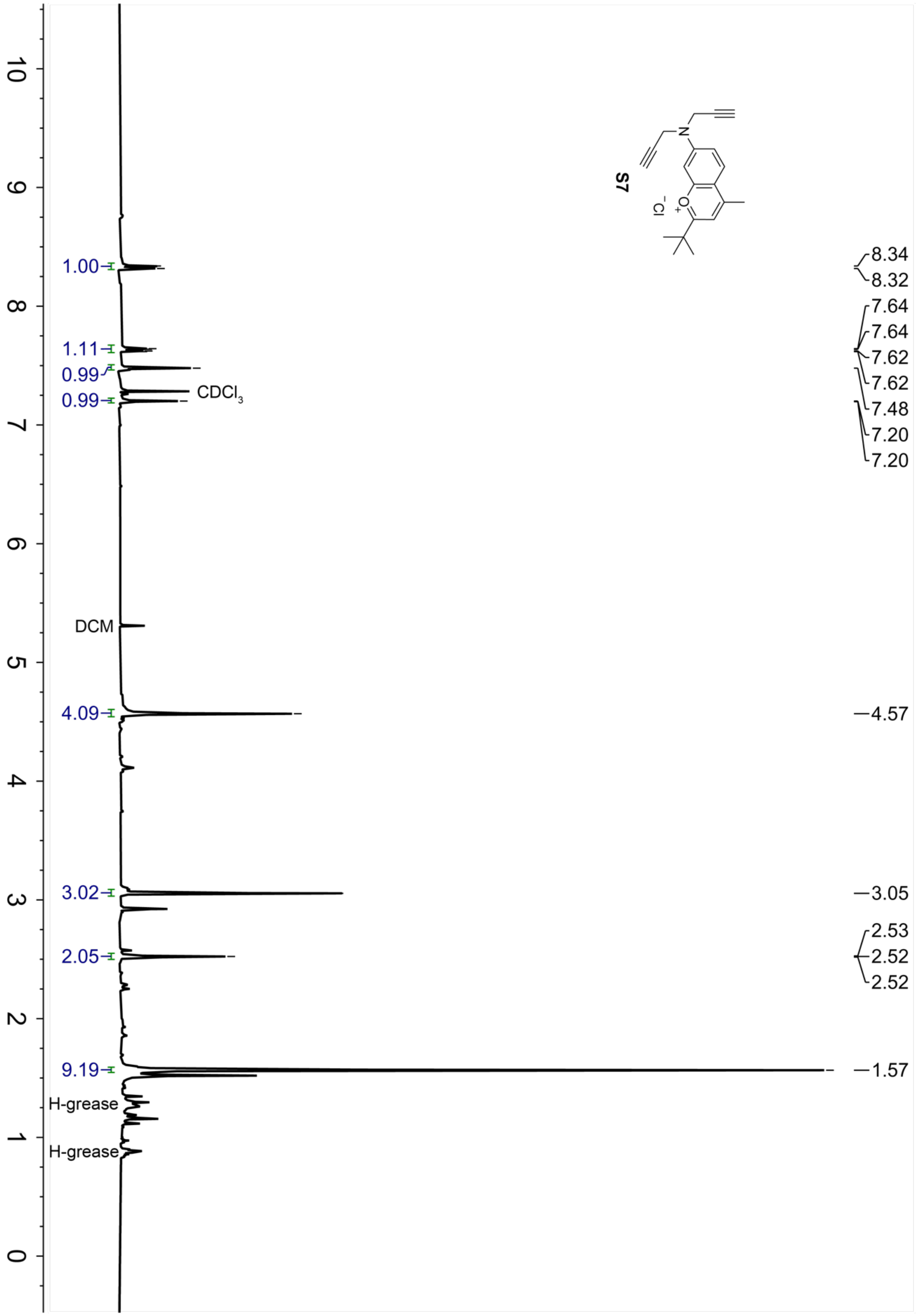

^{1}H NMR (500 MHz, $CDCl_3$) of **S7**.

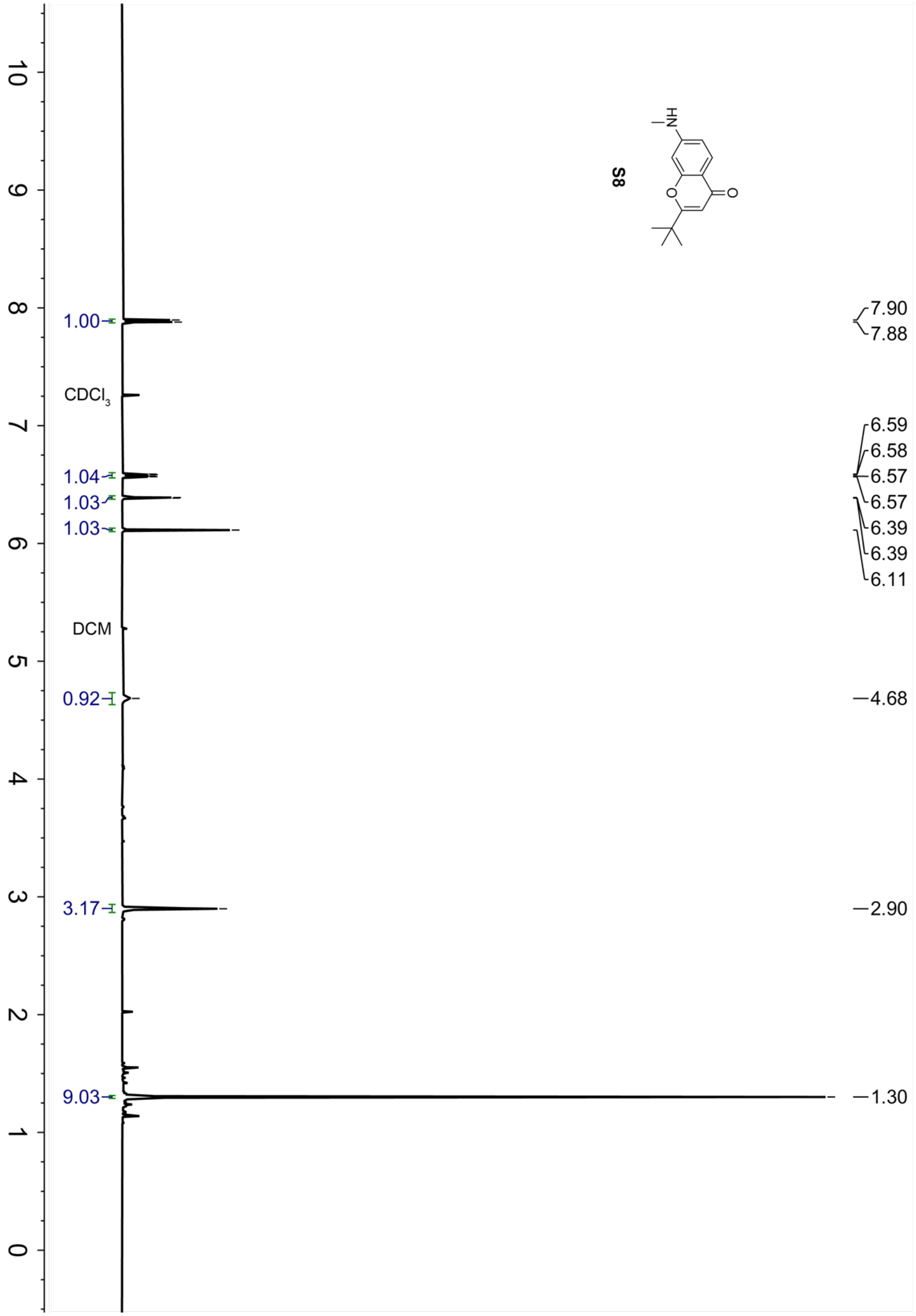

^{1}H NMR(500 MHz, $CDCl_3$) of **S8**.

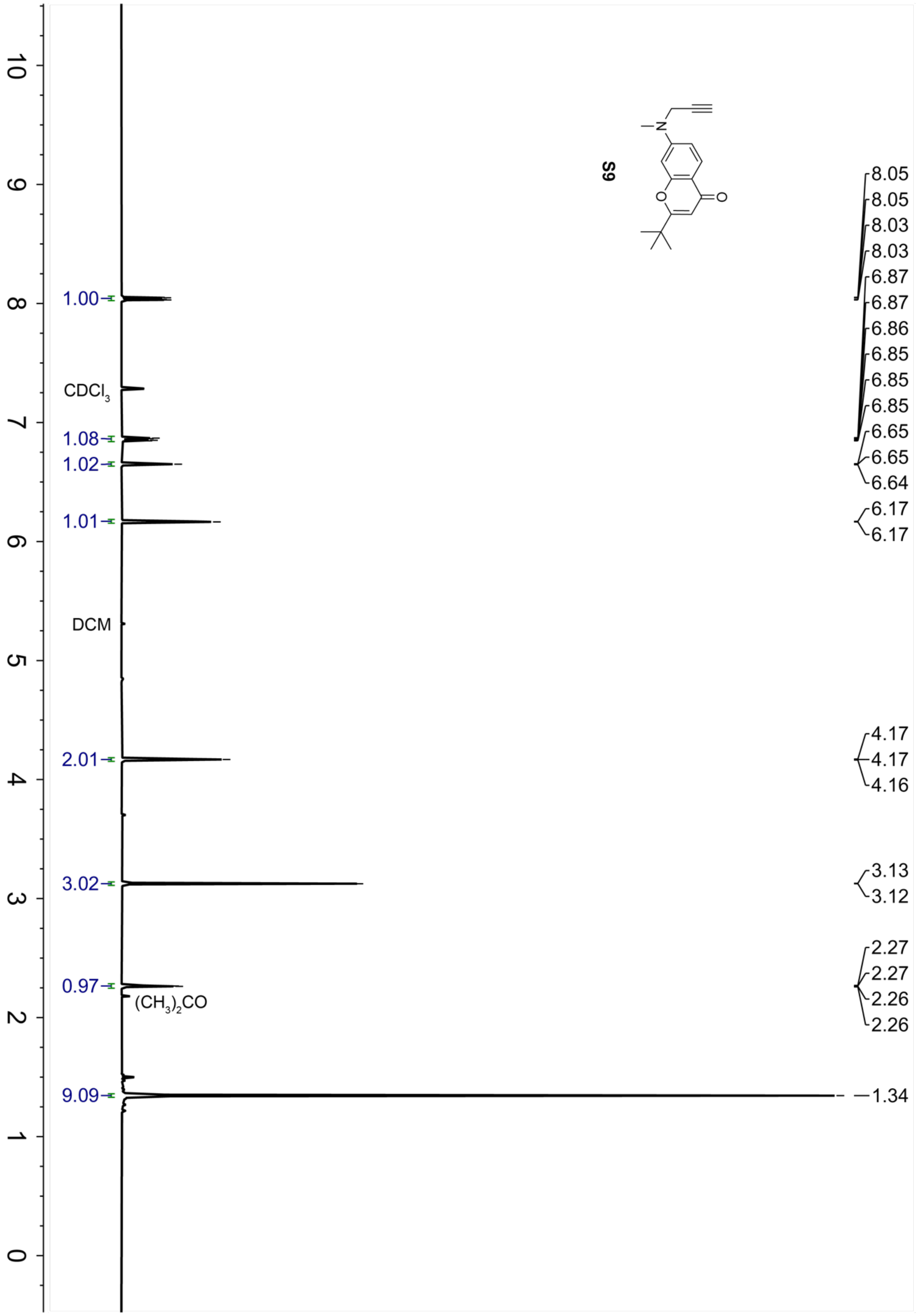

^{1}H NMR (500 MHz, $CDCl_3$) of **S9**.

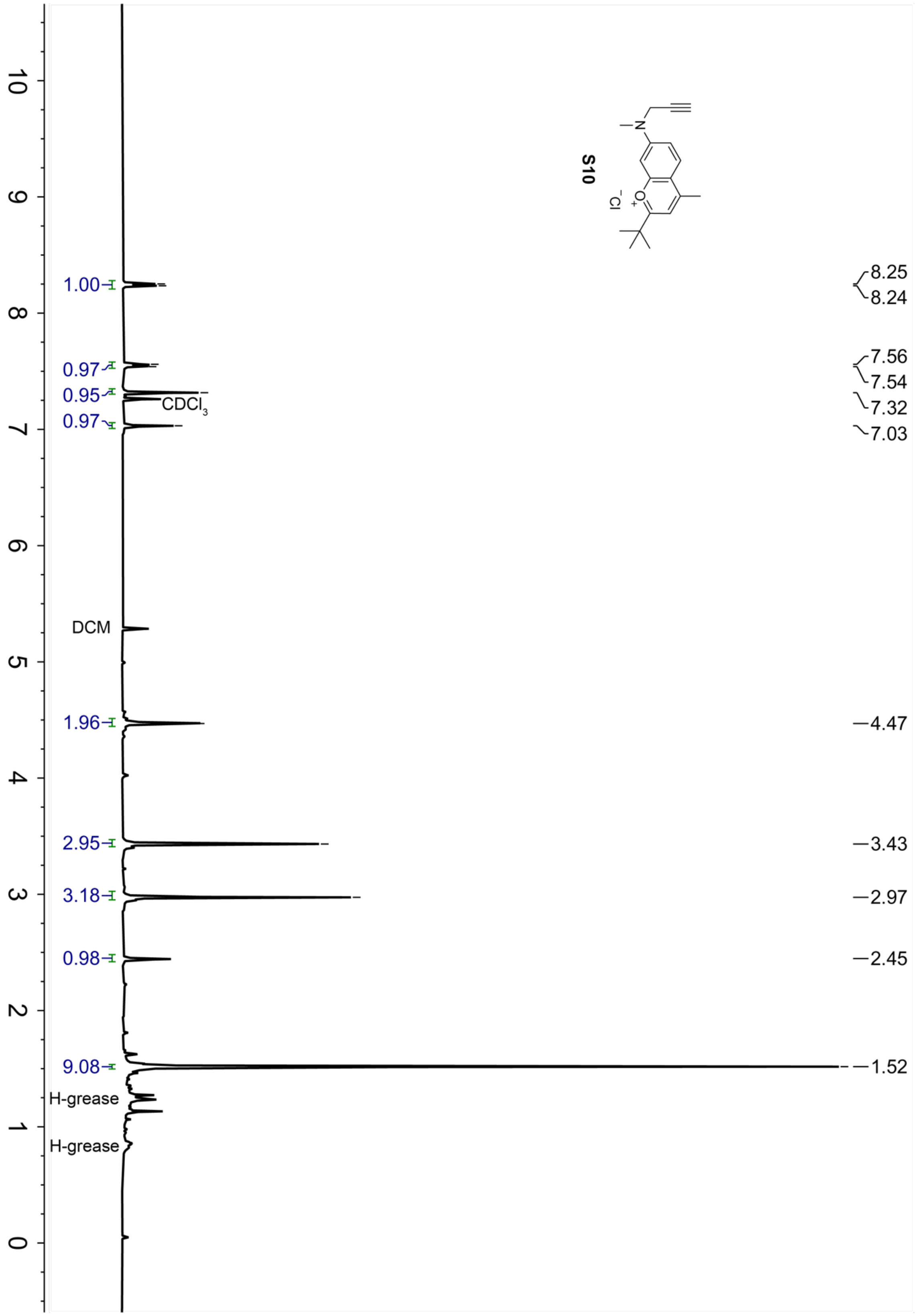

^{1}H NMR (600 MHz, $CDCl_3$) of **S10**.

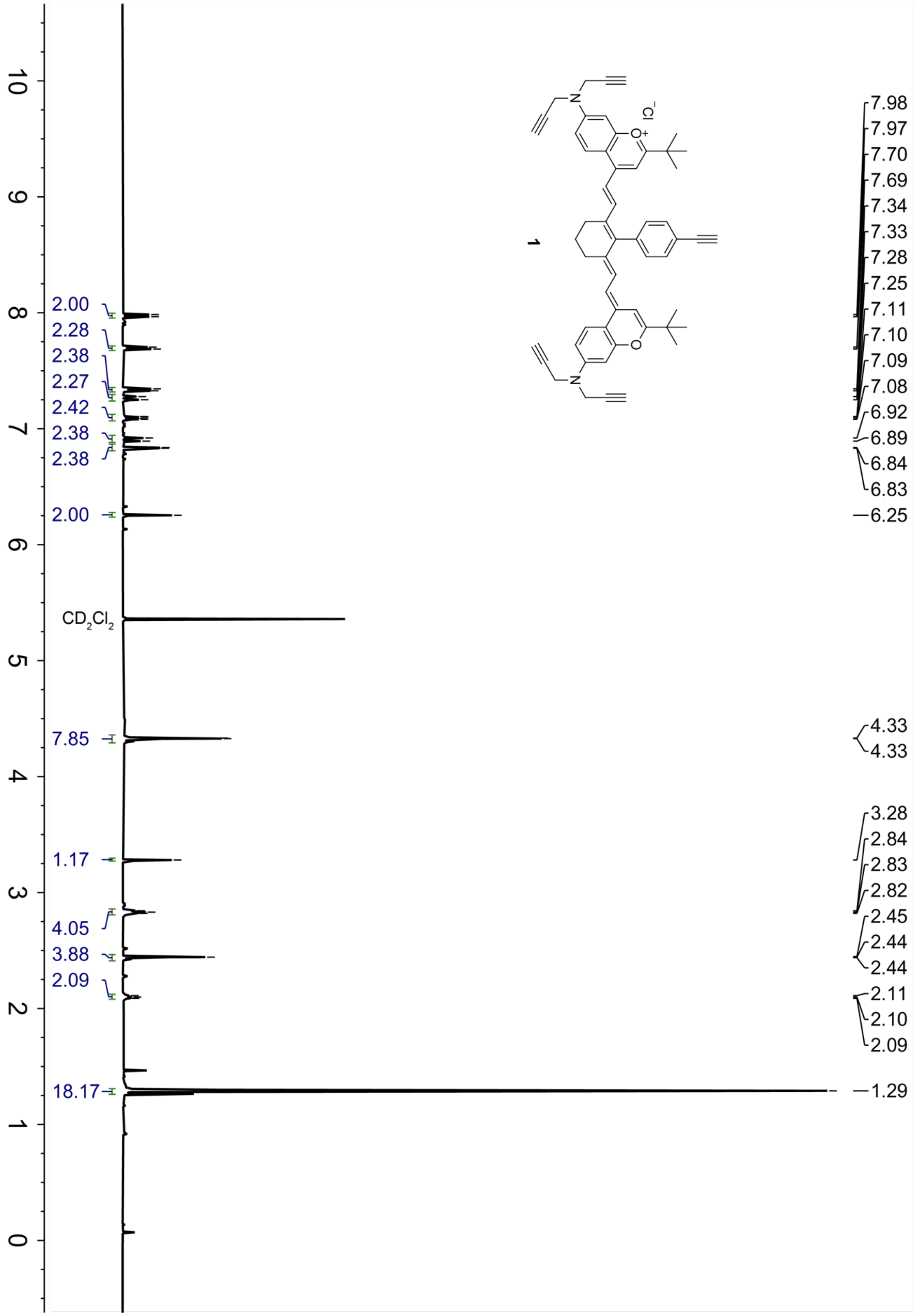

^{1}H NMR (500 MHz, CD_2Cl_2) of **1**.

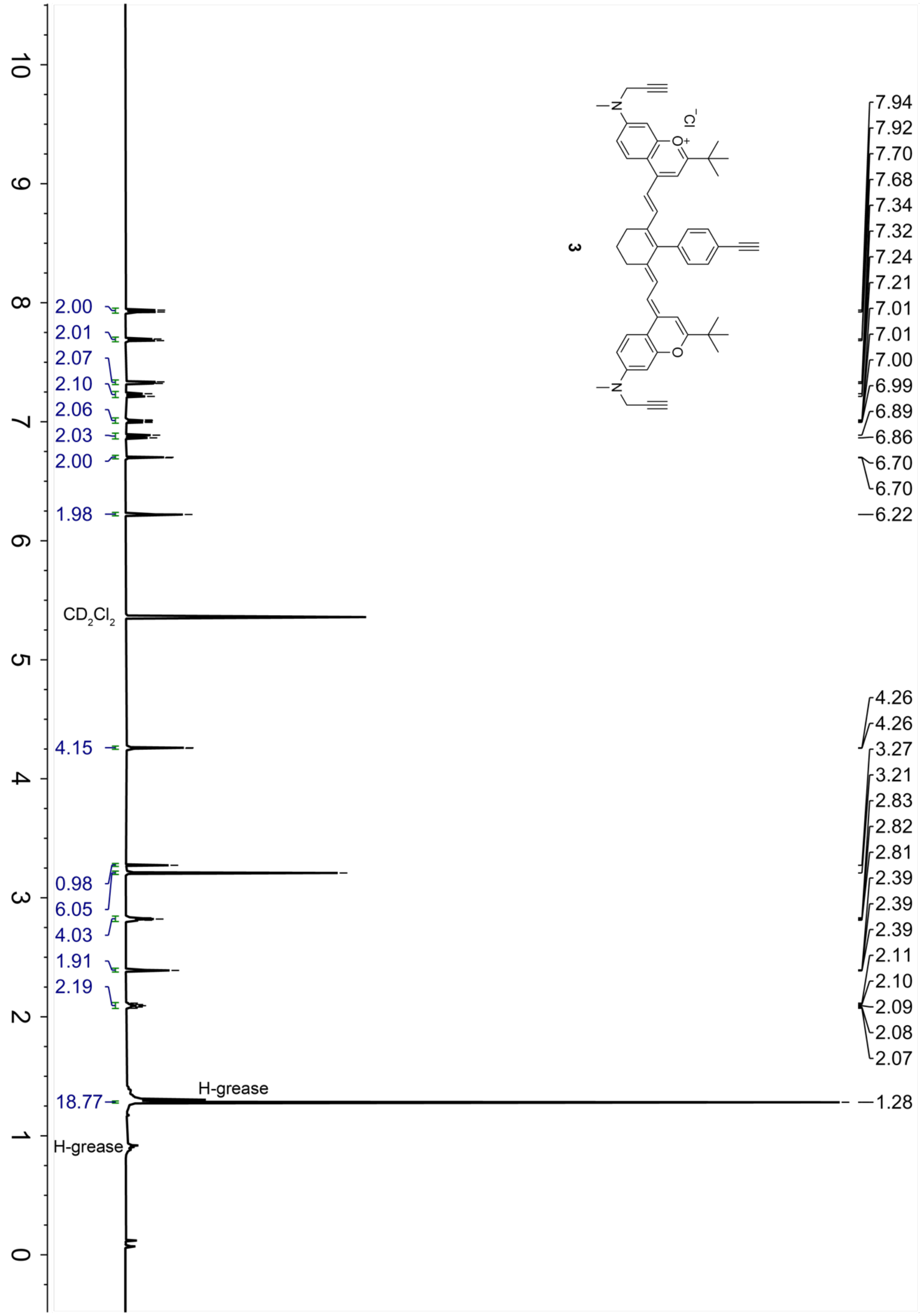

^{1}H NMR (600 MHz, CD_2Cl_2) of **3**.

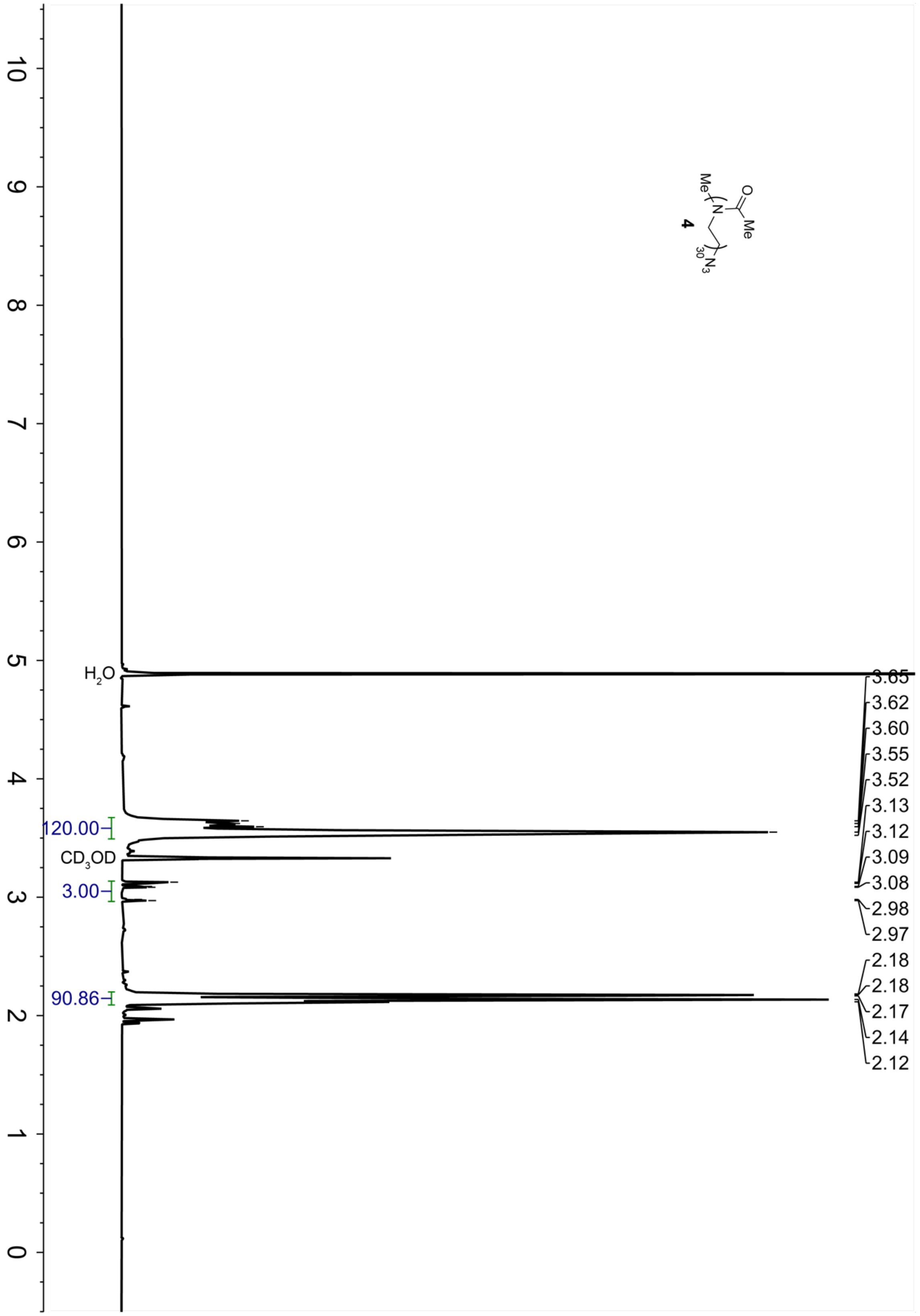

^{1}H NMR (500 MHz, CD_3OD) of **4**.

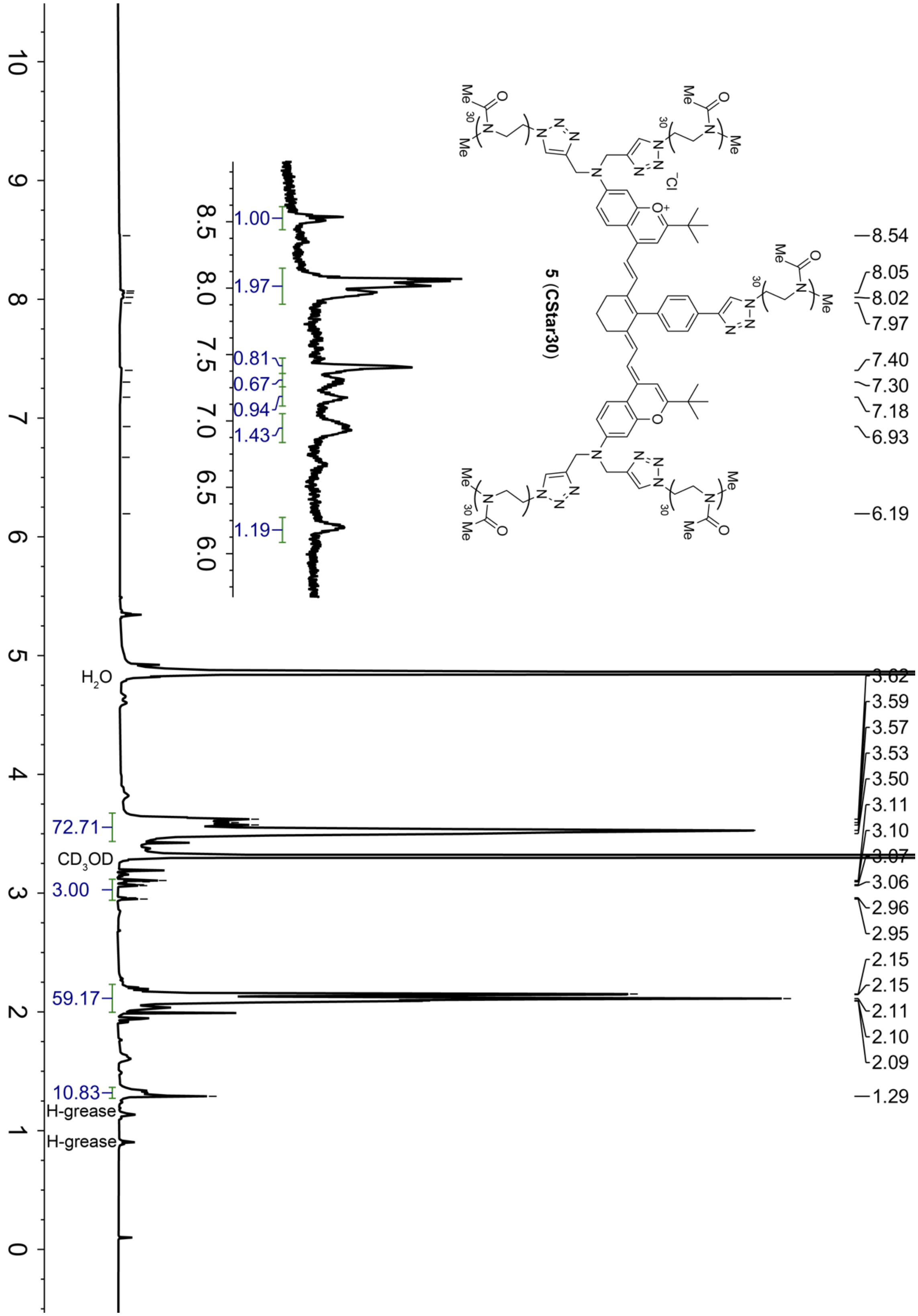

^{1}H NMR (600 MHz, CD_3OD) of **5** (**CStar30**).

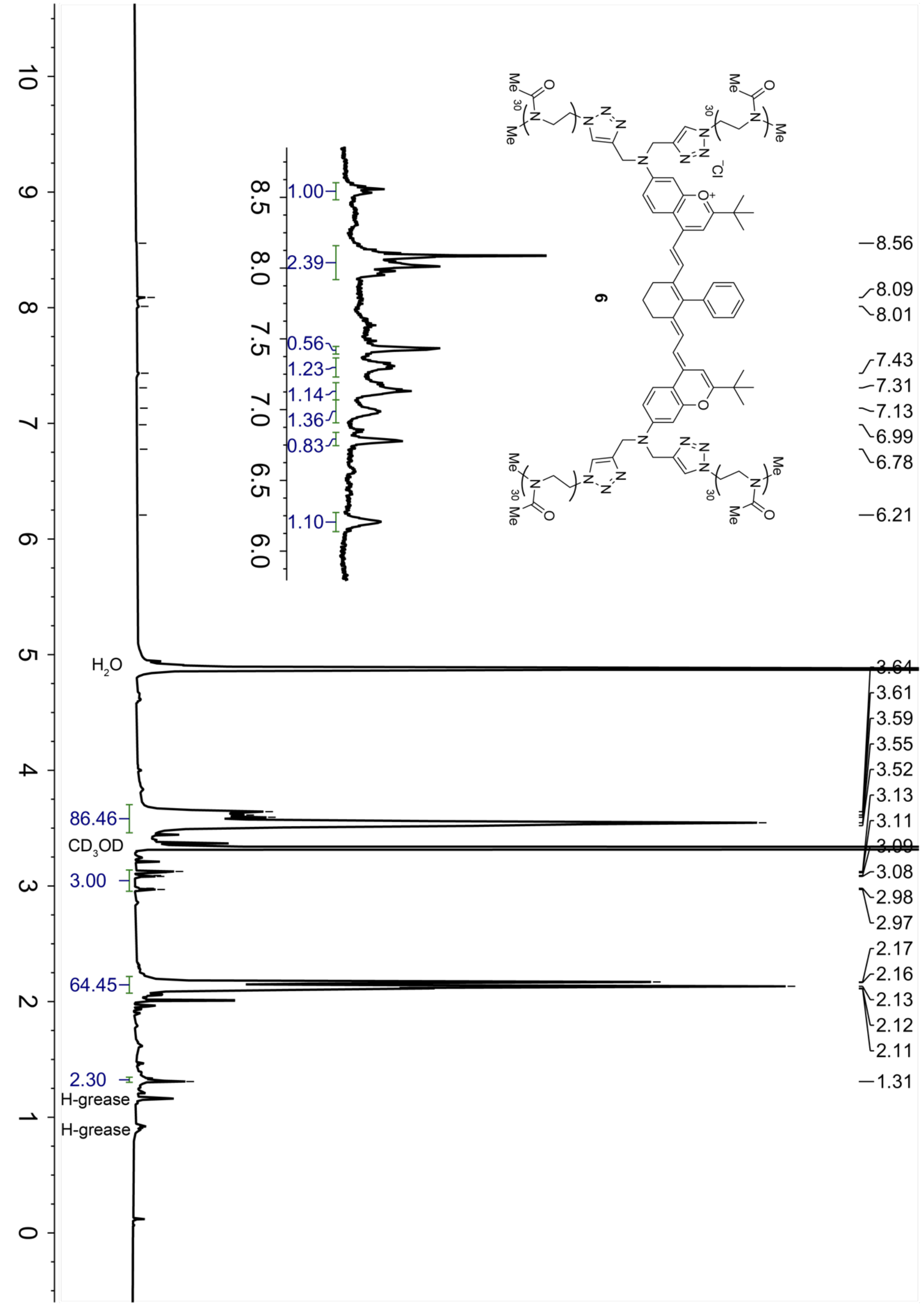

^{1}H NMR (600 MHz, CD_3OD) of **6**.

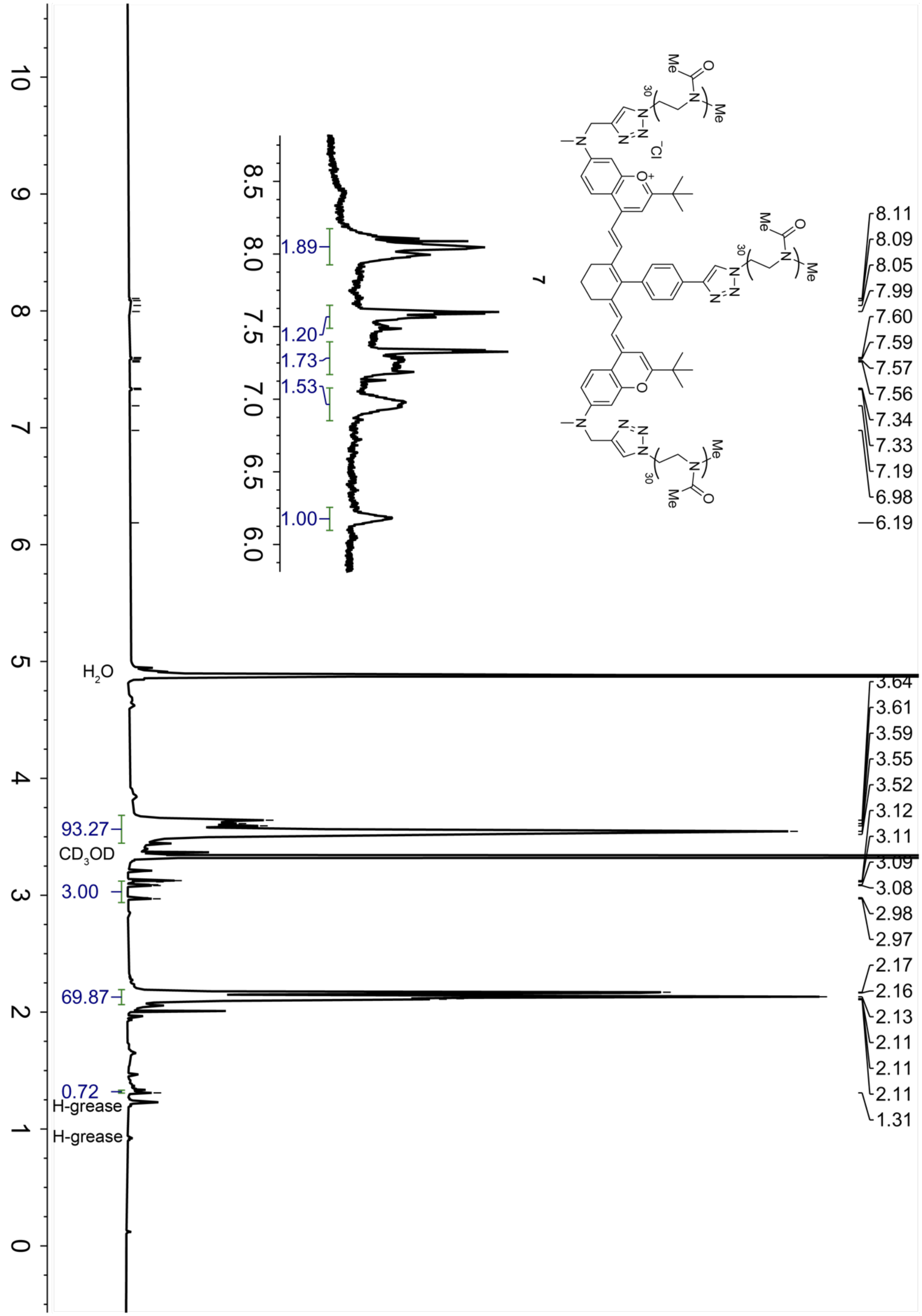

^{1}H NMR (600 MHz, CD_3OD) of **7**.

^{13}C NMR Spectra

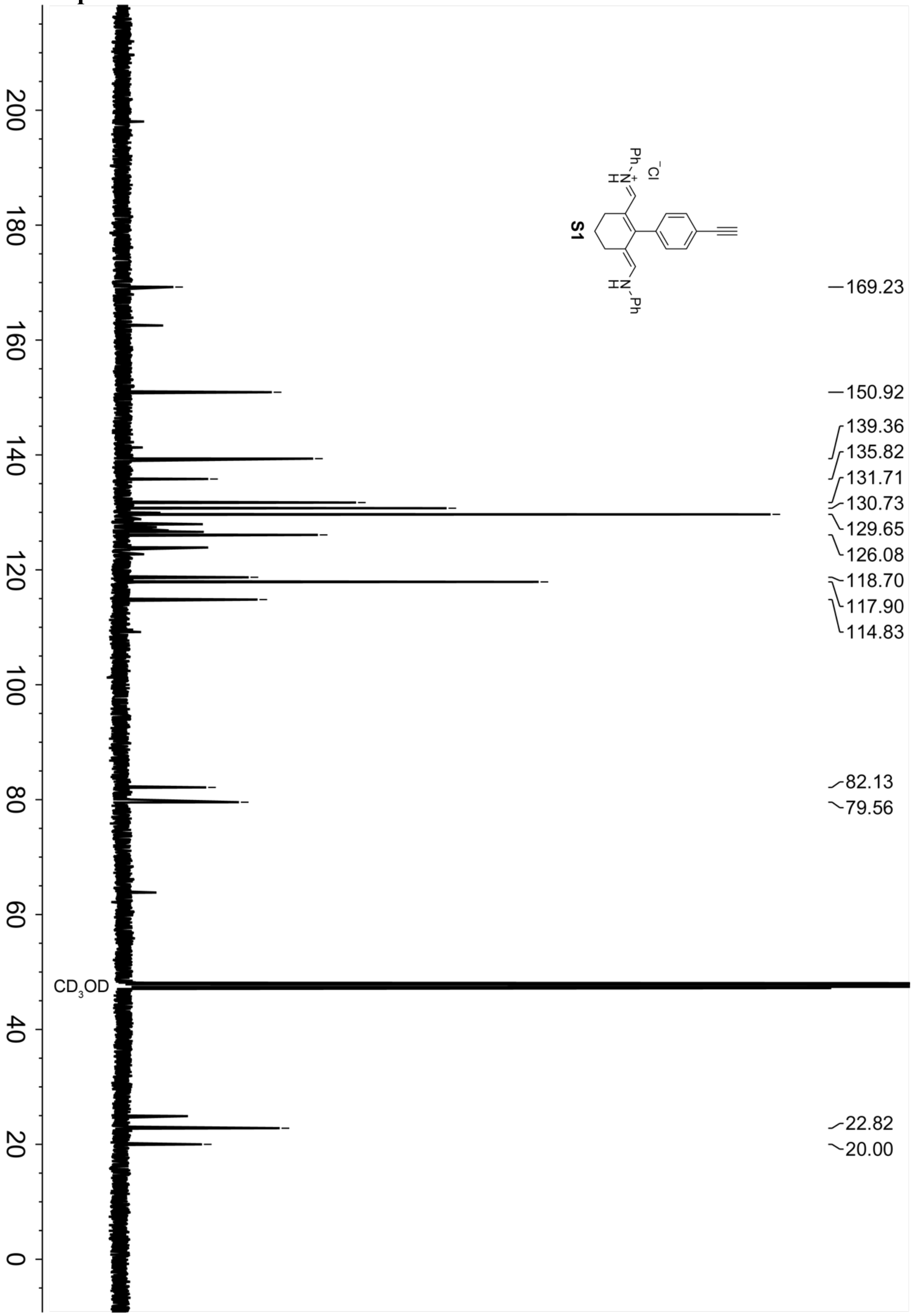

^{13}C NMR (126 MHz, $CDCl_3$) of **S1**.

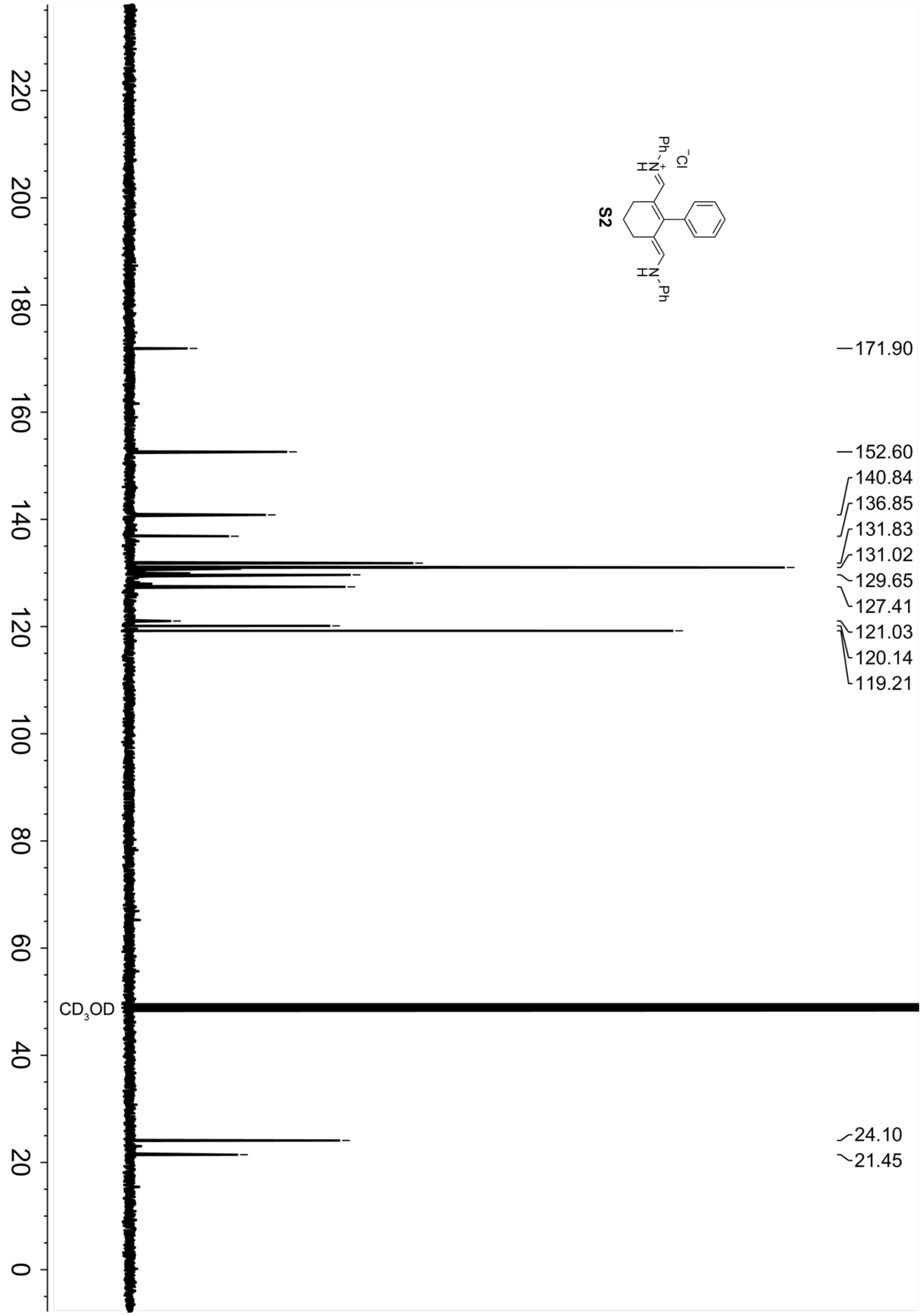

^{13}C NMR(126 MHz, $CDCl_3$) of **S2**.

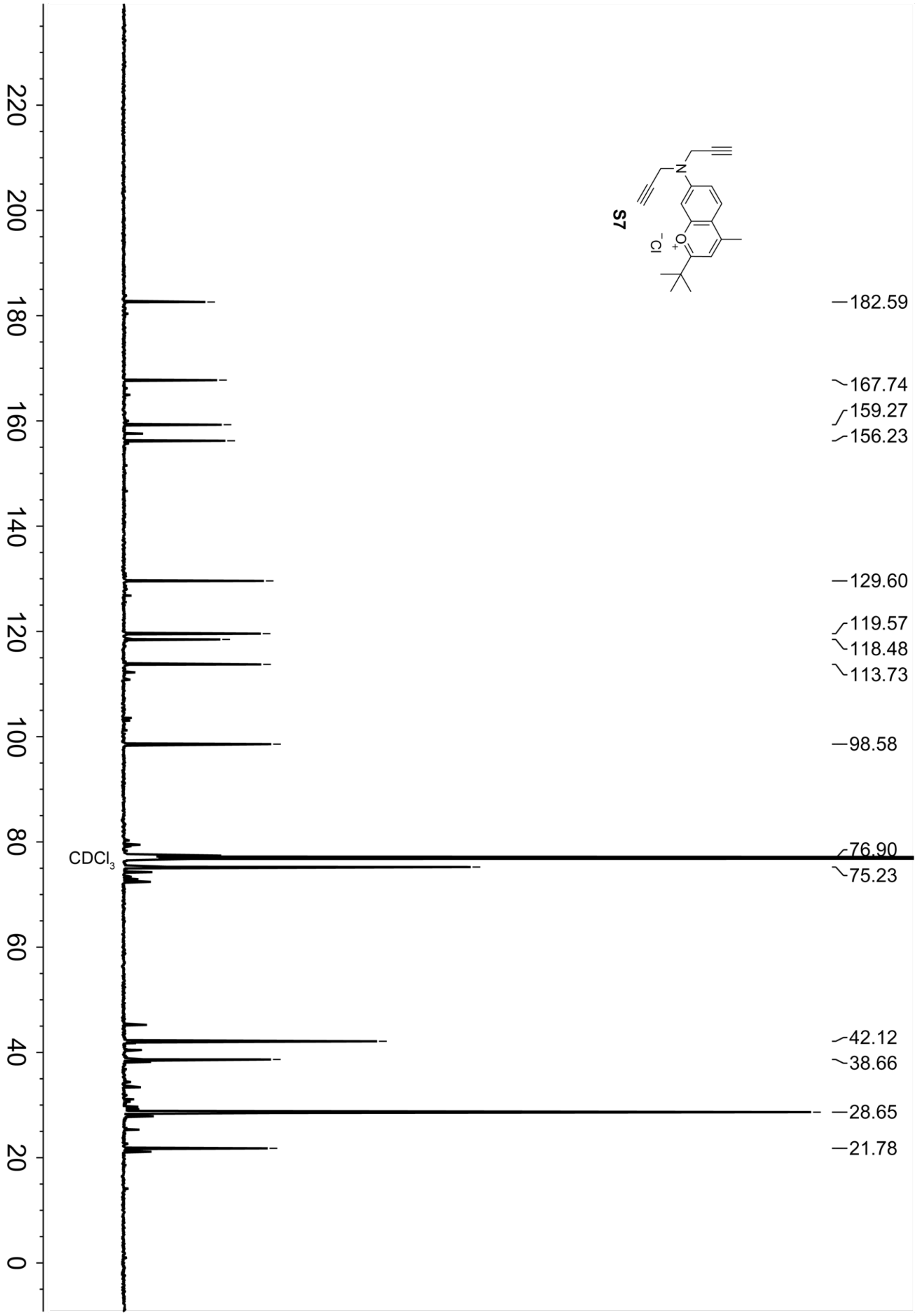

^{13}C NMR(126 MHz, $CDCl_3$) of **S7**.

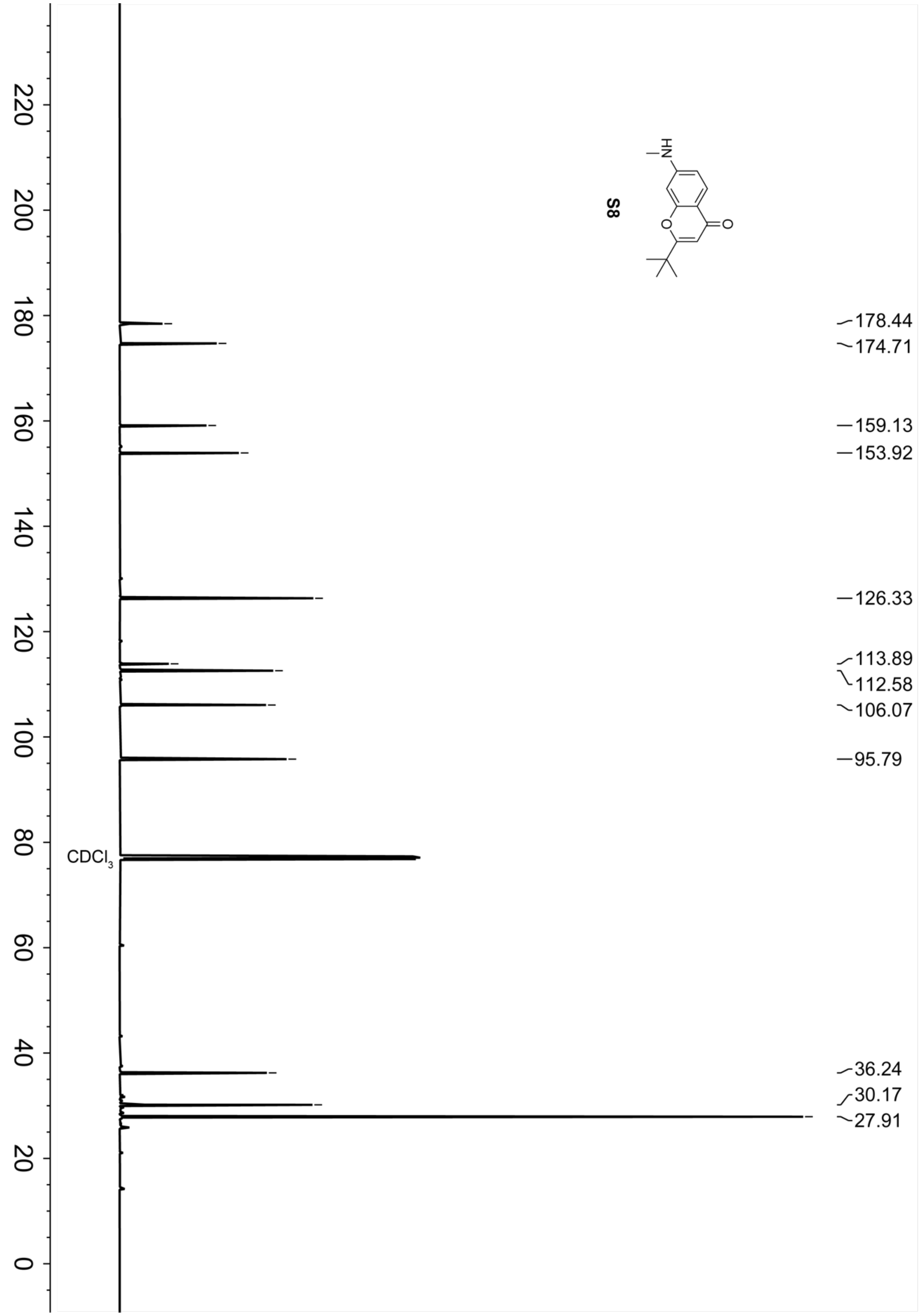

^{13}C NMR (126 MHz, $CDCl_3$) of **S8**.

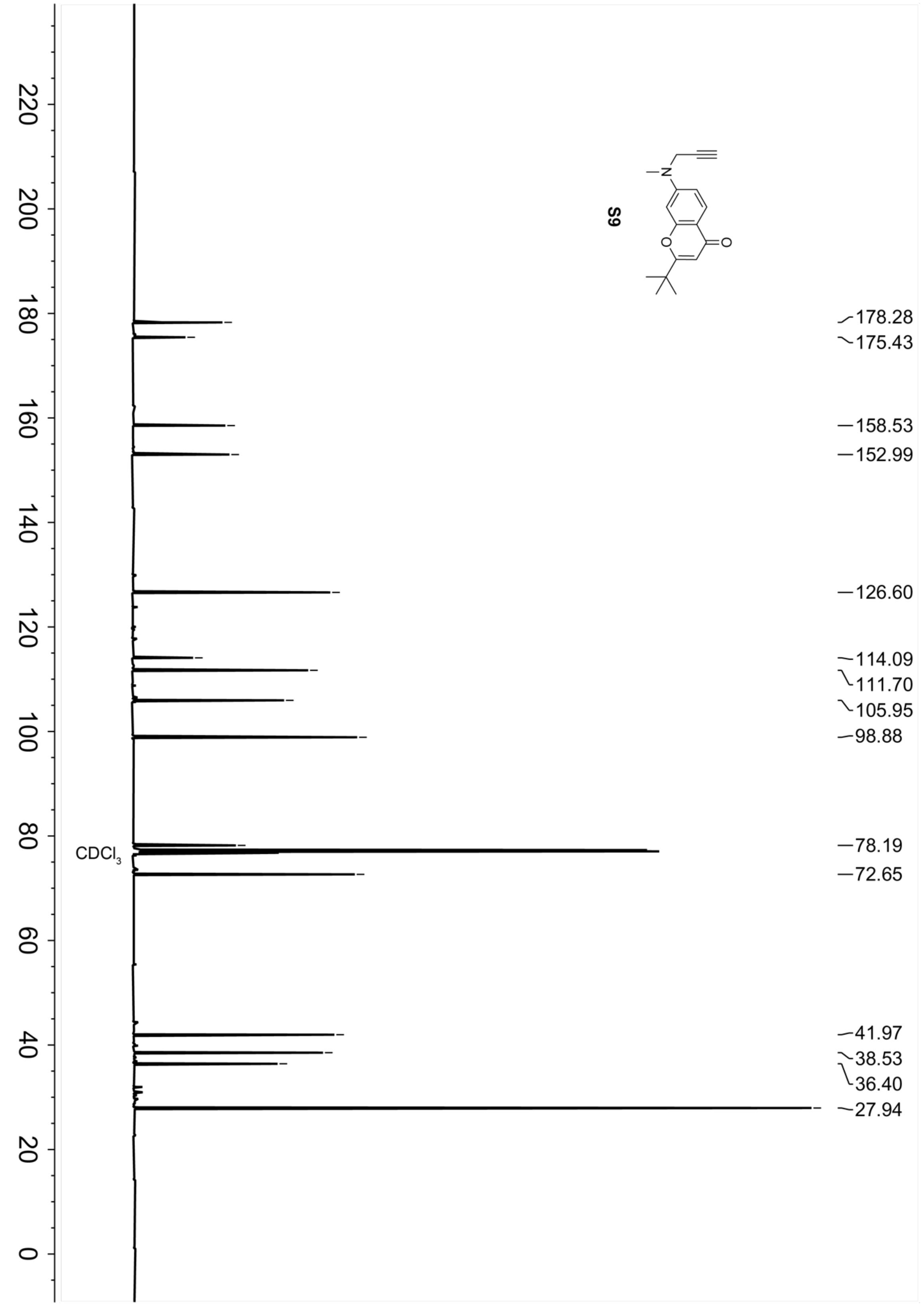

^{13}C NMR(126 MHz, $CDCl_3$) of **S9**.

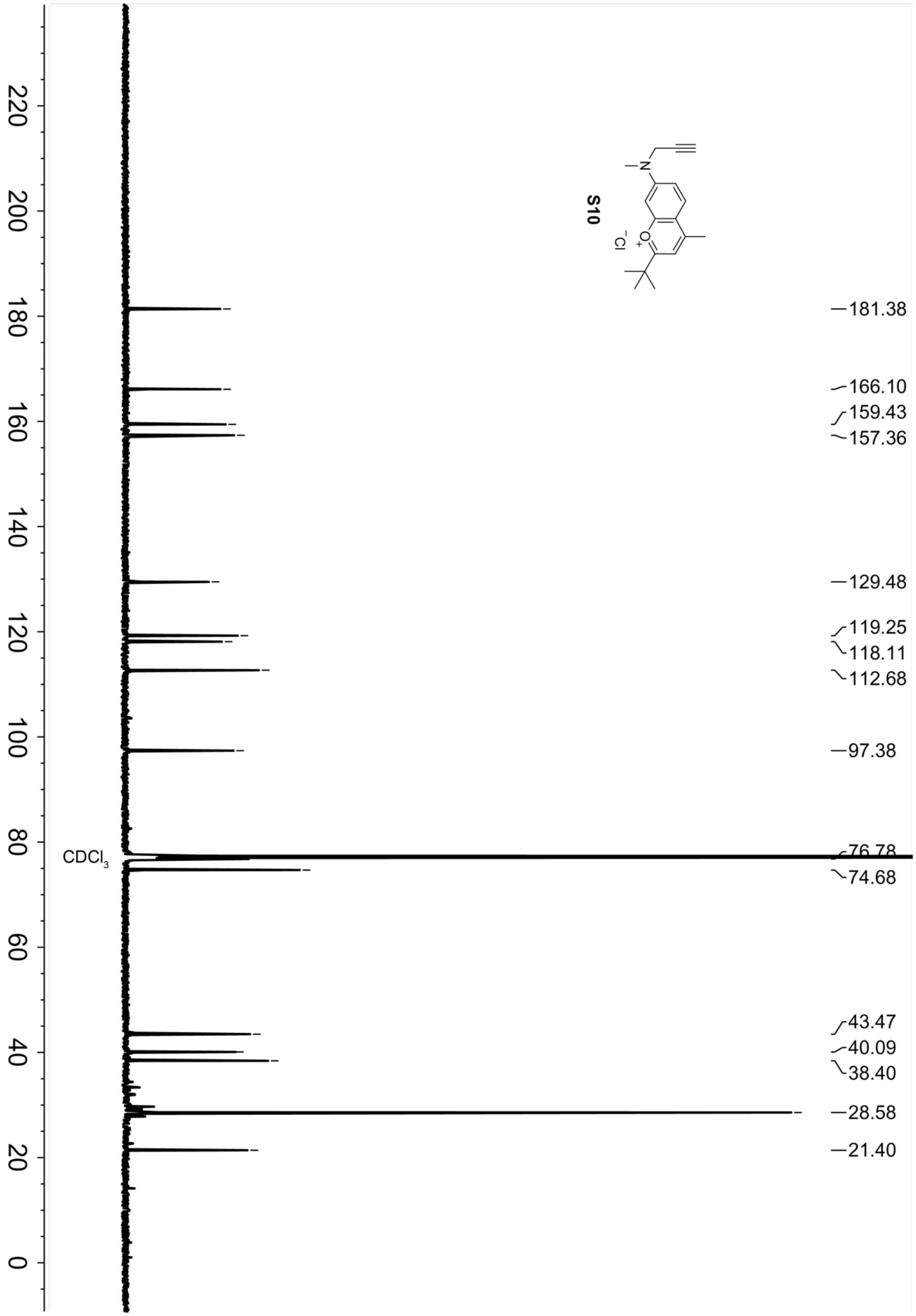

^{13}C NMR(126 MHz, $CDCl_3$) of **S10**.

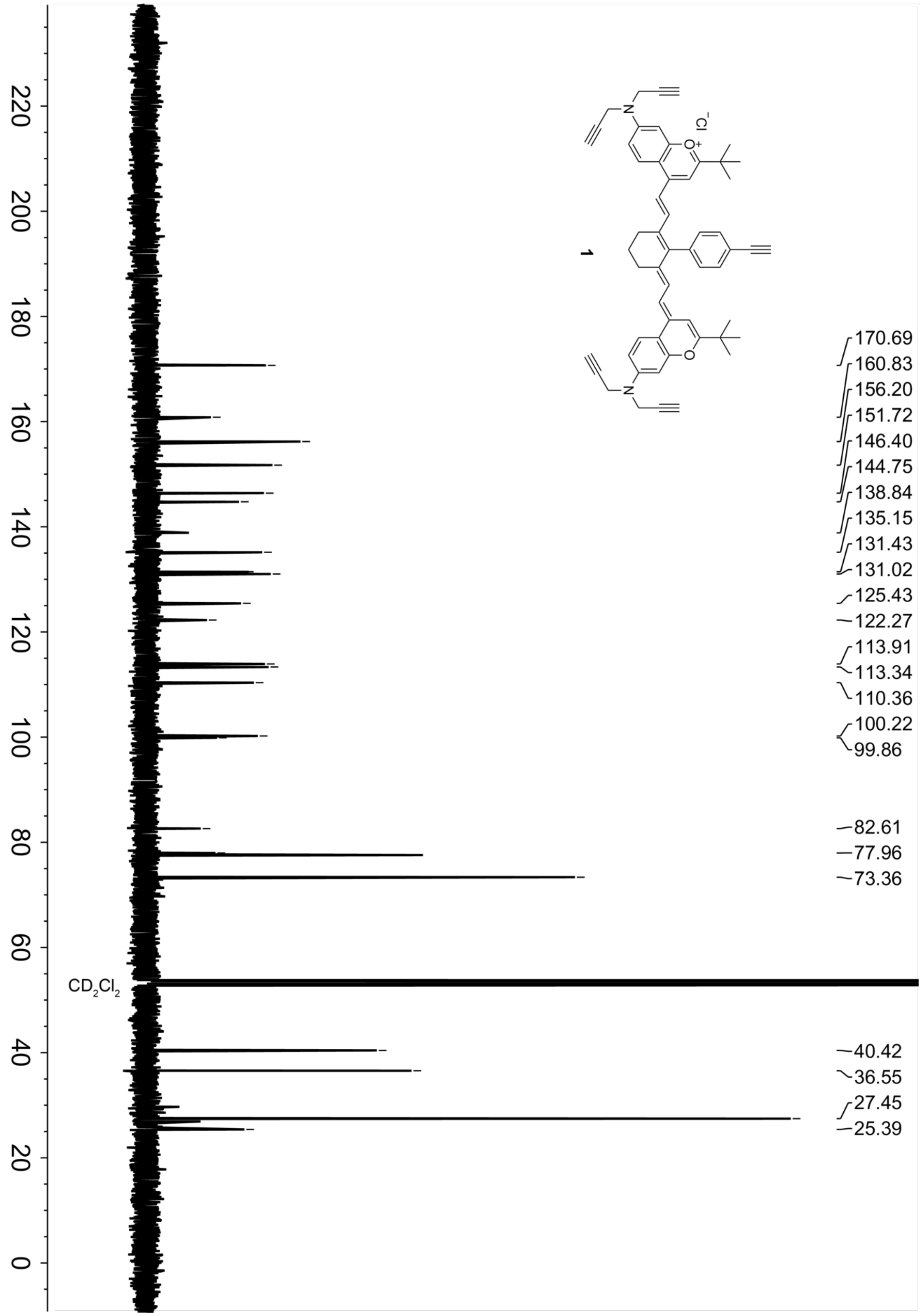

^{13}C NMR (126 MHz, CD_2Cl_2) of **1**.

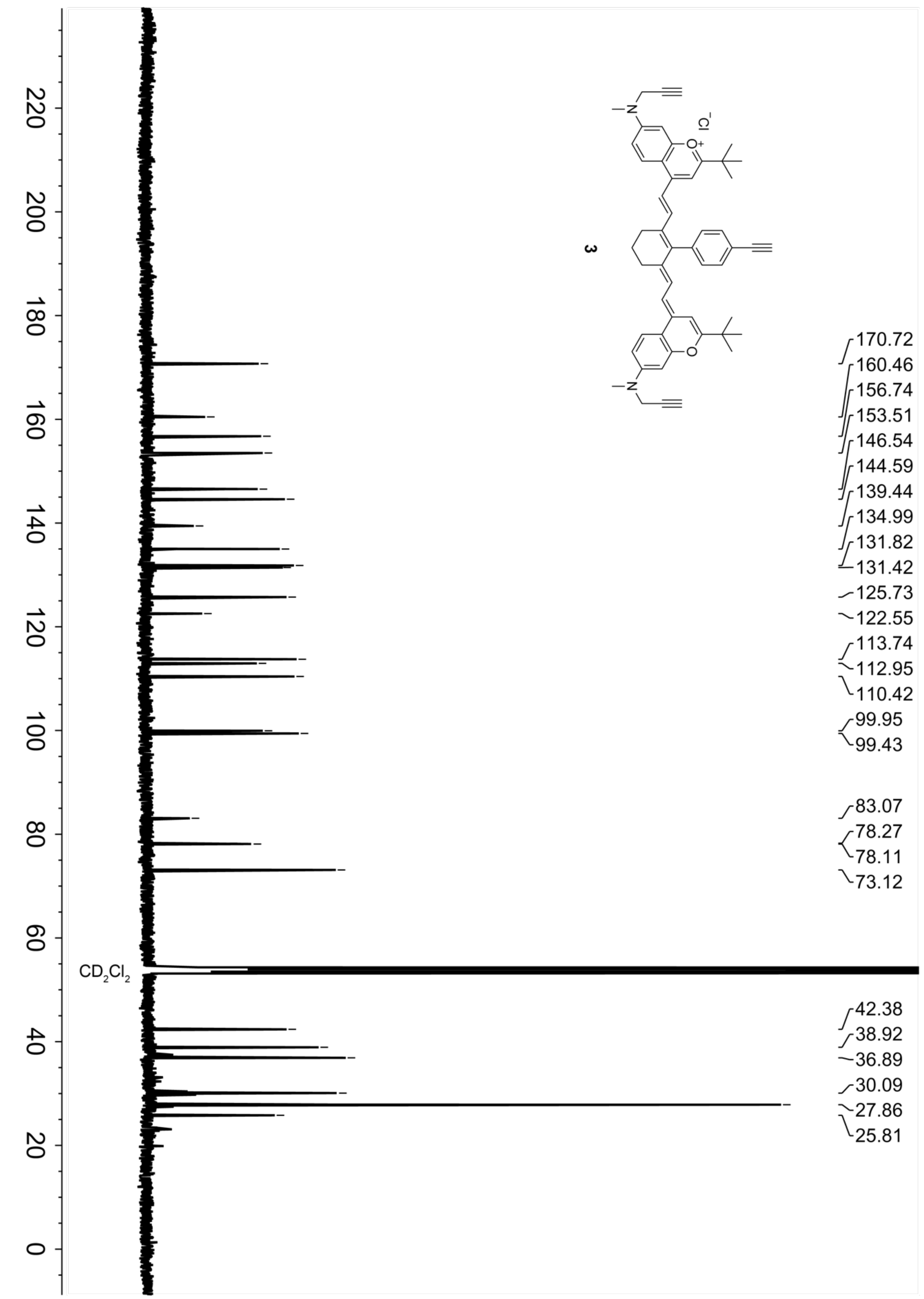

^{13}C NMR (126 MHz, CD_2Cl_2) of **3**.

GPC Spectra

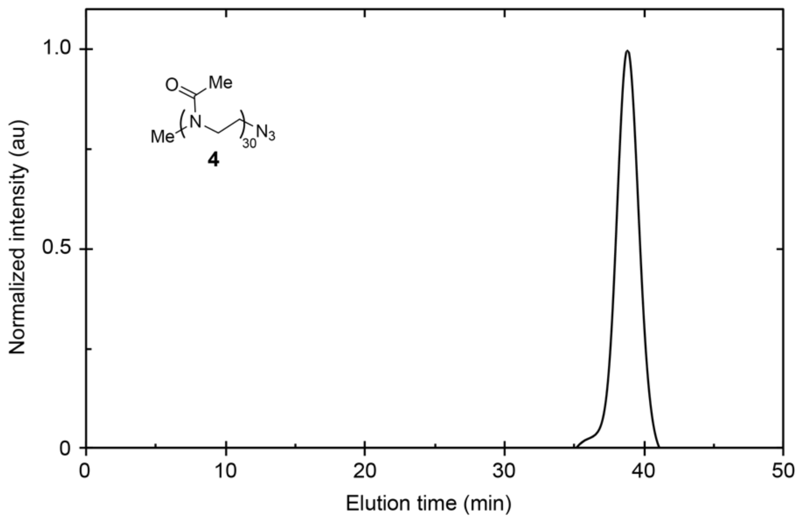

GPC spectrum of **4** (dUV 210 nm).

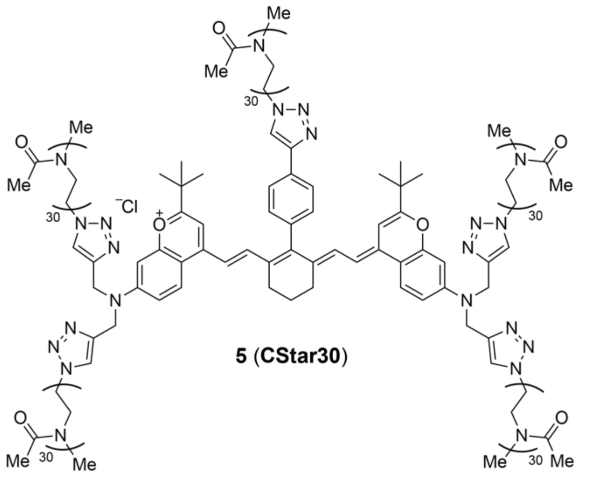

5 (**CStar30**)

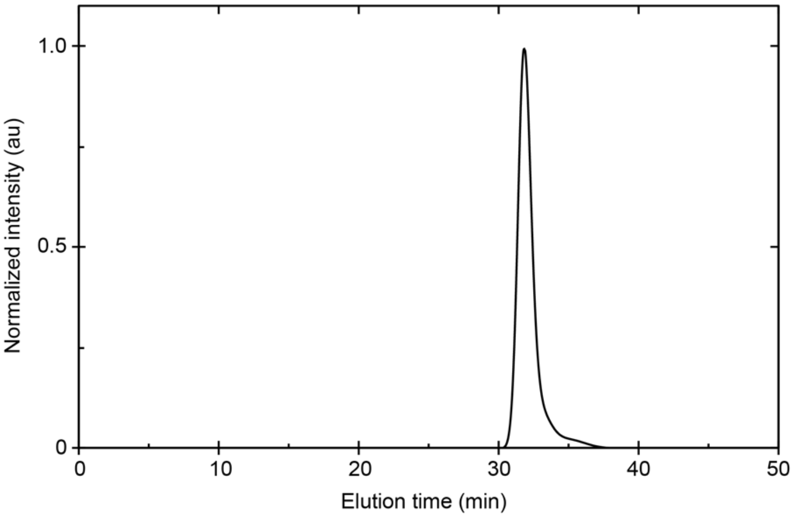

GPC spectrum of **5** (**CStar30**) (dUV 210 nm).

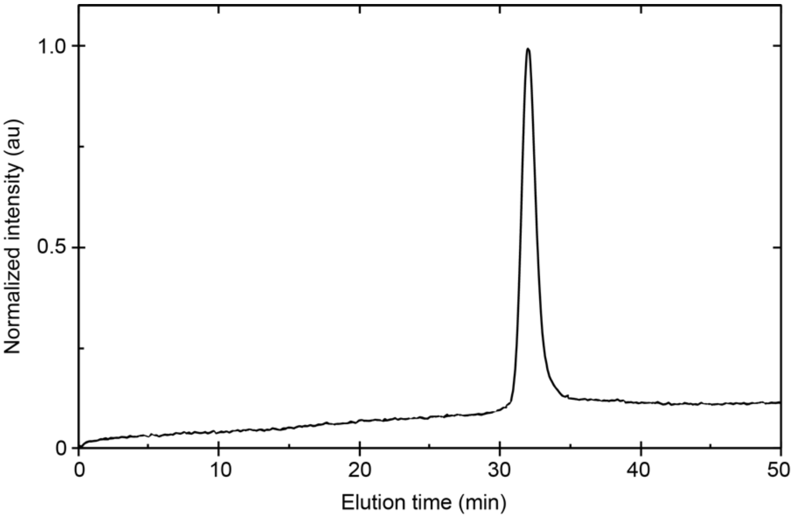

GPC spectrum of **5** (**CStar30**) (dUV 550 nm).

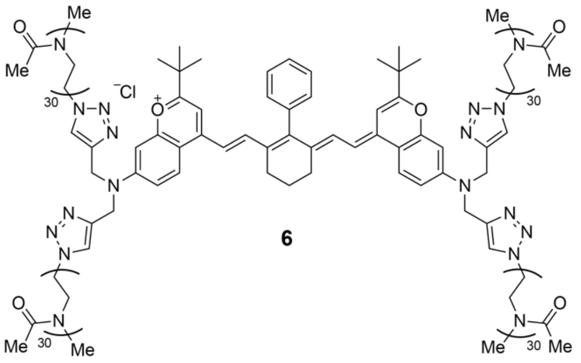

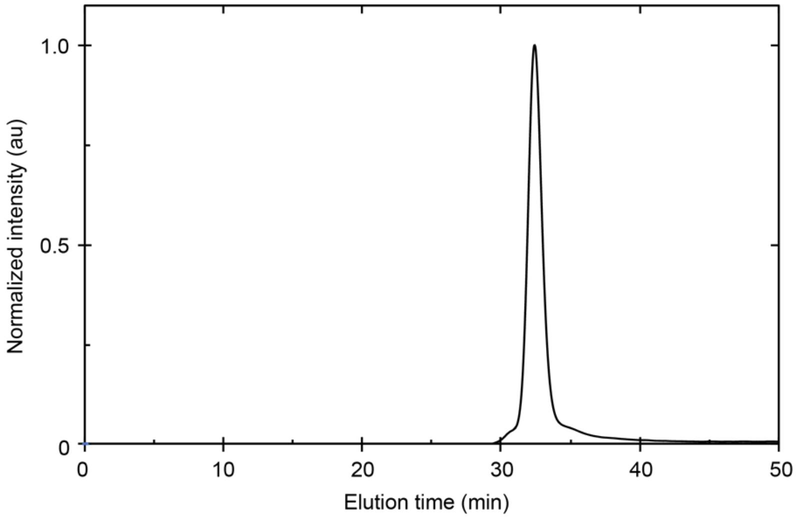

GPC spectrum of **6** (dUV 210 nm).

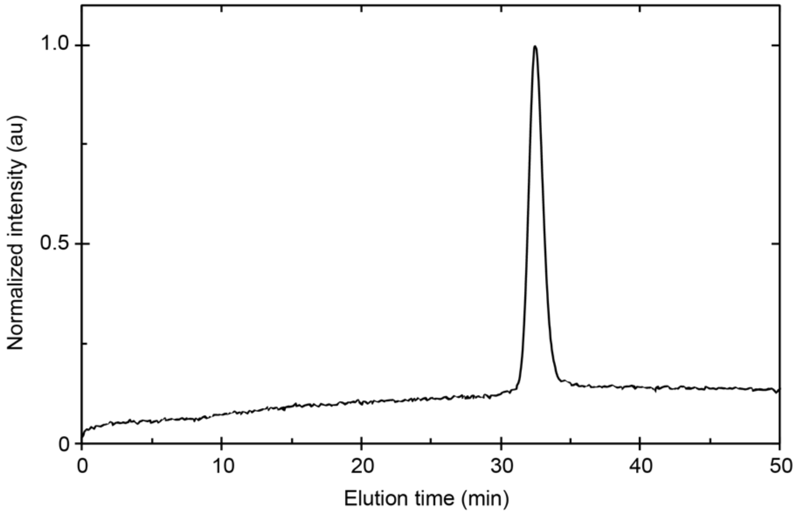

GPC spectrum of **6** (dUV 550 nm).

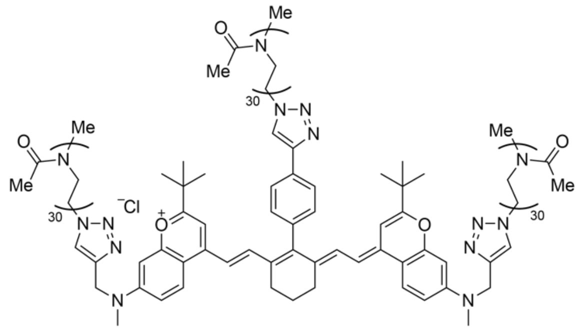

7

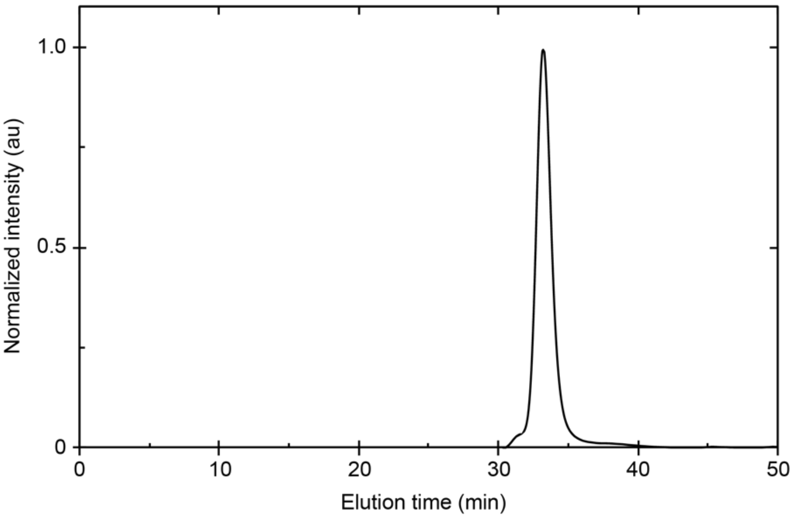

GPC spectrum of **7** (dUV 210 nm).

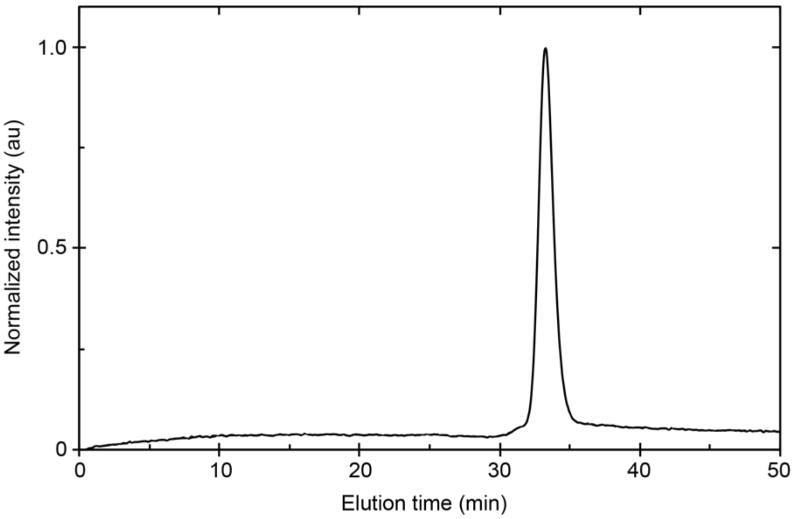

GPC spectrum of **7** (dUV 550 nm).

MALDI Spectra

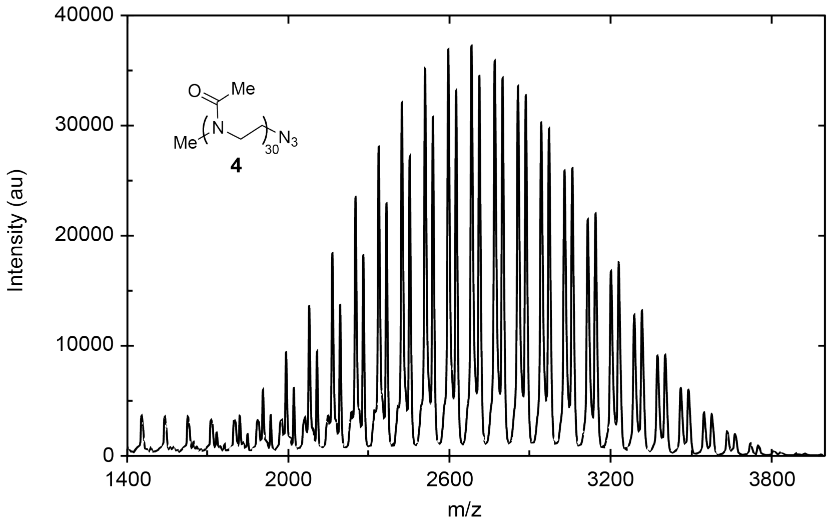

MALDI spectrum of **4**.

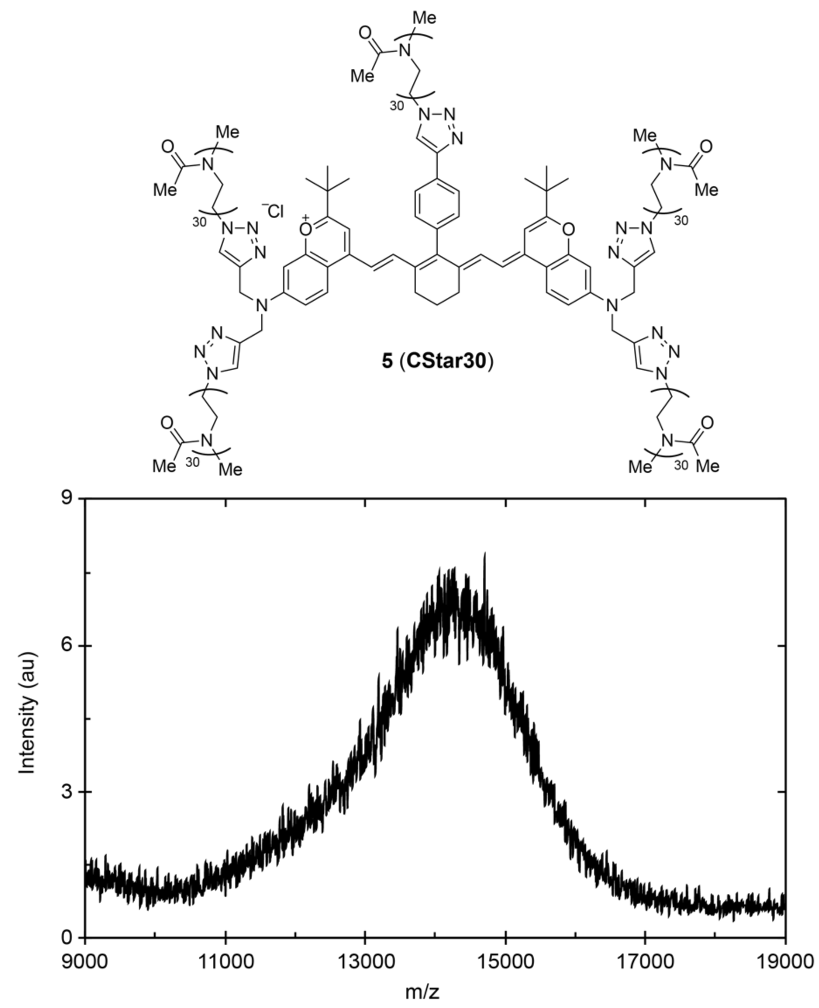

MALDI spectrum of **5** (**CStar30)**.

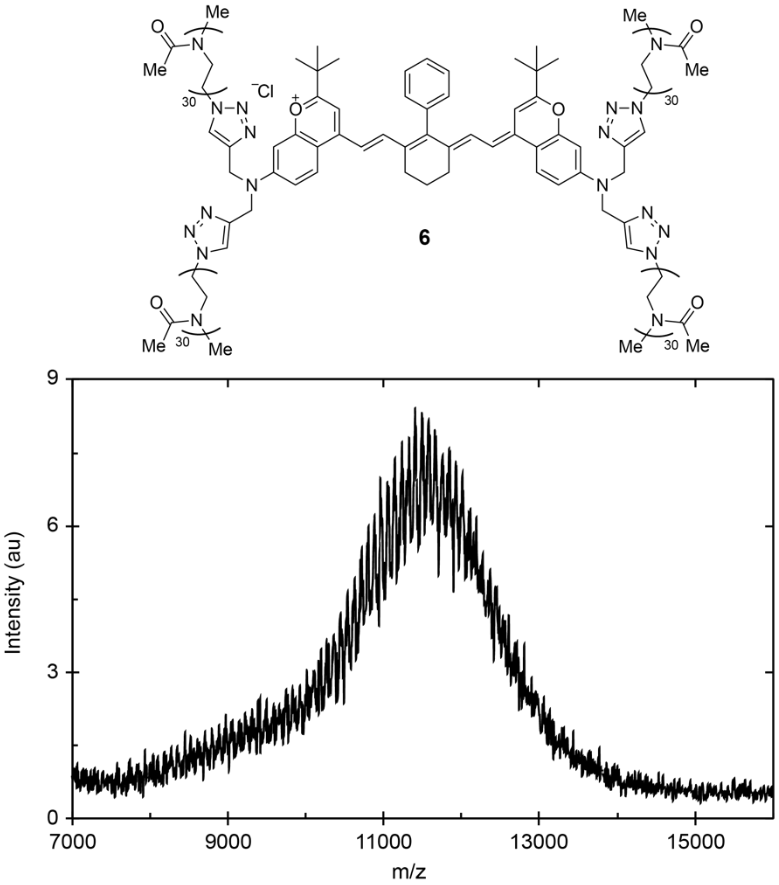

MALDI spectrum of **6**.

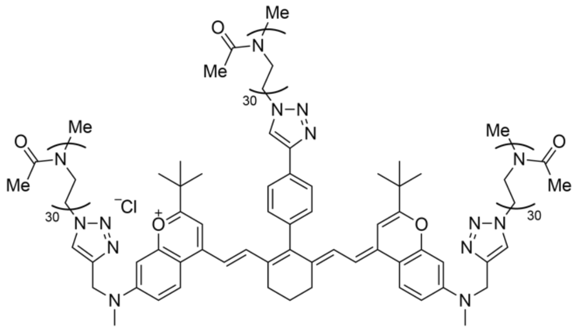

7

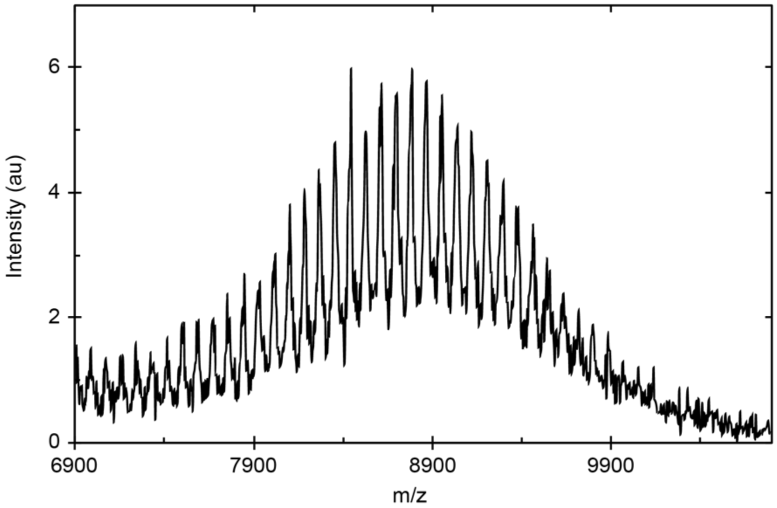

MALDI spectrum of **7**.

VII. Supporting References

1. Cosco, E.D.; Lim, I.; Sletten, E.M. "Photophysical Properties of Indocyanine Green in the Shortwave Infrared Region." *ChemPhotoChem*. **2021**, *5*, 727-734.
2. Jia, S.; Lin, E.Y.; Mobley, E.B.; Lim, I.; Guo, L.; Kallepu, S.; Low, P.S.; Sletten, E.M. "Water-Soluble Chromenylium Dyes for Shortwave Infrared Imaging in Mice." *Chem.* **2023**, *9* (12), 3648-3665.
3. (a) Knop, K.; Hoogenboom, R.; Fischer, D.; Schubert, U.S. "Poly(ethylene glycol) in Drug Discovery: Pros and Cons as Well as Potential Alternatives." *Angew. Chem., Int. Ed.* **2010**, *49* (36), 6288-6308. (b) Viegas, T.X.; Bentley, M.D.; Harris, J.M.; Fang, Z.; Yoon, K.; Dizman, B.; Weimer, R.; Mero, A.; Pasut, G.; Veronese, F.M. "Polyoxazoline: Chemistry, Properties, and Applications in Drug Delivery." *Bioconjugate Chem.* **2011**, *22* (5), 976-986. (c) Moreadith, R.W.; Viegas, T.X.; Bentley, M.D.; Harris, J.M.; Fang, Z.; Yoon, K.; Dizman, B.; Weimer, R.; Rae, B.P.; Li, Z.; Rader, C.; Standaert, D.; Olanow, W. "Clinical Development of a Poly(2-oxazoline) (POZ) Polymer Therapeutic for the Treatment of Parkinson's Disease – Proof of Concept of POZ as a Versatile Polymer Platform for Drug Development in Multiple Therapeutic Indications." *Eur. Polym. J.* **2017**, *88*, 524-552. (d) Verbraeken, B.; Monnery, B.D.; Lava, K.; Hoogenboom, R. "The Chemistry of Poly(2-oxazoline)s." *Eur. Polym. J.* **2017**, *88*, 451-469. (e) Yang, Q.; Jacobs, T.M.; McCallen, J.D.; Moore, D.T.; Huckaby, J.T.; Edelstein, J.N.; Lai, S.K. "Analysis of Pre-existing IgG and IgM Antibodies Against Polyethylene Glycol (PEG) in the General Population." *Anal. Chem.* **2016**, *88* (23), 11804-11812.
4. Cosco, E.D.; Spearman, A.L.; Ramakrishnan, S.; Lingg, J.G.P.; Saccomano, M.; Pengshung, M.; Arús, B.A.; Wong, K.C.Y.; Glasl, S.; Ntziachristos, V.; Warmer, M.; McLaughlin, R.R.; Bruns, O.T.; Sletten, E.M. "Shortwave Infrared Polymethine Fluorophores Matched to Excitation Lasers Enable Non-Invasive, Multicolour In Vivo Imaging in Real Time." *Nat. Chem.* **2020**, *12*, 1123-1130.
5. Cosco, E.D.; Arús, B.A.; Spearman, A.L.; Atallah, T.L.; Lim, I.; Leland, O.S.; Bischof, T.S.; Bruns, O.T.; Sletten, E.M. "Bright Chromenylium Polymethine Dyes Enable Fast, Four-Color In Vivo Imaging with Shortwave Infrared Detection." *J. Am. Chem. Soc.* **2021**, *143* (18), 6836-6846.
6. Ren, J.M.; McKenzie, T.G.; Fu, Q.; Wong, E.H.H.; Xu, J.; An, Z.; Shanmugam, S.; Davis, T.P.; Boyer, C.; Qiao, G.G. "Star Polymers." *Chem. Rev.* **2016**, *116*, 6743-6836.
7. Würth, C.; Grabolle, M.; Pauli, J.; Spieles, M.; Resch-Genger, U. "Relative and Absolute Determination of Fluorescence Quantum Yields of Transparent Samples." *Nat. Protocols.* **2013**, *8* (8), 1535-1550.
8. International Commission on Non-Ionizing Radiation Protection (ICNIRP). "ICNIRP Guidelines on Limits of Exposure to Laser Radiation of Wavelengths between 180 nm and 1,000 μm." *Health Phys.* **2013**, *105* (3), 271-295.
9. Schindelin, J.; Arganda-Carreras, I.; Frise, E.; Kaynig, V.; Longair, M.; Pietzsch, T.; Preibisch, S.; Rueden, C.; Saalfeld, S.; Schmid, B.; Tinevez, J.-Y.; White, D. J.; Hartenstein, V.; Eliceiri, K.; Tomancak, P.; Cardona, A. "Fiji: An Open-Source Platform for Biological-Image Analysis." *Nat. Methods.* **2012**, *9* (7), 676–682.
10. Schneider, C. A.; Rasband, W. S.; Eliceiri, K. W. "NIH Image to ImageJ: 25 Years of Image Analysis." *Nat. Methods.* **2012**, *9* (7), 671–675.

11. Okoh, O. A.; Bisby, R. H.; Lawrence, C. L.; Rolph, C. E.; Smith, R. B. "Promising Near-Infrared Non-Targeted Probes: Benzothiazole Heptamethine Cyanine Dyes." *J. Sulfur Chem*. **2014**, *35* (1), 42–56.
12. Hoogenboom, R.; Fijten, M. W. M.; Thijs, H. M. L.; Van Lankvelt, B. M.; Schubert, U. S. "Microwave-Assisted Synthesis and Properties of a Series of Poly(2-Alkyl-2-Oxazoline)s." *Des. Monomers Polym.* **2005**, *8* (6), 659-671.
13. Sedlacek, O.; Monnery, B.D.; Hoogenboom, R. "Synthesis of Defined High Molar Mass Poly(2-methyl-2-oxazoline)." *Polym. Chem.* **2019**, *10*, 1286-1290.

VIII. Appendix A

Single Channel Images for Excitation-Multiplexing Experiments

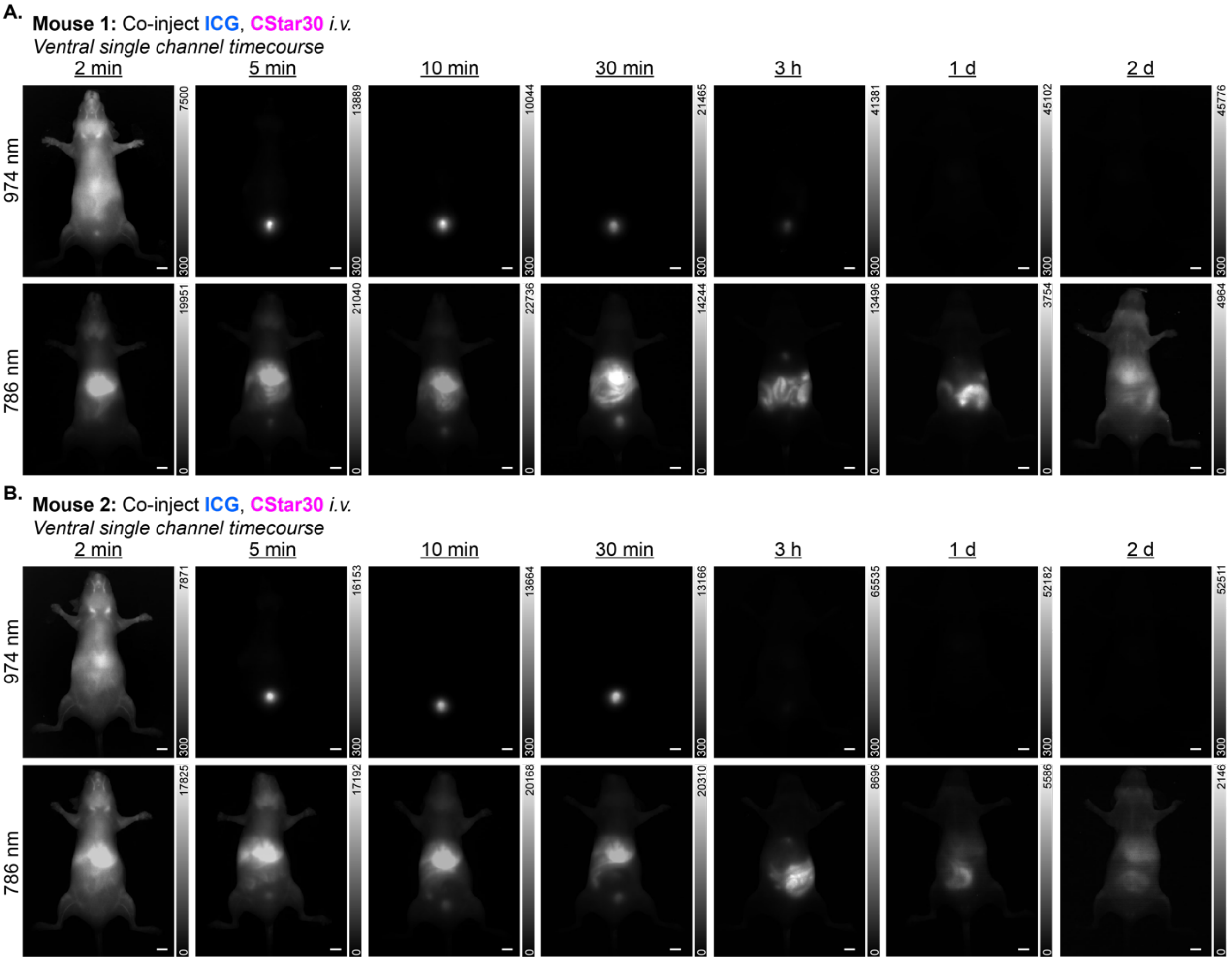

Figure SA1: Single channel imaging timecourse of **5** (**CStar30**) and **ICG** co-injected *i.v.* in mice for two-color excitation-multiplexed imaging. Ventral views of replicate "Mouse 1" (A) and "Mouse 2" (B) are displayed with either 974 nm (**CStar30**) or 786 nm (**ICG**) ex. All images corresponding to two-color timecourse in Figure S23B ("Mouse 1") and Figure S24B ("Mouse 2"). See Table S3 for sensitivity related parameters. Scale bars: 10 mm.

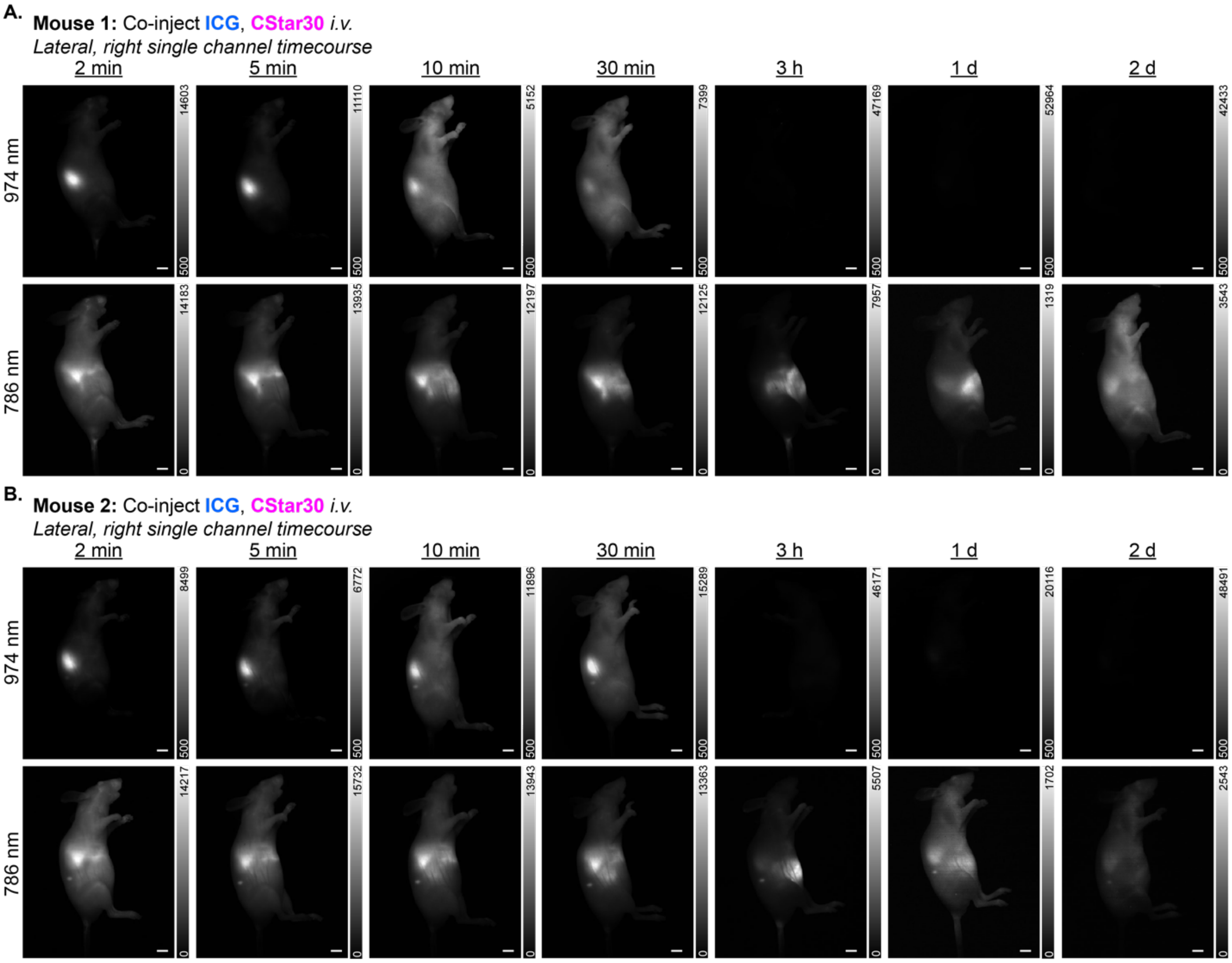

Figure SA2: Single channel imaging timecourse of **5** (**CStar30**) and **ICG** co-injected *i.v.* in mice for two-color excitation-multiplexed imaging. Lateral right views of replicate "Mouse 1" (A) and "Mouse 2" (B) are displayed with either 974 nm (**CStar30**) or 786 nm (**ICG**) ex. All images corresponding to two-color timecourse in Figure S23B ("Mouse 1") and Figure S24B ("Mouse 2"). See Table S3 for sensitivity related parameters. Scale bars: 10 mm.

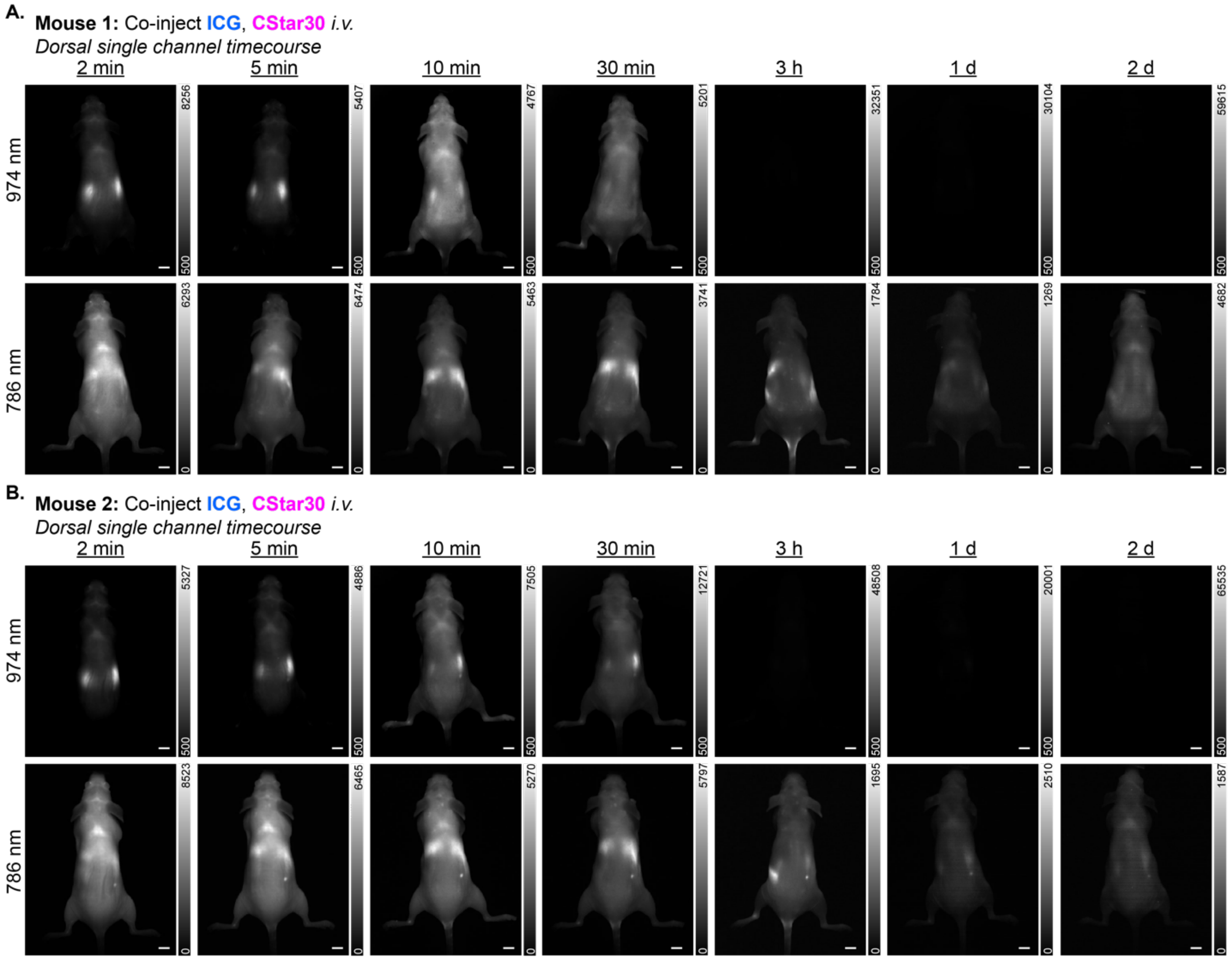

Figure SA3: Single channel imaging timecourse of **5** (**CStar30**) and **ICG** co-injected *i.v.* in mice for two-color excitation-multiplexed imaging. Dorsal views of replicate "Mouse 1" (A) and "Mouse 2" (B) are displayed with either 974 nm (**CStar30**) or 786 nm (**ICG**) ex. All images corresponding to two-color timecourse in Figure S23B ("Mouse 1") and Figure S24B ("Mouse 2"). See Table S3 for sensitivity related parameters. Scale bars: 10 mm.

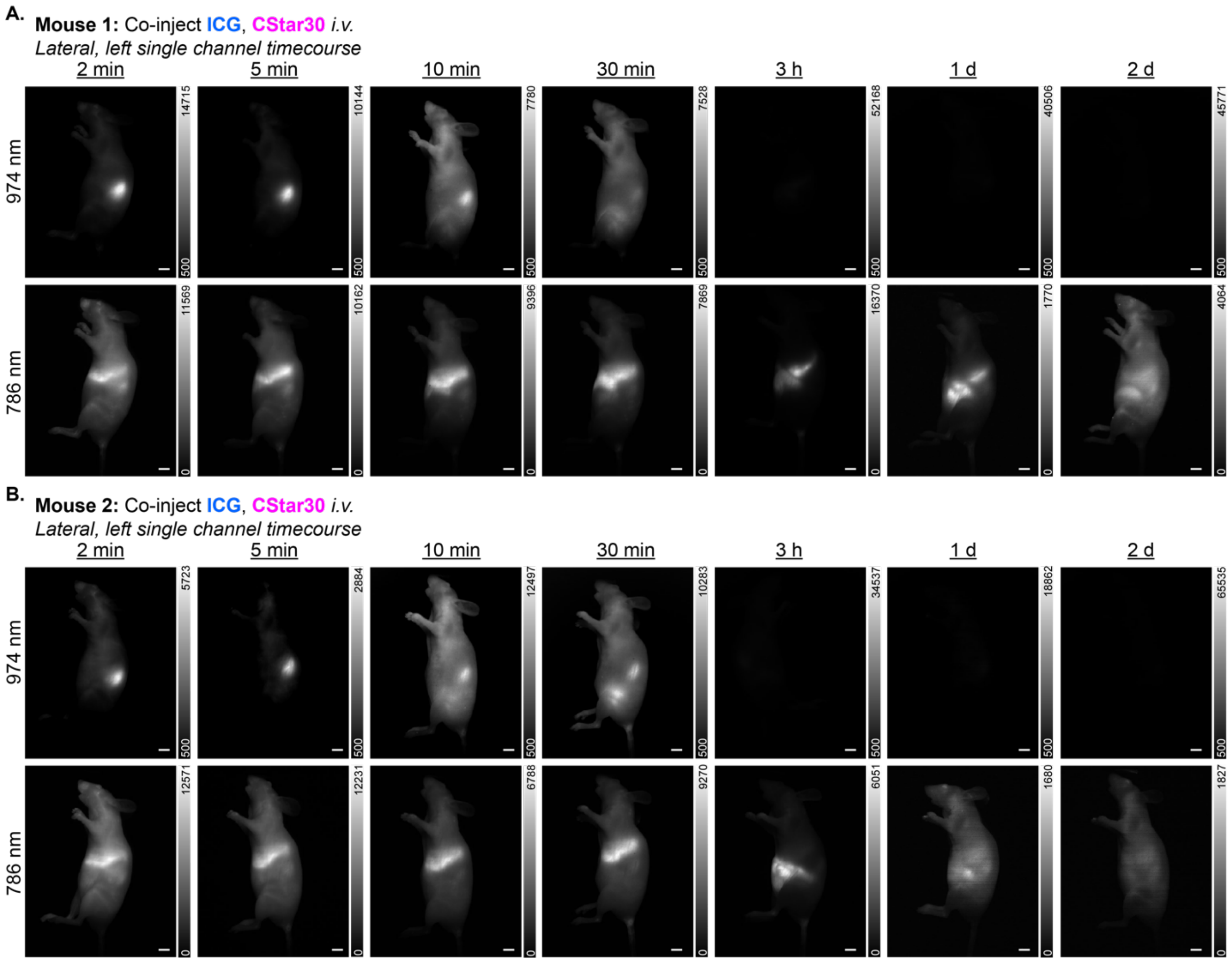

Figure SA4: Single channel imaging timecourse of **5** (**CStar30**) and **ICG** co-injected *i.v.* in mice for two-color excitation-multiplexed imaging. Lateral left views of replicate "Mouse 1" (A) and "Mouse 2" (B) are displayed with either 974 nm (**CStar30**) or 786 nm (**ICG**) ex. All images corresponding to two-color timecourse in Figure S23B ("Mouse 1") and Figure S24B ("Mouse 2"). See Table S3 for sensitivity related parameters. Scale bars: 10 mm.

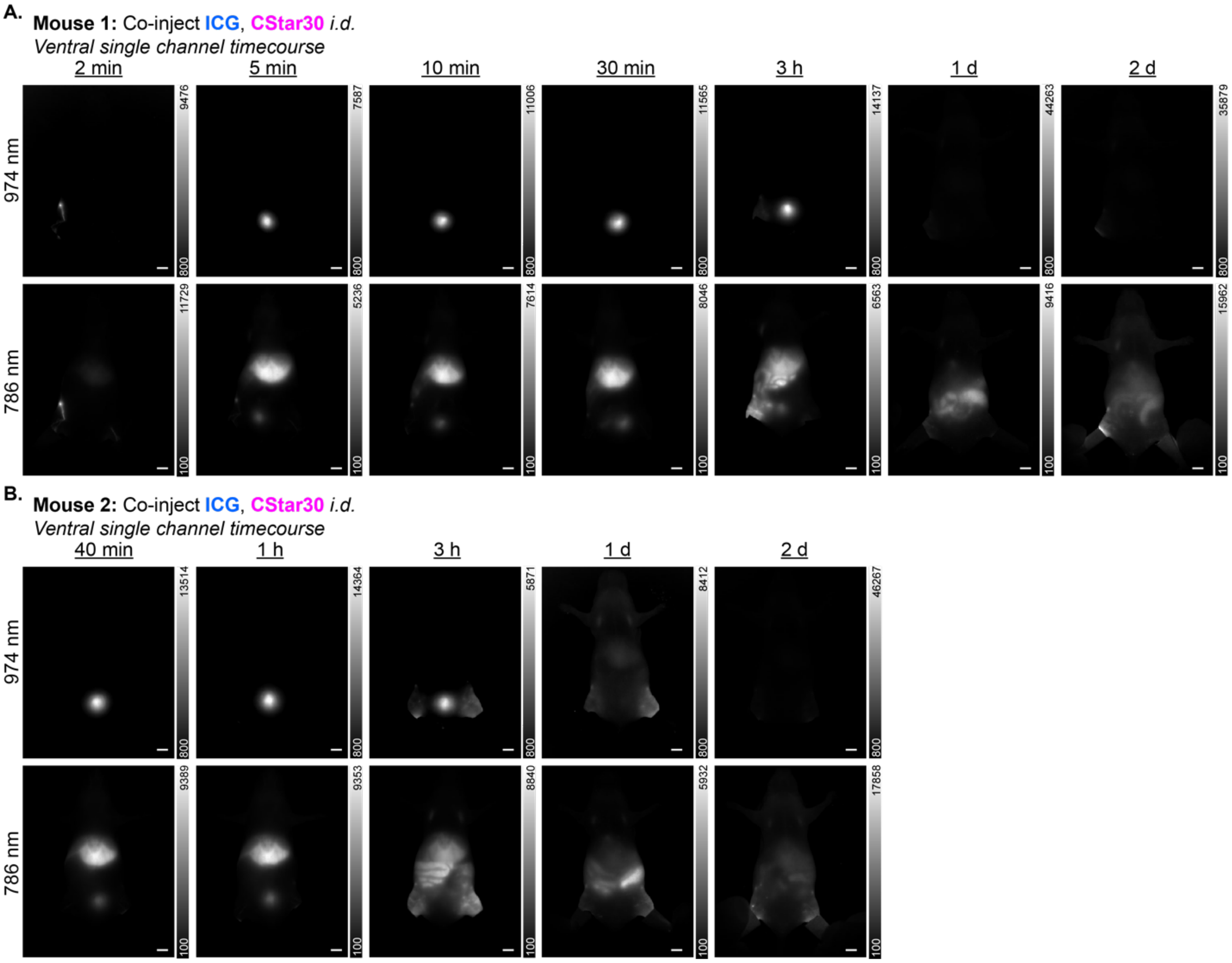

Figure SA5: Single channel imaging timecourse of **5** (**CStar30**) and **ICG** co-injected *i.d.* in mice for two-color excitation-multiplexed imaging. Ventral views of replicate "Mouse 1" (A) and "Mouse 2" (B) are displayed with either 974 nm (**CStar30**) or 786 nm (**ICG**) ex. All images corresponding to two-color timecourse in Figure S26B ("Mouse 1") and Figure S27B ("Mouse 2"). See Table S3 for sensitivity related parameters. Scale bars: 10 mm.

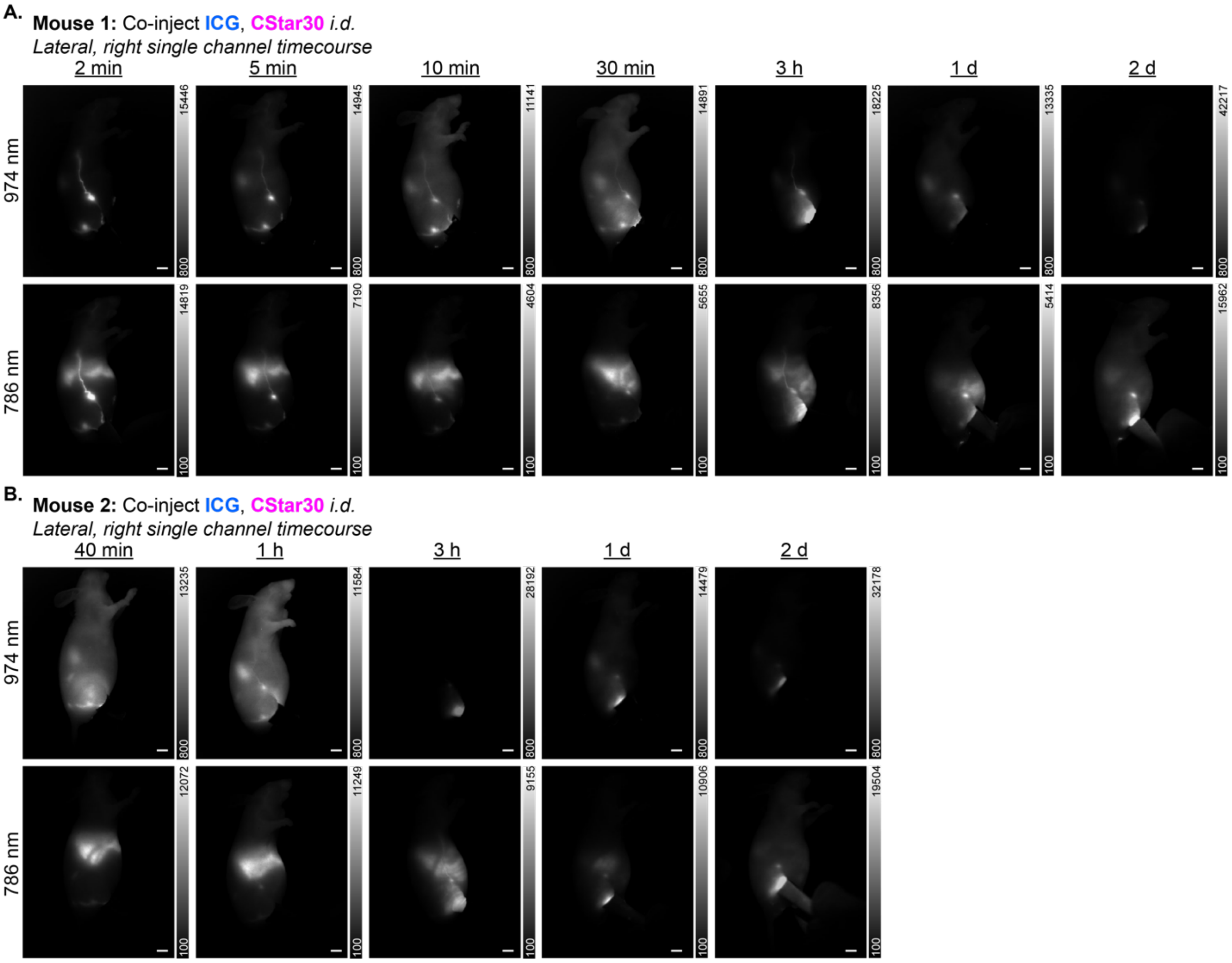

Figure SA6: Single channel imaging timecourse of **5** (**CStar30**) and **ICG** co-injected *i.d.* in mice for two-color excitation-multiplexed imaging. Lateral right views of replicate "Mouse 1" (A) and "Mouse 2" (B) are displayed with either 974 nm (**CStar30**) or 786 nm (**ICG**) ex. All images corresponding to two-color timecourse in Figure S26B ("Mouse 1") and Figure S27B ("Mouse 2"). See Table S3 for sensitivity related parameters. Scale bars: 10 mm.

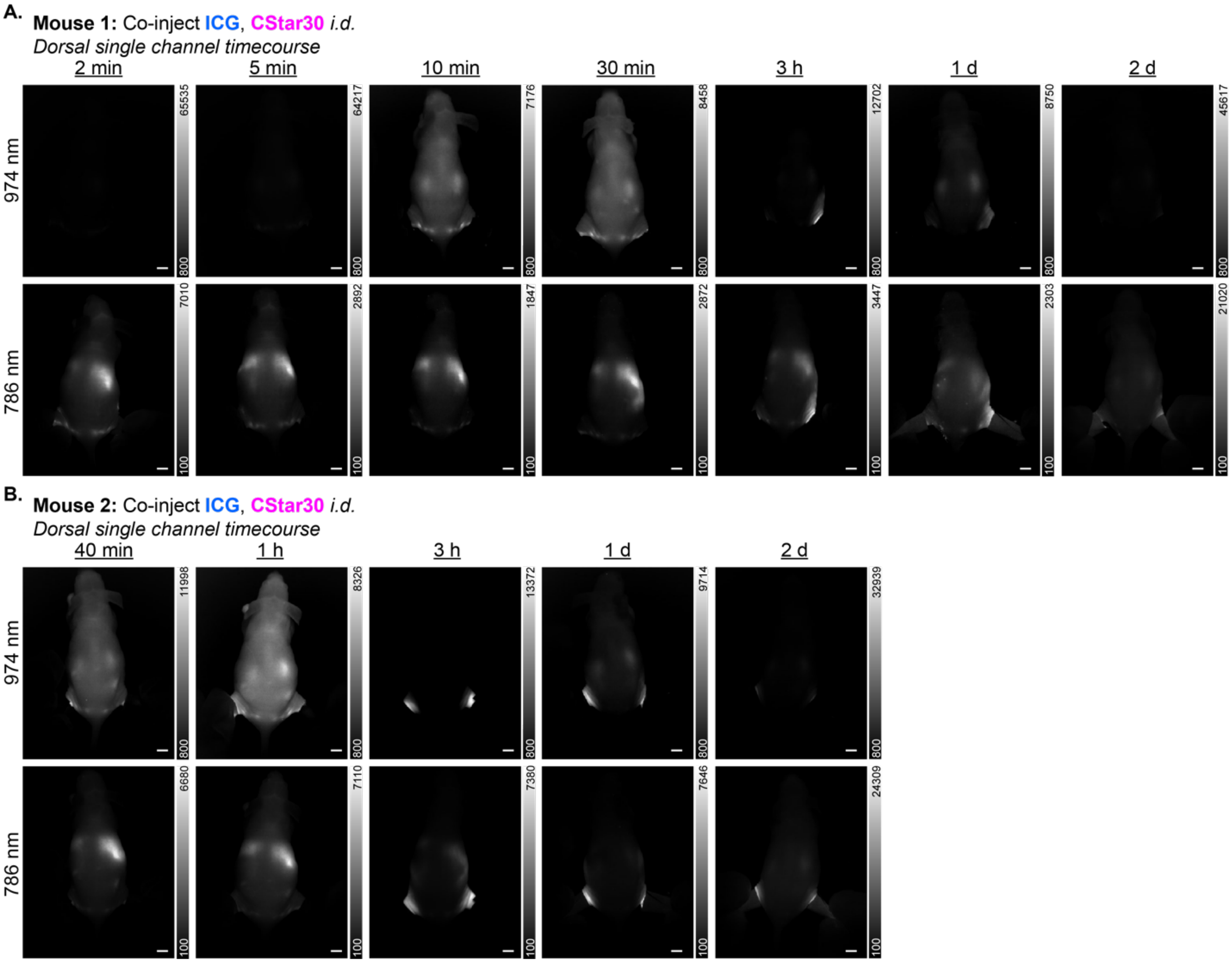

Figure SA7: Single channel imaging timecourse of **5** (**CStar30**) and **ICG** co-injected *i.d.* in mice for two-color excitation-multiplexed imaging. Dorsal views of replicate "Mouse 1" (A) and "Mouse 2" (B) are displayed with either 974 nm (**CStar30**) or 786 nm (**ICG**) ex. All images corresponding to two-color timecourse in Figure S26B ("Mouse 1") and Figure S27B ("Mouse 2"). See Table S3 for sensitivity related parameters. Scale bars: 10 mm.

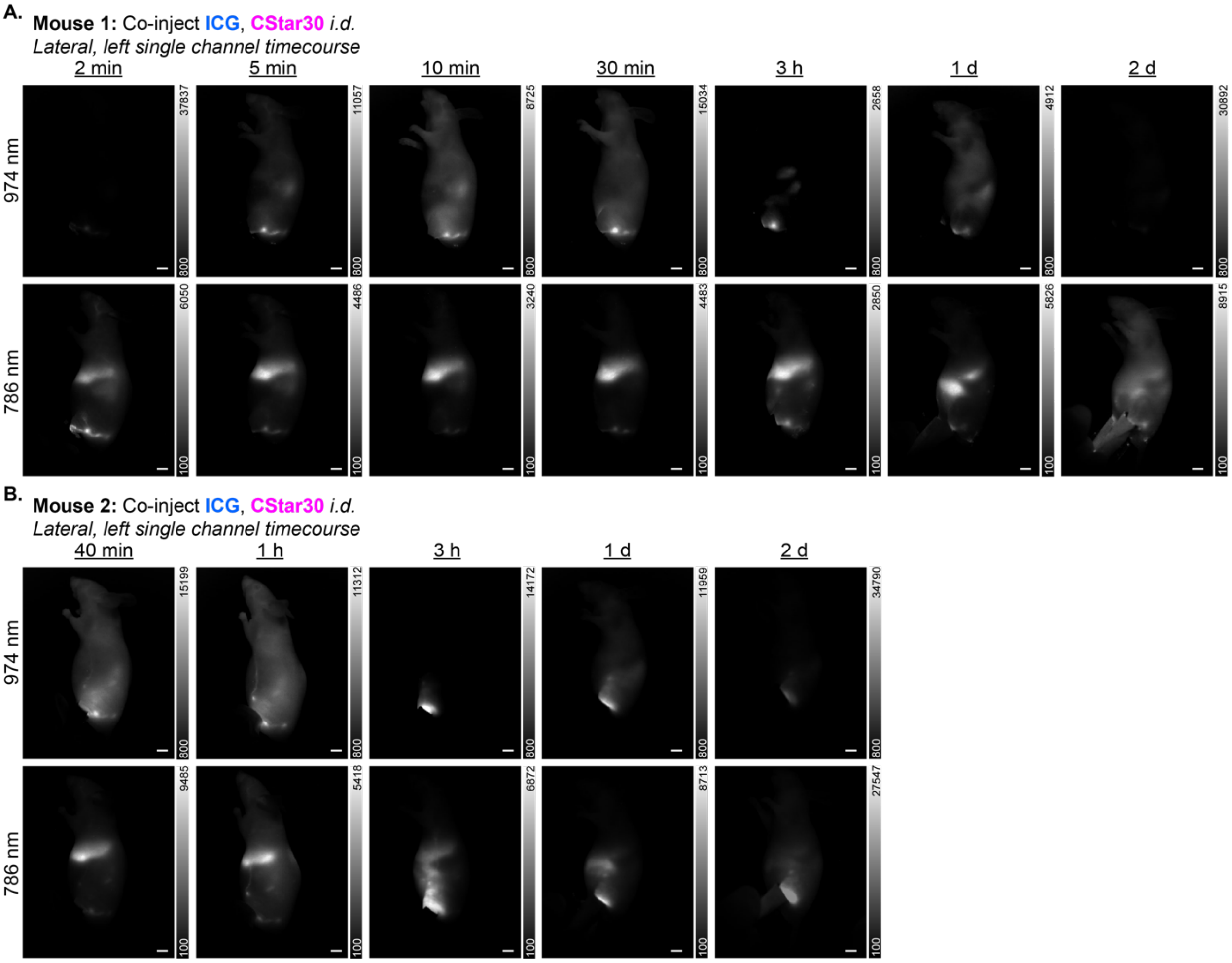

Figure SA8: Single channel imaging timecourse of **5** (**CStar30**) and **ICG** co-injected *i.d.* in mice for two-color excitation-multiplexed imaging. Lateral left views of replicate "Mouse 1" (A) and "Mouse 2" (B) are displayed with either 974 nm (**CStar30**) or 786 nm (**ICG**) ex. All images corresponding to two-color timecourse in Figure S26B ("Mouse 1") and Figure S27B ("Mouse 2"). See Table S3 for sensitivity related parameters. Scale bars: 10 mm.

IX. Appendix B

GPC Calibration via Poly(2-methyl-2-oxazoline) (POx) Polymers

As there are currently no commercially available POx polymer size standards for GPC, molecular weight calibration for GPC (see "Synthetic instrumentation") was performed with custom methyl-poly(2-methyl-2-oxazoline)-azide standards ranging 1–6 kDa. Termination with sodium azide was chosen because it has well-established procedures with quantitative end-capping. Characterization was done via matrix-assisted laser desorption ionization time of flight mass spectrometry (MALDI, see "Synthetic instrumentation") to obtain M_n, M_w and dispersity ($Đ$) values.

General polymerization procedure

These polymerization procedures were adapted based on previously reports.[1] To a flame-dried microwave vial under N_2, acetonitrile (4 M, anhydrous), and 2-methyl-2-oxazoline (10, 23, 47, or 70 equiv.) were added. Methyl triflate (1 equiv.) initiator was then added and the mixture was heated at 140 ˚C under microwave irradiation to 98% monomer consumption. The reaction mixture was allowed to cool to 25 ˚C and sodium azide (10 equiv.) was added to terminate the polymerization. The slurry was allowed to stir for 3 h at 40 ˚C under N_2, and was then filtered over celite to remove excess azide salts. The filtrate was concentrated under vacuum, redissolved in either chloroform or dichloromethane, and then precipitated into ice cold diethyl ether three times. For polymers with target molecular weights above 1 kDa, dialysis against water with the appropriate molecular weight cutoff membrane was subsequently performed for a minimum of 24 h. The resulting polymer was then dried under vacuum to yield a hygroscopic white, fluffy power. *Note*: NaN_3 presents an unusually high safety concern. To mitigate this, NaN_3-containing vessels were handled carefully under a dry, inert atmosphere (away from metals and acid) until removal of excess azide salts (disposed as a basic, aqueous hazardous waste solution).

MALDI sample preparation

Polymers were prepared for MALDI in 50:5:1 mixtures of 20 mg/mL *trans*-2-[3-(4-*tert*-butylphenyl)-2-methyl-2-propenylidene]malononitrile (DCTB) matrix in chloroform, 20 mg/mL polymer in chloroform, and 10 mg/mL sodium trifluoroacetate in isopropyl alcohol. Mixtures were deposited onto the target plate via dry drop.

Table SB1: MALDI values used as calibrants for Me-P(MeOx)$_n$-N_3 (POx) standards.

Methyl-poly(2-methyl-2-oxazoline)-azide (POx) GPC standards:

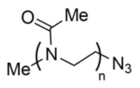

Target molecular weight (Da)	Actual degree of polymerization	MALDI M_n (Da)	MALDI M_w (Da)	$Đ$ (M_w/M_n)
approx. 1,000	n = 10	901	956	1.06
approx. 2,000	n = 28	2,427	2,616	1.08
approx. 4,000	n = 44	3,834	4,119	1.07
approx. 6,000	n = 57	4,932	5,285	1.07

MALDI spectra for POx standards
Note: 85 m/z: 2-methyl-2-oxazoline monomer spacing. 28 m/z: loss of N_2 (characteristic of azide ionization).

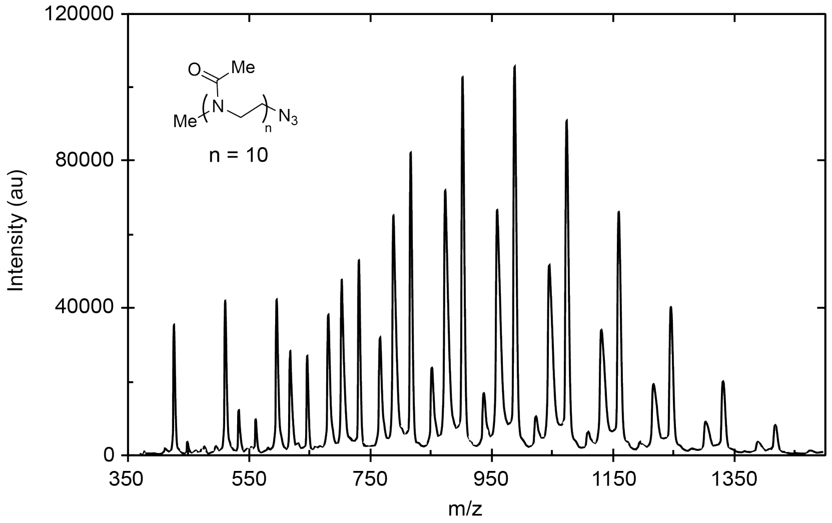

MALDI spectrum of POx, n = 10.

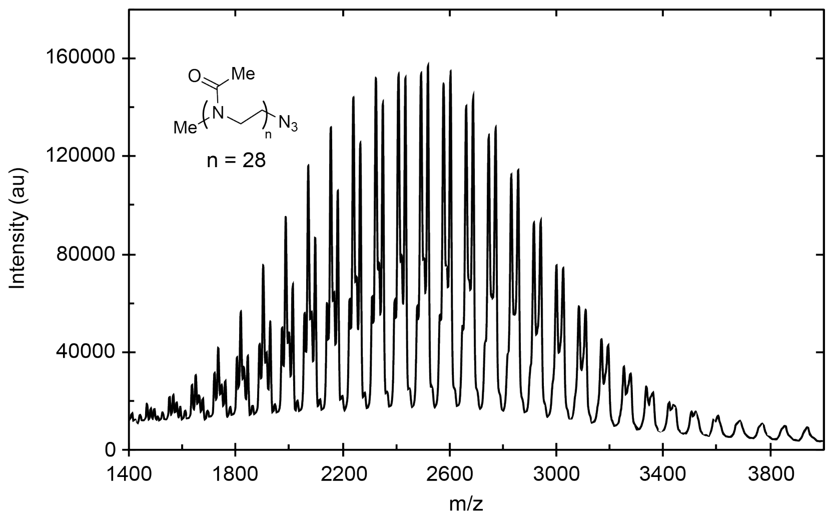

MALDI spectrum of POx, n = 28.

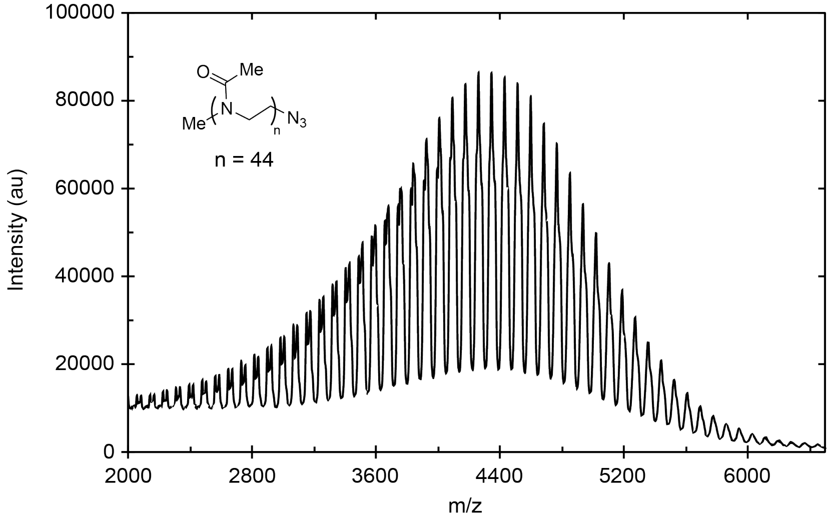

MALDI spectrum of POx, n = 44.

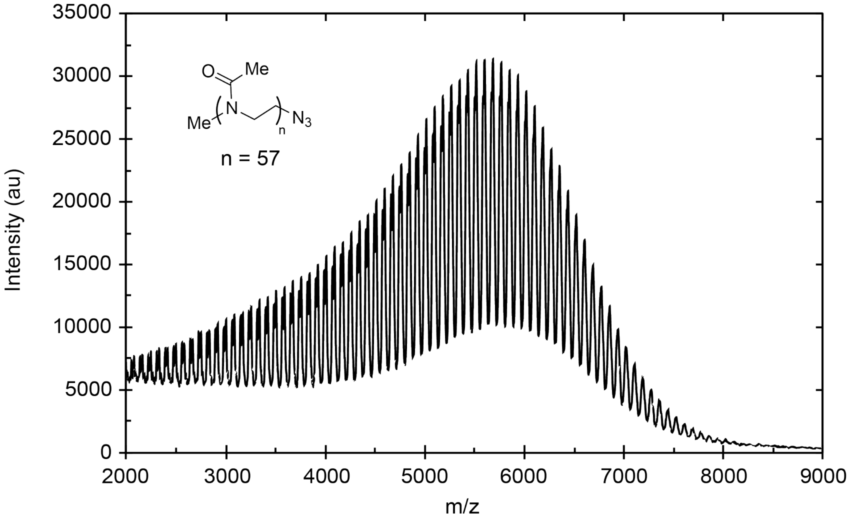

MALDI spectrum of POx, n = 57.

GPC spectra for POx standards

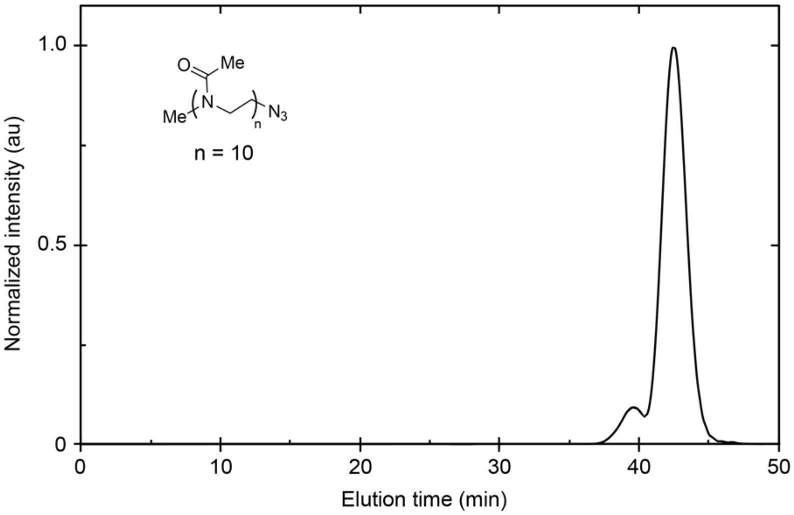

GPC spectrum of POx, n = 10 (dUV 210 nm).

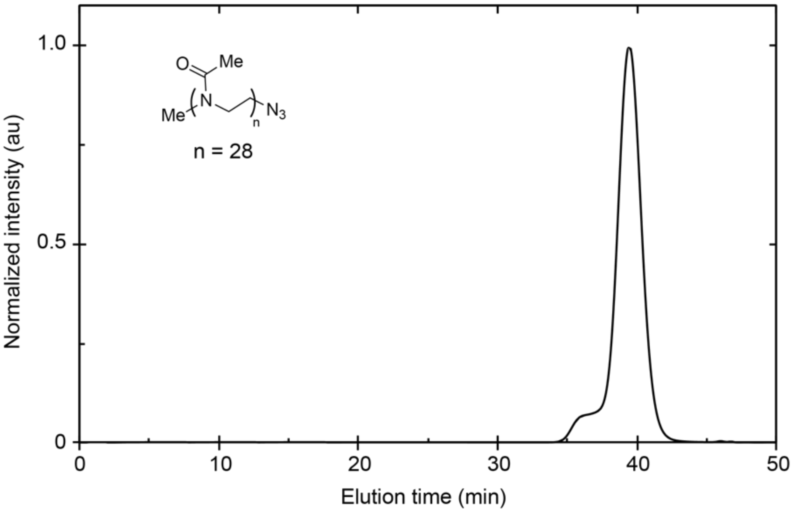

GPC spectrum of POx, n = 28 (dUV 210 nm).

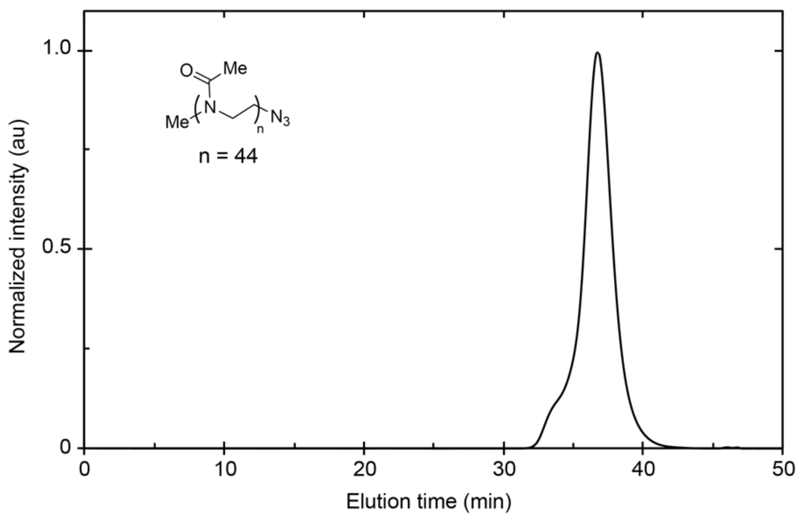

GPC spectrum of POx, n = 44 (dUV 210 nm).

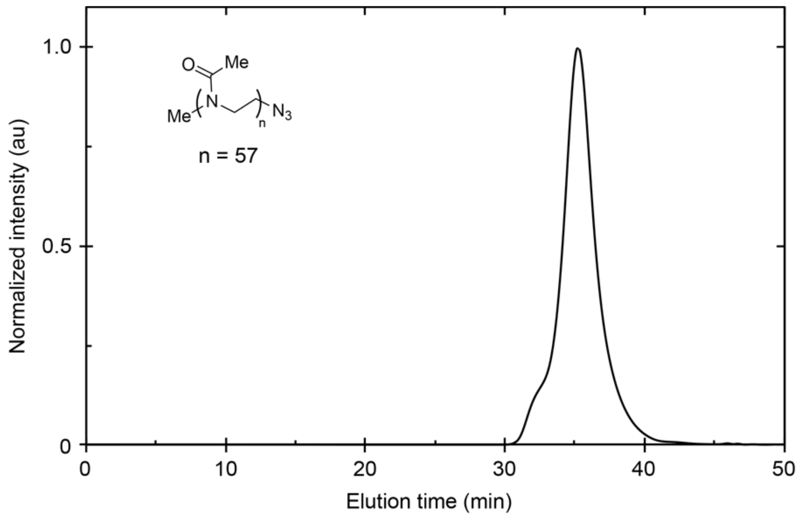

GPC spectrum of POx, n = 57 (dUV 210 nm).

^{1}H NMR spectra for POx standards

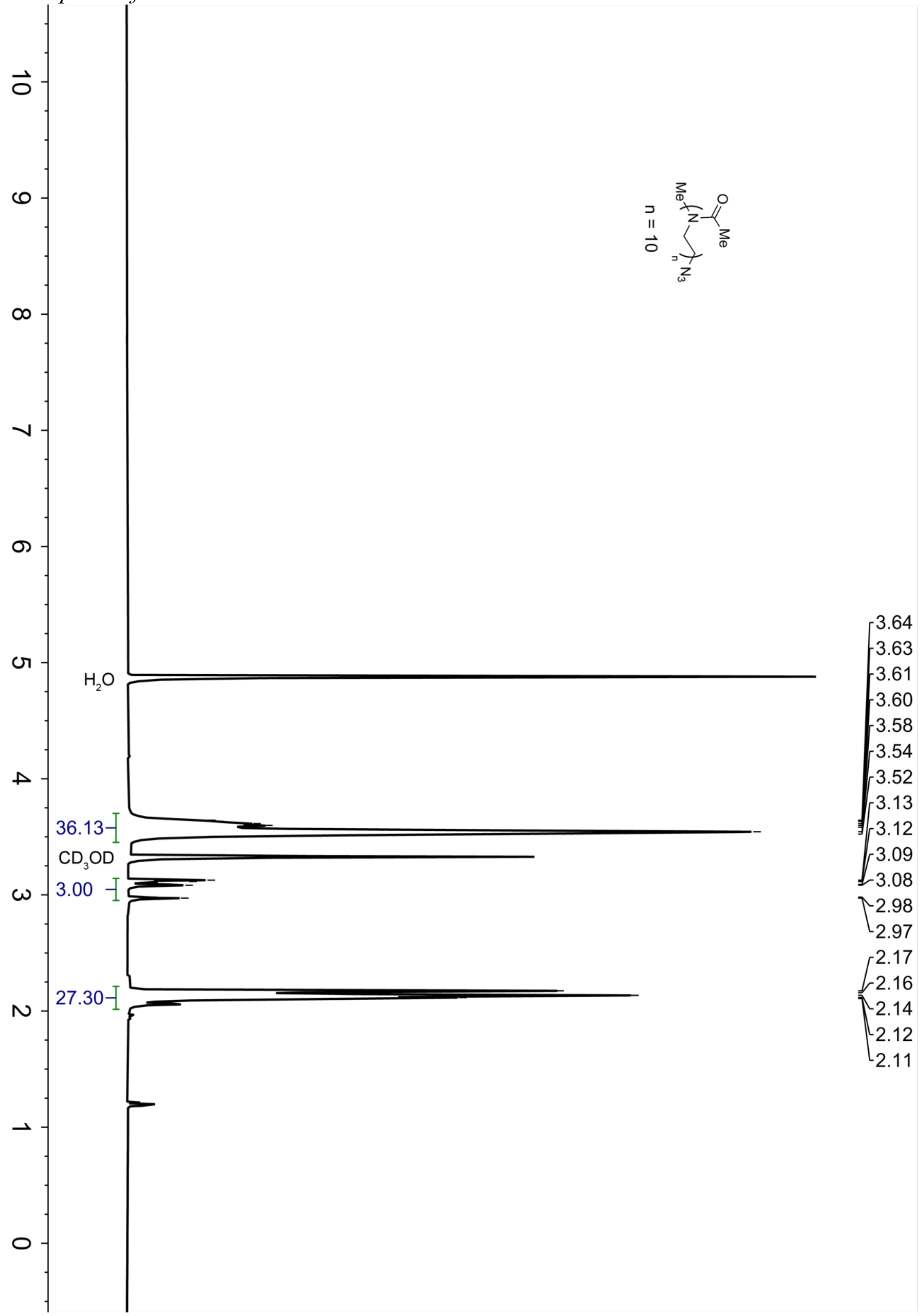

^{1}H NMR of POx, n = 10 (500 MHz, CD_3OD) δ 3.70 – 3.45 (m, 36H), 3.14 – 2.95 (m, 3H), 2.21 – 2.01 (m, 27H).

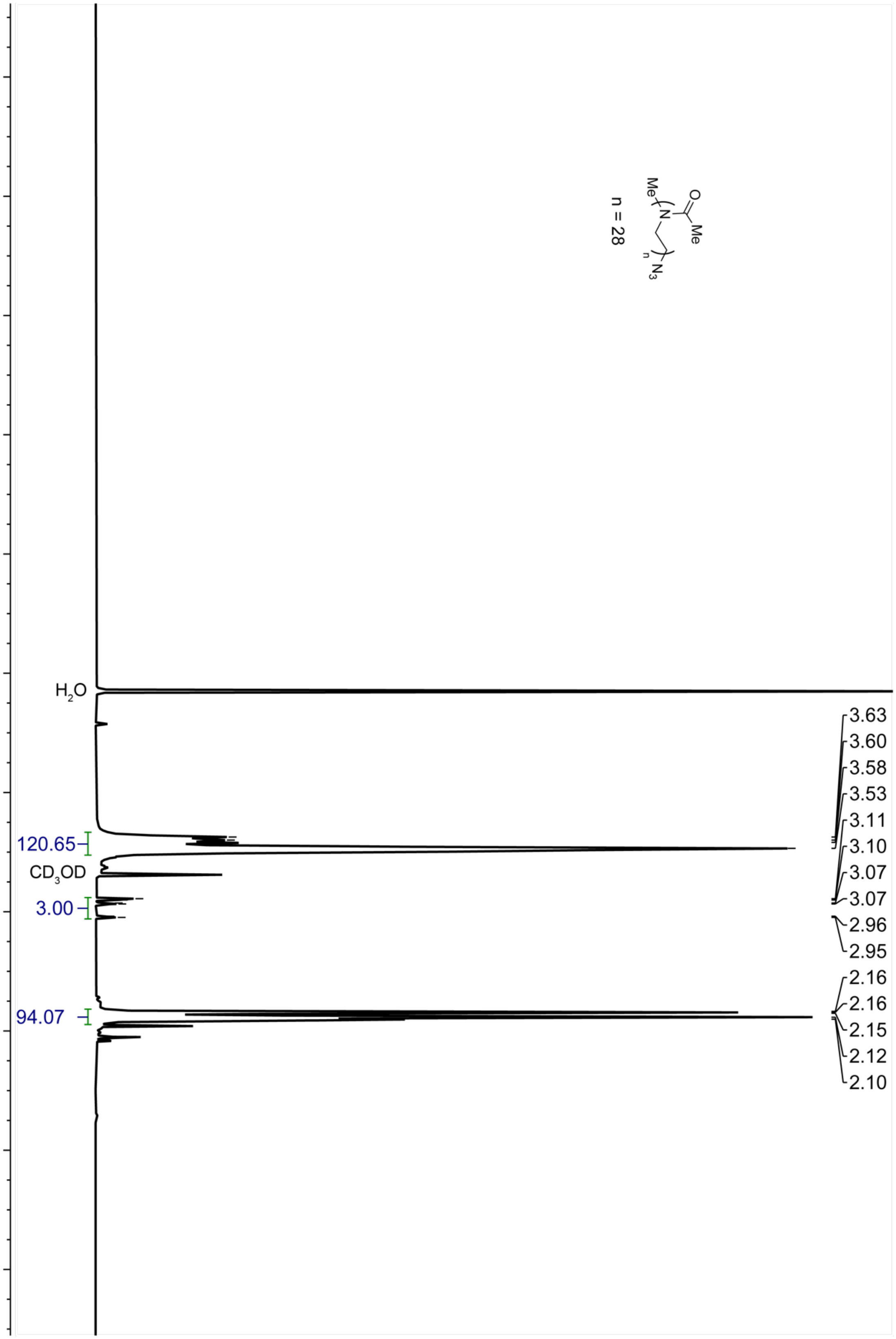

^{1}H NMR of POx, n = 28 (500 MHz, CD_3OD) δ 3.53 (s, 121H), 3.12 – 2.94 (m, 3H), 2.15 – 2.08 (m, 94H).

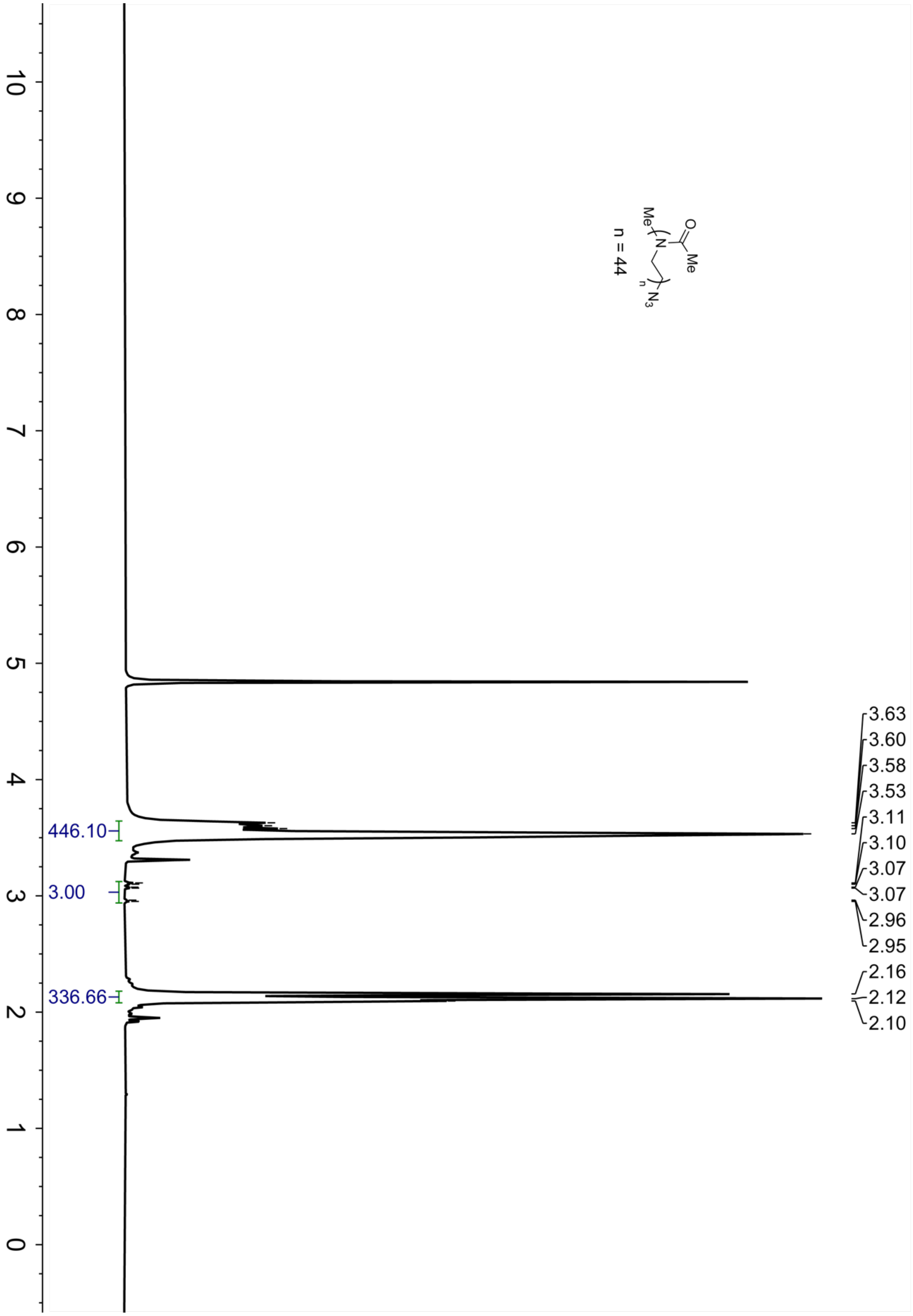

^{1}H NMR of POx, n = 44 (500 MHz, CD_3OD) δ 3.58 (d, J = 47.8 Hz, 446H), 3.12 – 2.94 (m, 3H), 2.18 – 2.08 (m, 337H). *Note*: ^{1}H NMR is not reliable for large polymer species size determination.[2]

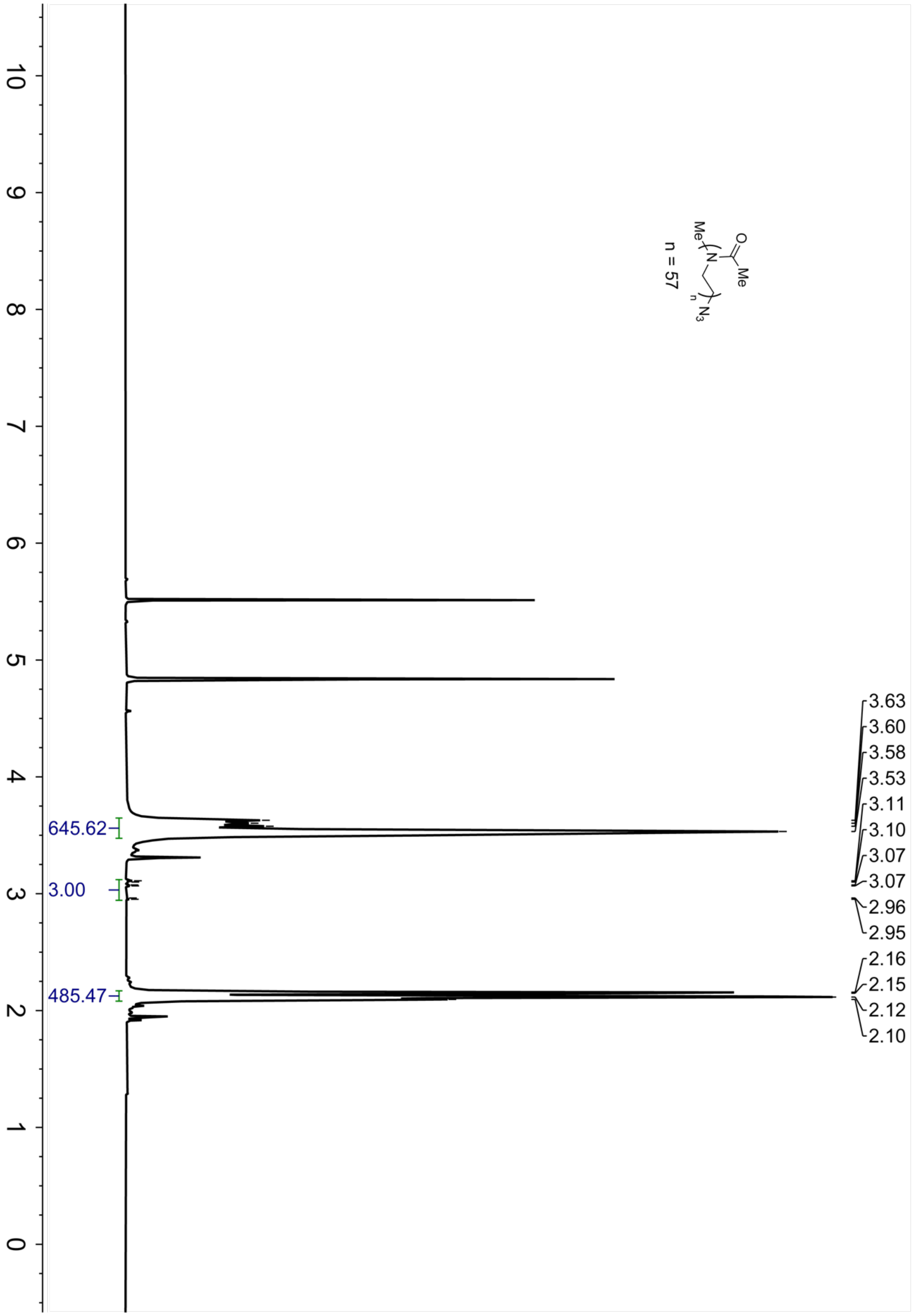

^{1}H NMR of POx, n = 57 (500 MHz, CD_3OD) δ 3.58 (d, *J* = 47.8 Hz, 646H), 3.12 – 2.94 (m, 3H), 2.17 – 2.08 (m, 485H). *Note*: ^{1}H NMR is not reliable for large polymer species size determination.[2]

Appendix B References

1. Hoogenboom, R.; Fijten, M. W. M.; Thijs, H. M. L.; Van Lankvelt, B. M.; Schubert, U. S. "Microwave-Assisted Synthesis and Properties of a Series of Poly(2-Alkyl-2-Oxazoline)s." *Des. Monomers Polym.* **2005**, *8* (6), 659-671.
2. Sedlacek, O.; Monnery, B.D.; Hoogenboom, R. "Synthesis of Defined High Molar Mass Poly(2-methyl-2-oxazoline)." *Polym. Chem.* **2019**, *10*, 1286-1290.
